# The specificity of intermodular recognition in a prototypical nonribosomal peptide synthetase depends on an adaptor domain

**DOI:** 10.1126/sciadv.adm9404

**Published:** 2024-06-19

**Authors:** Megha N. Karanth, John P. Kirkpatrick, Joern Krausze, Stefan Schmelz, Andrea Scrima, Teresa Carlomagno

**Affiliations:** ^1^ Laboratory of Integrative Structural Biology, Institute of Cancer and Genomic Sciences, College of Medical and Dental Sciences, University of Birmingham, Birmingham B15 2TT, UK.; ^2^ Institute of Organic Chemistry and Center of Biomolecular Drug Research, Leibniz University Hannover, Hannover D-30167, Germany.; ^3^ Laboratory of Integrative Structural Biology, School of Biosciences, College of Life and Environmental Sciences, University of Birmingham, Birmingham B15 2TT, UK.; ^4^ Department of Structure and Function of Proteins, Helmholtz Centre for Infection Research, Braunschweig D-38124, Germany.

## Abstract

In the quest for new bioactive substances, nonribosomal peptide synthetases (NRPS) provide biodiversity by synthesizing nonproteinaceous peptides with high cellular activity. NRPS machinery consists of multiple modules, each catalyzing a unique series of chemical reactions. Incomplete understanding of the biophysical principles orchestrating these reaction arrays limits the exploitation of NRPSs in synthetic biology. Here, we use nuclear magnetic resonance (NMR) spectroscopy and mass spectrometry to solve the conundrum of how intermodular recognition is coupled with loaded carrier protein specificity in the tomaymycin NRPS. We discover an adaptor domain that directly recruits the loaded carrier protein from the initiation module to the elongation module and reveal its mechanism of action. The adaptor domain of the type found here has specificity rules that could potentially be exploited in the design of engineered NRPS machinery.

## Teaser

An adaptor domain drives specificity of an NRPS by recruiting the donor carrier protein to the enzymatic domain.

## INTRODUCTION

Ever-increasing levels of antimicrobial resistance and the emergence of new viral threats demand the development of novel anti-infective agents. Nonribosomal peptide synthetases (NRPSs) produce small peptides as secondary metabolites in fungi and bacteria. Because of their notable structural diversity, these peptides―which contain proteinogenic and nonproteinogenic amino acids, sugar, and heterocyclic modifications―fulfill antimicrobial, antiviral, and anticancer functions (*1*). Their therapeutic potential has triggered efforts in engineering NRPS machinery to generate peptides with specific drug-like properties (*2*). However, progress in this area has been hampered by insufficient knowledge of the rules that govern intermolecular recognition in critical NRPS reaction steps.

**Fig. 1.**
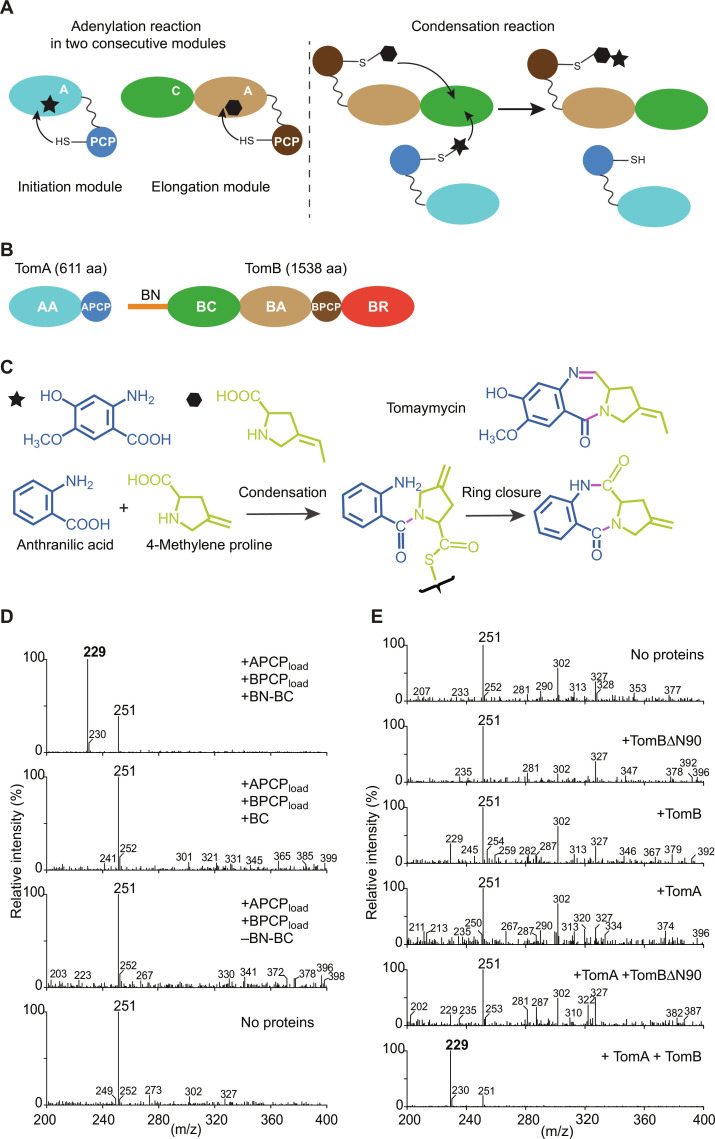
Condensation reaction of the tomaymycin NRPS. (**A**) Schematic models of prototypical initiation and elongation modules performing adenylation (left) and condensation (right) reactions. The initiation module comprises the adenylation domain A and the peptidyl carrier domain PCP. Its substrate is represented by the black star. The elongation module comprises the condensation domain C, the adenylation domain A, and the peptidyl carrier domain PCP. Its substrate is represented by the black hexagon. (**B**) Architecture of the tomaymycin NRPS: initiation module (TomA) and elongation/termination module TomB, containing the reductase domain BR. The two natural substrates and the product tomaymycin are displayed underneath. aa, amino acid. (**C**) Substrates used in this study (shown conjugated to the terminal thiol of ppant) and the products obtained after the BC-catalyzed condensation reaction and spontaneous ring closure. (**D** and **E**) LC-MS analysis of the LC peak containing the product of the condensation reaction and subsequent spontaneous ring closure, as shown in (C). The expected product mass on the M^+^ channel is 229 Da. The horizontal and vertical axes represent the mass/charge ratio (m/z) and relative abundance, respectively. The peak at 251 Da is TCEP. (D) and (E) show the reactions conducted with excised domains and full length proteins, respectively.

Linear NRPS machinery consists of a set of sequentially acting modules, which can be either individual proteins or parts of the same protein chain. Each module adds one building block (i.e., an amino acid) to the growing peptide chain and typically consists of condensation (C), adenylation (A), and peptidyl carrier protein (PCP) domains (Fig. 1A) (*3*). The A domain uses energy from adenosine triphosphate (ATP) hydrolysis to attach an amino acid subunit to the terminal thiol of a 4′-phosphopantetheine (ppant) arm coupled to a conserved serine residue in the PCP domain (PCP_ppant_) (*4*). The C domain binds two substrate-loaded PCP_ppant_ domains—termed the donor and acceptor PCPs—and transfers the growing peptide chain from the donor PCP of the upstream module onto the aminoacyl subunit carried by the acceptor PCP of the same module (Fig. 1A) (*5*). The first (initiation) module in the assembly line lacks the C domain, while the last (termination) module includes an additional domain (usually a thioesterase) to catalyze product release. Modules may also contain other catalytic domains that chemically modify the growing chain (*1*, *6*). The synthesis of nonribosomal peptides is mechanistically challenging; key to the process is the specificity of both interdomain and intermodular recognition events. Within a module, all enzymatic domains interact with each other to form a catalytic platform, with their active sites located on the same side of the platform (*7*–*10*). The core catalytic platform remains stable throughout the reaction cycle, while the acceptor PCP moves to reach the individual catalytic sites (*11*). Such communication between catalytic domains does not occur across separate modules, whose core catalytic platforms do not adopt a defined relative orientation (*11*, *12*). This leaves open the question of how specific intermodular recognition is achieved—preventing futile intermodular encounters—when individual modules are on separate protein chains.

In some, but not all, NRPSs consisting of multiple protein chains, small noncatalytic domains, referred to as docking domains (DDs), interact pairwise at the C- and N-terminal ends of two separate chains and bring them together (*13*–*20*). DDs have been exploited to facilitate intermodular communication in NRPS engineering (*17*, *21*–*23*); however, none of the DDs found to date functions to recruit the donor PCP to the C domain solely through direct interactions with the PCP globular domain (*24*).

Here, we exploit nuclear magnetic resonance (NMR) spectroscopy and mass spectrometry to discover the mechanism of intermodular recognition in the NRPS that synthesizes the cytotoxic agent tomaymycin (*25*) and reveal how the donor PCP discriminates between the two PCP binding sites on the C domain even when the condensation reaction occurs with excised domains. We discover and characterize the functional mechanism of a hitherto unknown adaptor domain—distinct from known DDs—that directly recruits the donor PCP from a separate protein chain to the correct site of the C domain.

## RESULTS

### Replication of the condensation reaction of the tomaymycin NRPS by excised functional domains

Tomaymycin is synthesized in *Streptomyces* and *Micrococci* by an NRPS consisting of the initiation module TomA and the elongation/termination module TomB (Fig. 1B) (*25*–*27*). TomA contains an A domain (AA; TomA^1–522^) followed by a PCP domain (APCP; TomA^523–610^), whose ppant arm is loaded by AA with the substrate 4-hydroxy-5-methoxyanthranilic acid. TomB contains a C domain (BC; TomB^91–533^), an A domain (BA; TomB^534–1055^), which functions to load the ppant arm of its PCP domain (BPCP; TomB^1056–1141^) with 4-ethylidene proline, and a reductase domain (BR; TomB^1142–1535^). Comparative sequence analysis (fig. S1) revealed that residues 1 to 90 of TomB (hereafter called BN) do not form part of the canonical C domain and have unknown structure and function. For our structural and functional studies, we expressed and purified all TomA and TomB domains as well as full-length (FL) TomA and TomB (see Materials and Methods). The PCP domains were coexpressed with phosphopantetheinyl transferase to ensure coupling of the ppant arm to the conserved serine and yield PCP_ppant_ domains. We used the commercially available anthranilic acid and 4-methylene proline as substrates of TomA and TomB, respectively (Fig. 1C). To confirm that the recombinant proteins are catalytically active, we mixed FL TomA and TomB (both 20 μM) with 2 mM ATP-Mg^2+^, anthranilic acid, and 4-methylene proline (both 25 μM) in buffer at pH 7.5 (see Materials and Methods) and detected the formation of compound **1** (M^+^ = 229 Da) after a 1-hour incubation using liquid chromatography–mass spectrometry (LC-MS) (Fig. 1E). **1** corresponds to the product of the NRPS except for the last reduction step, which could not occur because of the lack of the BR cofactor reduced form of nicotinamide adenine dinucleotide phosphate (NADPH). Next, we tested if we could obtain the same product with a minimal system consisting of the substrate-loaded APCP_ppant_ and BPCP_ppant_ (hereafter called APCP_load_ and BPCP_load_) and TomB^1–533^ (BN-BC), i.e., the BC domain preceded by the stretch of 90 amino acids with unknown function. AA and BA were used to achieve loading of APCP_ppant_ and BPCP_ppant_, respectively, as verified by mass spectrometry. After a 1-hour incubation, we obtained the same product (**1**) as that from the reaction performed with FL proteins (Fig. 1D). The absence of other products, such as dipeptides consisting of two 4-methylene proline units or two anthranilic acid units, demonstrated that BN-BC binds APCP_load_ and BPCP_load_ specifically at the donor and acceptor sites, respectively. As this was true for both the reactions with FL TomA and TomB and the excised domains, we concluded that the discrimination of donor and acceptor sites is intrinsic to the BN-BC–APCP_load_–BPCP_load_ complex and does not depend on the presence of the AA, BA, or BR domains.

### Structures of BN-BC and APCP

To understand the structural basis for the specificity of BN-BC–APCP_load_ recognition with excised domains, we solved the structures of isolated BN-BC, APCP_ppant_, and substrate-loaded APCP_ppant_ (APCP_load_).

#### Structure of BC

**Fig. 2.**
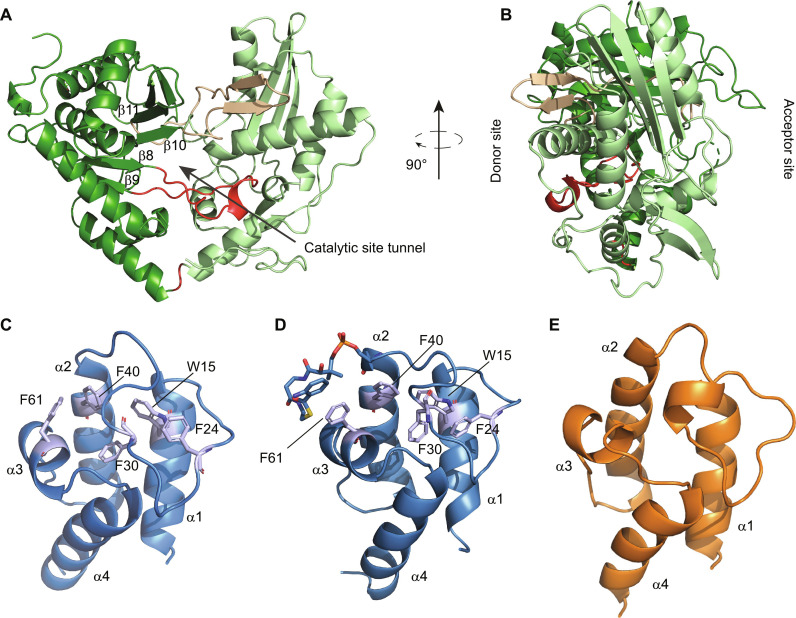
Structures of excised APCP and BN-BC domains. (**A**) Crystallographic structure of BN-BC at 1.7-Å resolution (N-form). The N- and the C-lobes are shown in light and dark green, respectively. The two cross-over elements—the floor loop and latch loop—are shown in red and beige, respectively. Also highlighted in red is the junction between the N- and C-lobes. The first 79 amino acids do not yield any electron density. (**B**) Structure of BN-BC showing the sites on opposite faces of the protein where the donor and acceptor PCPs are expected to bind. (**C**) NMR structure of APCP_ppant_ (amino acids 5 to 81). The disordered ppant arm is omitted for clarity. (**D**) NMR structure of substrate-loaded APCP_ppant_ (APCP_load_; amino acids 5 to 81) showing both the ppant arm and the substrate (in stick representation). (**E**) NMR structure of BN (amino acids 4 to 77), which closely resembles the PCP fold.

The structure of BN-BC was solved by x-ray crystallography (see Materials and Methods) in two independent crystallization experiments at resolutions of 2.3 Å (S-form, using a Se-Met derivative) and 1.7 Å (N-form) (Fig. 2, A and B, fig. S2A, and table S1). In neither form was there observable electron density for the first 79 residues. As other canonical C domains, TomB^80–533^ consists of N-terminal and C-terminal lobes, which adopt a chloramphenicol acetyltransferase fold (*28*) and are arranged in a V shape. The catalytic tunnel is at the interface between the two lobes and includes the conserved motif ^222^HHXXXD^227^, where H223 is fundamental for catalysis. Across all C domains and structurally homologous domains that perform other catalytic reactions (epimerization, cyclization, and X-domains), the relative orientation of the N- and C-lobes has been proposed to modulate the width of the catalytic tunnel and thus control access to the active site (*5*, *15*). Among other C domains, the structure of TomB^80–533^ most closely resembles that of the C domain of the linear gramicidin synthetase subunit A [LgrA; Protein Data Bank (PDB) ID 6MFZ (*11*)] (fig. S2B). The width of the aperture of the catalytic tunnel in BC is in between those of the most closed and most open C domains (fig. S2C).

In both crystal forms, the catalytic tunnel of BC is wide enough to accommodate the ppant arm and their substrates at both the donor and acceptor sites. The tunnel is ~38 Å long with a minimum width of ~1.4 Å at the bottleneck, as measured by CAVER 3.0 (*29*). In the S-form, with an empty active-site tunnel, H223-N^ε2^ faces outward, away from the tunnel; in the N-form, which has a glycerol molecule in the tunnel, H223-N^ε2^ faces inward pointing toward the glycerol (fig. S2D). This suggests that H223 may be in a noncatalytic, outward conformation in unbound BC and may switch to the catalytic, inward conformation only after binding the substrates. In previous structures, the catalytic histidine has been seen in the outward conformation only once [PDB ID 5JA1 (*30*)], where it is associated with loss of defined electron density for the entire helix α1. In comparison to all other structures of C domains, BC helix α1 is shorter, which allows the side chains of residues Y117 and N118 of the α1–β1 loop to protrude into the active site (fig. S2E).

Two crossover elements extend from the C-terminal to the N-terminal lobe: the “floor” loop between β8 and β9 and the “latch” loop between β10 and β11 (Fig. 2). The latch loop adopts a secondary structure consisting of a one-turn helix, followed by a β-hairpin (Fig. 2 and fig. S2A). The helical turn is distorted in the N-form. A β-hairpin in the latch loop has been previously observed only in a few cases [(*31*, *32*) and PDB IDs 4ZNM/4ZXW; SgcC5 protein from *Streptomyces globisporus*]. However, the latch loop of homologous proteins can adopt a variety of different secondary structures: The most conserved secondary structure element is the second β strand, sometimes preceded by a short helix; other combinations of short helical elements or no secondary structure at all have also been observed, suggesting a high degree of structural adaptability in this loop.

The crystallization construct comprised residues 1 to 533; however, the first 79 residues did not yield resolvable electron density, resulting in crystal structures of only the BC domain (91 to 533) and the preceding 11 amino acids. The stretch of 11 amino acids preceding the canonical BC domain lacks secondary structure but makes several contacts with helix α5 of the N-lobe and residues of the floor loop (fig. S2F), probably contributing to the stabilization of the relative orientation of the N- and C-lobes.

#### Structural heterogeneity of BN-BC and BC in solution

**Fig. 3.**
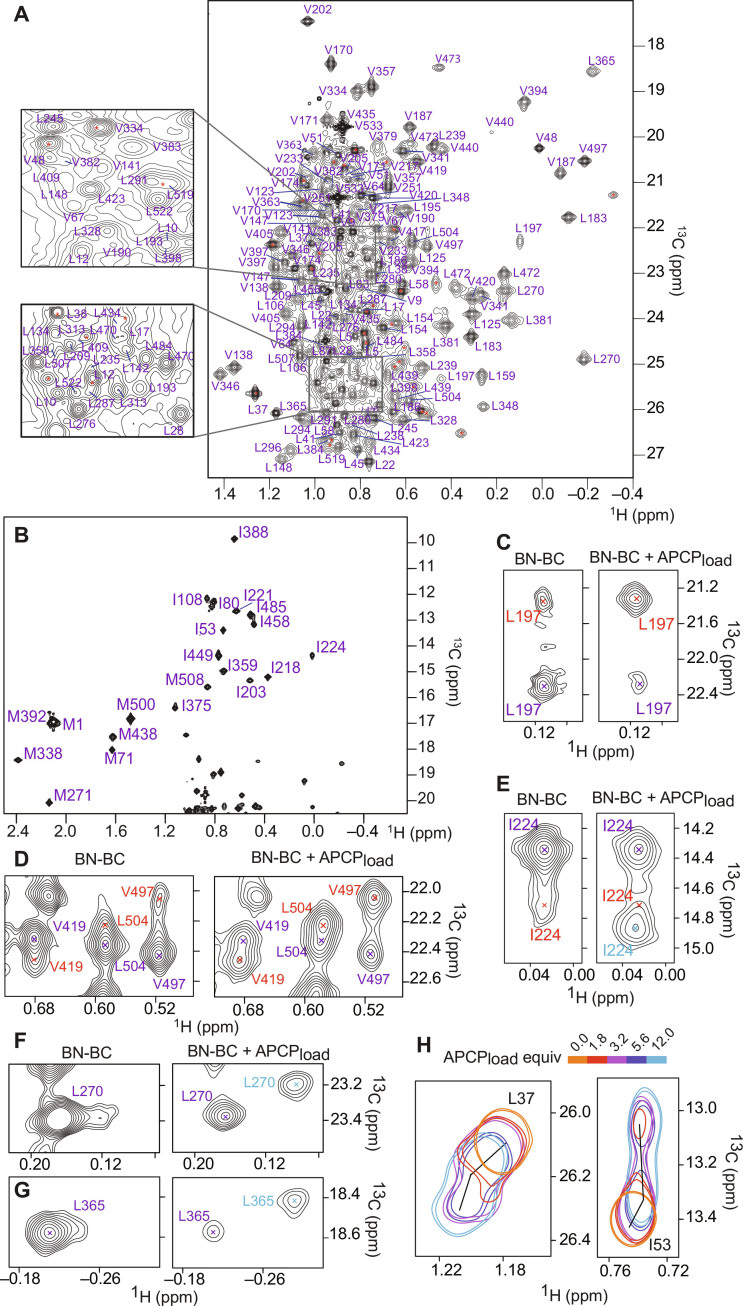
BC exhibits conformational heterogeneity. (**A** and **B**) Excerpts of the ^1^H,^13^C-methyl HMQC spectrum of 50 μM ^1^H,^13^C-ILV-methyl–labeled ^2^H-BN-BC with peak assignments. Asterisks indicate 35 unassigned peaks. ppm, parts per million. (**C** and **D**) Expansions of regions from the ^1^H,^13^C-methyl HMQC spectrum showing methyl groups that adopt two conformations in both isolated BN-BC (left) and after addition of 12 equiv of APCP_load_ (right; BN-BC at a concentration of 100 μM in both). Purple and red labels indicate the major and minor conformations, respectively. The population of the minor conformation increases in the presence of APCP_load_. (**E**) For some methyl groups, the peak corresponding to the minor conformation increases in intensity and shifts to a new position in the presence of APCP_load_ (light blue). (**F** and **G**) Some of the peaks showing little or no evidence of a second conformation in isolated BN-BC split into two peaks upon addition of APCP_load_, corresponding to the APCP-bound (light blue) and unbound (purple) forms. (**H**) Peaks belonging to the BN domain in the BN-BC construct show a bimodal behavior upon addition of 1.8 to 12 equiv of APCP_load_ to 50 μM BN-BC (spectra are color-coded according to the number of APCP_load_ equivalents).

Next, to study the formation of the BN-BC–APCP_load_ complex in solution, we assigned the peaks of the ^1^H,^13^C-methyl heteronuclear multiple-quantum coherence (HMQC) spectrum of ^1^H,^13^C-ILV-methyl–labeled ^2^H-BN-BC to the respective methyl groups. At the molecular weight of BN-BC (59 kDa), solution NMR focuses on the observation of ^1^H,^13^C methyl group resonances because their favorable relaxation properties yield tolerable linewidths even in high–molecular weight proteins. To complete the assignment, we used a four-dimensional (4D) ^13^C-HMQC–^1^H,^1^H-nuclear Overhauser effect spectroscopy (NOESY)–^13^C-HMQC experiment (*33*) and the crystallographic structure of BN-BC. Guided by the crystallographic structure, we identified groups of closely spaced ILV residues and designed 21 ILV mutants to pinpoint key residues in these clusters as starting points for assignment. In total, we assigned 84.5% of ILV-methyl groups (Fig. 3). We also used paramagnetic relaxation enhancement (PRE) NMR experiments to guide the assignment process (see Materials and Methods).

In the methyl HMQC spectrum of BN-BC, several methyl groups yielded two discernible peaks, indicating the existence of two conformational states in slow exchange with each other (Fig. 3, C to E). The residues showing two peaks were grouped into five spatial clusters, three of which correspond to pockets in the N-lobe that accommodate helix α1 and the latch and floor loops (fig. S3, A and B). The fourth and largest cluster is in the C-lobe and comprises helix α8 and the neighboring structural elements as well as α6 and α10 (fig. S3C). Because of the spatial distribution of methyl groups displaying two peaks, which cluster around the C-lobe central helix α8, we suggest that the conformational exchange is intrinsic to the tertiary structure of the C-lobe and that this plasticity is communicated to the N-lobe through the contacts between α10 and α1 and the crossover elements (floor and latch loops). It has been proposed that the latch loop is able to adopt two different conformations: a closed one, similar to that observed in our crystal structure, and an open one, where the interactions with the N-lobe are lifted and the entrance to the catalytic tunnel is wide open. The existence of an open conformation was postulated because of the need to fit the growing peptide chain in the catalytic tunnel of the C domains of NRPS systems that synthesize long peptides (*34*, *35*). In the case of TomBC, displacement of the latch loop from the N-lobe is unnecessary as the substrates and product of the condensation reaction are small enough to fit in the tunnel as it is in the crystal structure. Nevertheless, the conformational exchange observed for the methyl groups in the pocket of the N-lobe hosting the latch loop would be compatible with the existence of a second conformation of this loop that differs from that seen in the crystal structure.

The same distribution of residues with two peaks at the same chemical shifts was seen in the construct TomB^91–533^, containing the BC domain only, confirming that this conformational plasticity is an intrinsic property of BC (fig. S3, D to H). However, the population of the minor conformation was, on average, higher in BC than in BN-BC, probably because the contacts between residues BN^80–90^ and BC (fig. S2F) stabilize the major conformation in BN-BC. The presence of conformational heterogeneity in BC is consistent with a previous study that demonstrated extensive dynamics in the cyclization domain Cy1 of the NRPS that synthesizes yersiniabactin (*36*) and with the heterogeneous width of the central channel seen across the available crystal structures of C domains (fig. S2). We do not know which conformations the two NMR peaks represent, but the presence of two conformations extending from the donor to the acceptor site and involving both N- and C-lobes suggests allosteric coupling of the two PCP binding sites, similar to that observed for the cyclization domain Cy1 of HMWP2 from the yersiniabactin synthetase (*36*). Later, we will demonstrate that only one of the two conformations binds APCP_load_, which means that, in the absence of partner proteins, BN-BC populates both an inactive and an active conformation.

#### Structures of APCP

Last, we solved the structure of both APCP_ppant_ and APCP_load_ by solution-state NMR (Fig. 2, fig. S4, and table S2). APCP adopts the classical four-helix bundle fold with a long but well-ordered acidic loop connecting α1 and α2. In APCP_ppant_, a cluster of aromatic residues involving W15 and F30 of the loop, α2-F40 and α3-F61 and, peripherally, F24 and H33, stabilize both the loop conformation and the tertiary structure of the protein (Fig. 2 and fig. S4D). The C-terminal portion of the α1–α2 loop forms a short helical segment in APCP_load,_ but not in APCP_ppant_, involving four residues at positions 4 to 7 counting from the serine carrying the ppant arm (fig. S4). This short helical segment has been observed in many, but not all, PCP structures, including most crystallographic structures and a few NMR structures. The ppant arm, which shows only very few nuclear Overhauser effects (NOEs) in APCP_ppant_, becomes ordered in APCP_load_, curling back onto itself to nestle on the top of α3 (fig. S4, A and C). In this arrangement, the substrate is presented next to the serine carrying the ppant arm for recognition by BC. α3-F61 moves away from the cluster of protein aromatic residues to interact with the aromatic ring of the anthranilate substrate (Fig. 2D and fig. S4D). At the same time, F24 is stably recruited to the aromatic cluster, with H33 stabilizing the cluster from above F24 (fig. S4D).

We compared the structures of APCP_ppant_ and APCP_load_ with those of both the aryl carrier protein (ArCP) from the yersiniabactin synthetase (*37*) and the first PCP in the NRPS that synthesizes pyoluteorin (PltL) (*38*), coupled to the ppant arm in both their unloaded (ArCP_ppant_ and PltL_ppant_) and loaded (ArCP_load_ and PltL_load_) forms. In ArCP_ppant_, the 3_10_ helix is already present before substrate loading and, in both ArCP_ppant_ and PltL_ppant_, the ppant arm makes stable contacts with the protein core, in contrast to what we observe for APCP_ppant_. The core of both ArCP and PltL is formed by a cluster of aliphatic residues rather than the aromatic residues in APCP (fig. S4E). However, the aromatic residue APCP-F61 is conserved in ArCP (Y75) and, in ArCP_load_, interacts with the aromatic substrate, similar to F61 in APCP_load_. Nevertheless, the structure of the ppant arm, while curled in both ArCP_load_ and APCP_load_, differs in the two proteins (fig. S4E). In PltL_ppant_, α3 is not formed due to the presence of a glycine in what would be the middle of the helix. No aromatic residue is present at the position corresponding to APCP-F61, and the ppant arm is curled and interacts with the hydrophobic pocket between α2 and the residues corresponding to APCP-α3 independently of substrate loading.

### Dependence of the recruitment of APCP_load_ to BN-BC on the adaptor domain BN

To understand the function and structure of the first 90 amino acids of TomB, we expressed TomB^1–91^ (BN) and determined its structure by NMR (Fig. 2 and table S2). Residues 4 to 77 formed a well-defined fold consisting of a four-helix bundle that closely resembles the structure of a PCP domain. However, BN does not contain the canonical ppant attachment motif [I/L]GG[D/H]SL, which is substituted by the stretch RCEHPA, and therefore cannot function as a PCP.

As the structure did not suggest a specific function, we tested the importance of BN in the condensation reaction by repeating the functional assays with the excised domains using TomB^91–533^ (BC) rather than TomB^1–533^ (BN-BC) and the assays with the FL proteins using FL TomA and a TomB construct missing the first 90 residues (TomBΔN90) (Fig. 1, D and E). In neither case could we detect any product formation, demonstrating that BN is essential for the condensation reaction with both the excised domains and the FL modules.

Next, to verify if BN interacts with the N- and C-lobes of BC, we measured the methyl HMQC spectra of BN and BC and overlaid them with the methyl HMQC spectrum of BN-BC. The combined spectra of BN and BC superimposed very well with the spectrum of BN-BC, confirming that the BN domain adopts the same fold within BC as in isolated BN but is not an integral part of BC (fig. S5). Last, we used NMR spectroscopy to test if BN interacts with any of the APCP_ppant_ and BPCP_ppant_ domains (fig. S6). BN displayed small chemical shift perturbations (CSPs) in the presence of APCP_ppant_ but not in that of BPCP_ppant_, confirming that BN has a specific function in APCP recognition. No difference was observed in the CSPs measured in the presence of either APCP_load_ or APCP_ppant_ (fig. S6F), indicating that the APCP-BN interaction is not dependent on the substrate.

We then proceeded to test if BN is required for the recruitment of APCP to BC. We monitored the backbone amide peaks in a ^1^H,^15^N–heteronuclear single-quantum coherence (HSQC) spectrum of either APCP_ppant_ or APCP_load_ (120 μM) upon addition of either BN-BC or BC. The peaks of APCP_load_ showed substantial loss of intensity in the presence of 1.5 equiv of BN-BC (fig. S7, A and E), while the corresponding intensity decrease upon addition of 1.5 equiv of BC was much more modest (fig. S7, B and F). This confirmed that BN is essential for the recruitment of APCP to BC and thus for intermodular recognition within the NRPS.

Next, we investigated the contributions of both the ppant arm and the substrate to the strength of the BN-BC–APCP_load_ interaction. The intensity loss of APCP_ppant_ peaks upon addition of BN-BC was much smaller than for APCP_load_ (fig. S7, C and G, and table S3), indicating that the substrate contributes substantially to the affinity of APCP_load_ for BN-BC, although it plays no role in the BN-APCP interaction (fig. S6F). The affinity difference between APCP_load_ and APCP_ppant_ ensures that substrate-free APCP does not block BC and also stimulates enzyme turnover after condensation has taken place. Large loss of affinity for BN-BC was achieved only with the APCP-S37A mutant lacking the ppant arm (fig. S7, D and H, and table S3). This is in contrast to the cyclization domain–PCP interaction of the yersiniabactin synthetase (*36*) and the C domain–donor (Ar)CP interaction of the enterobactin synthetase (*39*), where the lack of substrate was sufficient to abrogate binding.

### Recruitment of APCP to BN-BC via the formation of an encounter complex with BN

To better understand the mechanism of APCP_load_ binding to BN-BC, we monitored the ^1^H,^13^C-ILV-methyl HMQC spectrum of BN-BC upon addition of an excess of APCP_load_ (Fig. 3, D to H, and figs. S8 and S9) and observed a variety of different behaviors across the methyl groups of BN-BC. The methyl groups of BC in the BN-BC construct were either unaffected by the presence of APCP_load_ or yielded new APCP-bound peaks in slow-to-intermediate exchange with the peaks of unbound BN-BC (Fig. 3, F and G). For those methyl groups that appeared in two conformations in the unbound form of BN-BC, the peak corresponding to the minor conformation either increased in intensity or both increased in intensity and shifted position (Fig. 3, C to E), suggesting that it is the minor conformation that recognizes APCP. In agreement with the hypothesis that the donor and acceptor binding sites are allosterically coupled, binding of APCP_load_ induced conformational rearrangements across the entire BC as peak changes were distributed over both lobes (fig. S9). The affinity between BN-BC and APCP_load_ is weak as, irrespective of the different behaviors described above, peaks continued changing up to addition of 12 molar equiv of APCP_load_ (fig. S8).

**Fig. 4.**
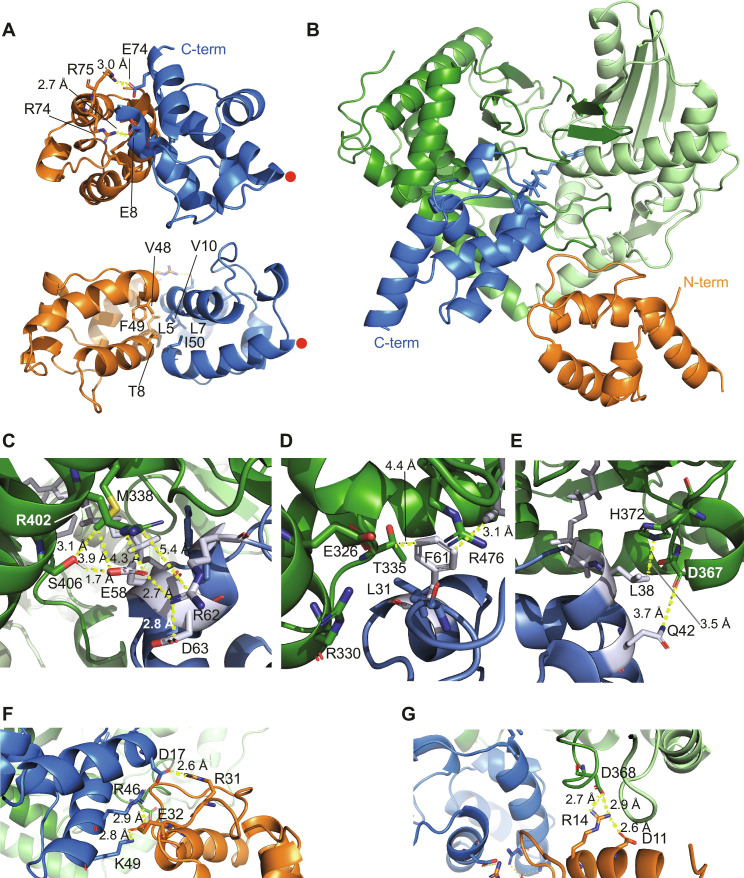
Structures of the encounter and final BN-BC–APCP_load_ complexes. (**A**) Structure of the encounter complex formed between the BN domain of BN-BC (in orange) and APCP_load_ (in blue). The ppant arm is omitted for clarity, but the red dots indicate the position of the modified serine. Hydrogen bonds and hydrophobic contacts are shown in the upper and bottom views, respectively. (**B**) Overview of the structure of the final BN-BC–APCP_load_ complex (BC, green; BN, orange; APCP_load_, blue). (**C** to **E**) Close-up views of the atomic interactions between APCP-α3 (C), APCP-F61 (D), and APCP-α2 (E) with BC. (**F** and **G**) Close-up views of the atomic interactions between BN and APCP-α1 (F) and APCP-α2 as well as BC (G).

The methyl groups of BN in the BN-BC construct showed quite a different behavior: Upon titration of APCP_load_, many of these peaks moved toward a new position in the fast exchange regime while simultaneously losing intensity and reappearing at another different position (Fig. 3H). Such a pattern of CSPs demonstrates the formation of an initial encounter complex BN-BC–APCP_load_, in fast exchange with unbound BN-BC, which then transitions to the final BN-BC–APCP_load_ complex. To determine the topology of the encounter complex, we first reasoned that the formation of this species does not involve BC because only the methyl group peaks of BN showed the pattern of CSPs characteristic of an encounter complex. Second, the interaction interfaces between BN and APCP must differ in the encounter and final BN-BC–APCP_load_ complexes as the positions of the methyl peaks of BN in the final complex did not correspond to the endpoints of the trajectories of the chemical shift changes associated with the encounter complex (Fig. 3H). It follows that the interface between BN and APCP in the encounter complex should be revealed by NMR experiments performed on mixtures of APCP and excised BN domain only. As expected, the CSPs observed for the ILV-methyl groups of BN upon addition of APCP_load_ were on the same paths as those of the CSPs attributed to the encounter complex in the ^1^H,^13^C-HMQC spectra of BN-BC upon addition of APCP_load_ (fig. S10). We thus compared the amide peaks in ^1^H,^15^N-HSQC spectra of the BN construct and APCP_ppant_ in isolation and in the presence of the other (fig. S6) and extracted the largest CSPs to guide the docking of the two domains (see Materials and Methods). We used APCP_ppant_ as the presence of the substrate did not affect the interaction of BN with APCP (fig. S6F). While the measured CSPs were very small, they consistently showed that the two domains interact at the surfaces comprising the N and the C termini of both domains (Fig. 4). In the BN-BC–APCP_load_ encounter complex, this arrangement allows BN to present the substrate-bound ppant arm, which is located on the opposite surface to the N and C termini, to the entry channel of BC (Fig. 4). The encounter complex is stabilized by two H-bonds between APCP-E8 and BN-BC-R74 and between APCP-E74 and BN-BC-R75 as well as by a cluster of hydrophobic residues containing APCP-L7, APCP-V10, and APCP-I50 and BN-BC-L5, BN-BC-T8, BN-BC-V48, and BN-BC-F49. The structure explains well the selectivity of the adaptor domain BN for APCP as, in BPCP, the positions corresponding to V10 and E74 are occupied by two arginines (fig. S1), which would cause clashes with BN-α1 and disrupt the H-bond with BN-BC-R75, respectively.

These results unambiguously attribute to the BN domain the role of recruiting APCP to the C domain of the TomB module, thus establishing BN as an NRPS adaptor domain responsible for both intermodular recognition and specificity of the condensation reaction.

### Structure of the BN-BC–APCP_load_ final complex

Because the CSPs induced by the binding of APCP_load_ to the BC core were distributed across the entire domain, they did not inform on the BC binding interface. Thus, to calculate the structure of the BN-BC–APCP_load_ final complex, we measured intermolecular distances through PRE NMR experiments. Briefly, a nitroxide tag carrying an unpaired electron (paramagnetic center) was coupled to an individual cysteine engineered on the surface of one of the proteins of the complex. The relaxation enhancements elicited by the paramagnetic center on the NMR resonances of another protein in the complex were quantified and translated into distances. To avoid complete loss of APCP_load_ peaks in the presence of BN-BC, the spectra of APCP_load_ were acquired in the presence of only 1.5 equiv of paramagnetically tagged BN-BC. Under these conditions, APCP_load_ was not saturated with BN-BC, which means that the resulting PREs were scaled down by the fraction of bound APCP_load_. The methyl HMQC spectra of BN-BC were acquired in the presence of 2.5 molar equiv of paramagnetically tagged APCP_load_, also yielding a mixture of BN-BC and BN-BC–APCP_load_ complex (table S4; see Materials and Methods). Consequently, the distances extracted from the quantification of the PREs were necessarily larger than in the complex; hence, distance restraints for the structure calculation were constructed using the PRE-derived distances as upper bounds and with no lower bounds. Of the 12 paramagnetic tags engineered on BC as part of BN-BC, only 3 gave quantifiable PRE effects, demonstrating that APCP_load_ binds to a well-defined surface of BC (figs. S11 and S12; see Materials and Methods).

First, we used the PRE data measured between BC in the BN-BC construct and APCP_load_ to dock APCP_load_ to BC and obtained a well-defined structure (Fig. 4, B and C). The APCP four-helix bundle engages only with the C lobe of BC, mostly through its α3 helix. APCP-F61 protrudes toward the BC surface and is sandwiched between BN-BC-R476, BN-BC-T335, and the ppant arm in a hydrophobic cavity protected by a “roof,” formed by BN-BC-R476, E326, and R330 together with APCP-L31 (Fig. 4D). Most of the APCP residues interacting with BC are not conserved in BPCP (Fig. 4, C to E, and fig. S1C).

To verify the structure, we monitored the decrease in peak intensities of wild-type or mutant APCP upon addition of 1.5 equiv of either wild-type or mutant BN-BC (table S3). The mutant APCP_load_-F61S bound BN-BC with much reduced affinity, corroborating its interaction with the BC donor binding site. Furthermore, in free APCP_load_, F61 stacks with the donor substrate and localizes it to a well-defined position next to APCP-S37 (Fig. 2D). Localization of the substrate prior to binding reduces the entropy penalty that would incur if the substrate and the ppant arm were to enter the catalytic tunnel of BC from a highly disordered state, thus increasing the affinity between APCP_load_ and BC. Affirming the importance of an aromatic residue at the position of APCP-F61 for the recognition of donor PCPs carrying aromatic substrates, the aromatic residue is conserved in other NRPS systems with aryl substrates (*40*) but is absent in BPCP.

Next, we used PREs measured between APCP_load_ and the BN domain in the BN-BC construct to dock BN to the BC–APCP_load_ structure. The measured PREs stemmed from both the encounter and the final BN-BC–APCP_load_ complex; hence, to obtain a structural model that is representative solely of the final complex, we excluded all PRE effects compatible with the encounter complex. This resulted in only eight PRE restraints measured between APCP_load_-D17C and residues 2 to 77 of BN-BC (table S4) and one PRE restraint measured between BN-BC-A36C and residue 7 of APCP_load_ (fig. S13). Because of the low number of restraints, the docking calculation did not converge on one individual structure but yielded a few clusters. Nevertheless, the lowest energy cluster is clearly distinguishable from the others and yields the best fit to the experimental data (Fig. 4B and fig. S14). Most intermolecular interactions occur between the loop α1–α2 of BN and APCP-α1 and are electrostatic in nature (Fig. 4, F and G). Mutants APCP_load_-K49V and BN-BC-D11A each interacted with their wild-type counterparts with reduced affinity (table S3), in agreement with the contributions of these residues at intermolecular interfaces in the BN-BC–APCP_load_ final complex.

The structure of BN-BC bound to APCP_load_ differs slightly from those of other condensation, condensation-like, and cyclization domains in complex with their cognate carrier protein at the donor site (*10*–*12*, *41*). While, in all other structures, the relative positions of the carrier proteins and the C-lobes of the condensation (or homologous) domains are similar, in the BN-BC–APCP_load_ complex, APCP is rotated by ~30° and moved slightly away from BC (fig. S15, A and B). This positions the C^α^ atom of the serine carrying the ppant arm at a distance of 7 Å from that of the other structures (fig. S15C). Nevertheless, the flexibility of the ppant arm allows the substrate to penetrate the enzymatic tunnel equally well as in all other structures. These differences are not unexpected given that, in the structures determined so far, the carrier protein is part of the same protein chain as the C domain, and thus its orientation may be guided by the linker conformation, contacts, and length. Furthermore, the presence of the BN domain in our structure may guide APCP to a slightly different position with respect to complexes lacking the adaptor domain.

## DISCUSSION

**Fig. 5.**
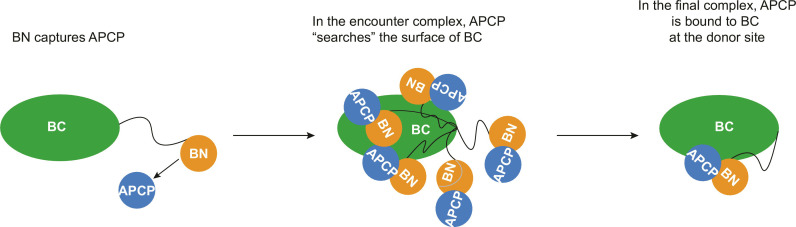
Schematic model of APCP recognition by BN-BC. BN encompasses a folded PCP-like domain, which recruits APCP from solution through formation of an encounter complex. Because of the flexibility provided by the disordered region corresponding to amino acids 78 to 90, the APCP bound to BN can scan the surface of BC. Once APCP finds the donor binding site, an array of specific (but individually weak) interactions commits APCP to this binding site. BN readjusts its position to optimize the interaction interfaces and yield the final complex.

For an NRPS assembly line to proceed efficiently, the selectivity of each enzymatic center for its intended substrates needs to be coupled with rapid dissociation of the products. This requirement precludes the PCP domains from harboring strong and selective interaction interfaces with their cognate C domain as this would lead to slow turnover. Here, we reveal that the tomaymycin NRPS uses an array of weak interactions, including the formation of a transient but specific encounter complex, to ensure correct intermodular recognition. The combination of multiple weak interactions can be finely modulated to achieve either specific recognition or product dissociation at different reaction steps. We demonstrate that the condensation reaction of the tomaymycin NRPS occurs specifically even with excised domains, provided that the PCP-like adaptor domain BN is present on the C domain. The formation of the BN–APCP complex is the first step of the recognition of APCP by either FL TomB or the truncated TomB^1–533^. The BN–APCP interaction is unique to the donor PCP, providing a mechanism for specific intermodular recognition. The flexible linker between the BC domain and the adaptor domain at its N terminus allows APCP to explore the surface of BC while bound to BN in the encounter complex and thus find the donor binding site (Fig. 5). The presentation of the aromatic substrate next to APCP-S37, together with the specific interactions of APCP-α3 with BC, commits APCP to the donor binding site (Fig. 5). The interactions between the APCP four-helix bundle and the BC C-lobe are specific but weak as, by themselves, they do not suffice to recruit APCP_load_ to BC (fig. S7H).

Among the known DDs that mediate the intermodular interaction between the donor PCP and the C domain (*14*–*18*, *20*), the driving contacts involve the DD upstream of the condensation (or condensation-like) domain and regions C-terminal to the globular PCP domain, rather than the domain itself. For example, βHD domains primarily recognize disordered regions [short linear motifs (SLiMs)] downstream of the donor PCP globular domain (*15*, *17*, *18*, *20*), while in peptide‐antimicrobial‐Xenorhabdus (PAX) multiprotein NRPS, the DD primarily recognizes a short helical motif (<35 amino acids) C-terminal to the PCP domain (*14*, *16*). In our case, the interaction between APCP and BN-BC does not depend on the disordered residues at the C-terminal end of APCP as their deletion has no impact on the interaction of APCP_load_ with BN-BC (APCP_load_ΔC11 in table S3). In addition, BN remains bound to APCP during catalysis. Hence, we have designated BN as an adaptor rather than a DD.

In summary, we have found an intermodular communication domain in an NRPS system that directly mediates interaction between the C domain and the donor PCP, a development that may present opportunities for the modular design of nonnatural NRPSs. The identity of the side chains presented on the surface of the adaptor domain could be optimized to recognize specific PCPs, different from the one of TomA, thus offering a possible strategy to tune affinity and specificity of donor PCP recognition in engineered systems.

## MATERIALS AND METHODS

### Protein preparation

The plasmid constructs for the proteins AA, APCP, BA, BPCP, BC, and BN-BC were cloned into pETM-44 with a cleavable N-terminal hexa-histidine tag and a maltose-binding protein (MBP) tag for solubility enhancement (gift from R. Mueller, HIPS Saarbrucken, Germany). The plasmid constructs for FL TomA and TomB (*26*) and for TomBΔN90 were cloned into pSTW42 with a cleavable hexa-histidine tag and a small ubiquitin-like modifier (SUMO) tag for solubility enhancement. The BN construct was made by inserting a stop codon into the BN-BC plasmid.

Single-point, double-point, and deletion mutants of APCP, BN and BN-BC were made by site-directed mutagenesis using standard protocols and the QuikChange II site-directed mutagenesis kit from Agilent Technologies.

Cells containing the plasmid of interest were grown in *Escherichia coli* BL21(DE3), induced with 0.1 mM isopropyl β-D-1-thiogalactopyranoside (IPTG) at an OD_600_ (optical density at 600 nm) of 0.6 and subsequently grown at 16°C for 18 hours. Proteins that carry the phosphopantetheinyl arm (ppant) were coexpressed with the MtaA ppant transferase (*42*). Clarified cell lysates in buffer containing 50 mM tris(hydroxymethyl)aminomethane (Tris) and 1 M NaCl at pH 8.0 (buffer A) were applied to HisTrap 5-ml columns (GE Healthcare Life Sciences) equilibrated in the same buffer and eluted with a linear gradient from 0 to 300 mM imidazole in 5 to 10 column volumes. The tag was then cleaved by overnight treatment with His-HRV3C protease at 4°C in buffer A (1:100 protease:substrate w/w ratio). A final purification step was carried out by size exclusion chromatography (SEC; using Superdex S75 pg 16/60 or Superdex S200 pg 16/60) either in storage buffer containing 50 mM Tris (pH 7.5), 150 mM NaCl, 5% v/v glycerol, and 0.02% w/v NaN_3_ or in NMR buffer containing 50 mM sodium phosphate (pH 7.0), 150 mM NaCl, 0.5 to 1 mM tris(2-carboxyethyl)phosphine (TCEP), and 0.02% w/v NaN_3_.

To prepare APCP_load_, APCP_ppant_ at a concentration of 50 to 100 μM was incubated overnight at 25°C with 0.2 to 1 molar equiv of AA in storage or NMR buffer and supplemented with 5 mM ATP, 5 mM MgCl_2_, 2 mM TCEP, and 4 mM substrate A (anthranilic acid; Sigma-Aldrich). The product APCP_load_ was purified by SEC using an analytical S200 column (Superdex 200 Increase 10/300 GL, GE Healthcare Life Sciences) in the appropriate buffer at 4°C. Substrate loading was nearly complete as verified by intact LC-MS of purified APCP_load_. Furthermore, substrate loading was monitored by both NMR and ultraviolet (UV) spectroscopy. With NMR, we monitored the chemical shifts of the ppant amide and methyl groups, which differed substantially between APCP_ppant_ and APCP_load_. With UV spectroscopy, we measured the UV absorption of the loaded anthranilate substrate at a λ_max_ of 310 nm.

Uniformly ^15^N-labeled samples were prepared by using ^15^NH_4_Cl (1 g/l) as the sole nitrogen source in M9 minimal medium. Similarly, ^15^N,^13^C-labeled samples were made by using ^15^NH_4_Cl (1 g/l) and ^13^C_6_-d-glucose (2.5 g/l) as the sole nitrogen and carbon sources, respectively. Selectively ILV-methyl–labeled samples, i.e., samples containing (^1^H,^13^C-γ methyl)-valine, (^1^H,^13^C-δ methyl)-leucine, and (^1^H,^13^C-δ1 methyl)-isoleucine in an otherwise perdeuterated background, were prepared by addition of the ILV precursors—[3-^13^C; 3,3-^2^H_2_]-2-ketobutyrate and [3-^13^C; 3,4,4,4-D_4_]-2-ketoisovalerate (Cambridge Isotope Laboratories)—at concentrations of 60 and 120 mg/mL, respectively, 1 hour prior to induction. Selectively IMLV-methyl–labeled samples were made as above with addition of [^13^C; 2,3,3,4,4-^2^H_5_]-methyl-l-methionine (NMR-Bio) 1 hour prior to induction. MV[pro-S] (^1^H,^13^C-ε1 methionine and ^1^H,^13^C- γ2 valine) and LV[pro-R] (^1^H,^13^C-δ1 leucine and ^1^H,^13^C-γ1 valine) were prepared according to the manufacturer’s protocol (NMR-Bio).

Individual protein domains—AA, APCP, BN, BN-BC, BC, BPCP, and BA—were checked for their viability for structural studies. The purity, oligomeric and aggregation states, maximum concentration in solution, correct fold, and stability were assessed by SEC, mass spectrometry, SDS–polyacrylamide gel electrophoresis (PAGE), and NMR (1D ^1^H and 2D ^1^H,^15^N correlation experiments). Furthermore, the solubility and stability of these constructs were assessed and optimized by high-throughput screening of various buffer conditions via thermal shift assays (*43*). A consensus buffer, in which all the proteins were reasonably stable and soluble, was chosen as the final “NMR buffer” to perform the studies. Those proteins that were not entirely stable in the NMR buffer were prepared in the assay buffer and buffer-exchanged into the NMR buffer immediately before the NMR experiments. In all cases, proteins used for structural studies were prepared fresh, kept at 4°C, and not subjected to freeze-thaw cycles.

### Activity assays

The assays were carried out in storage buffer supplemented with 2 mM ATP, 2 mM MgCl_2_, and 1 mM TCEP (assay buffer). For the activity assays performed with the excised domains, BPCP_ppant_ was incubated with 250 μM substrate B (4-methylene proline) and BA at a 1:1 BPCP_ppant_:BA ratio for 20 min at 25°C to obtain BPCP_load_. This solution was then mixed with (i) APCP_load_ (representing the control mixture); or (ii) APCP_load_ and BN-BC; or (iii) APCP_load_ and BC. An additional control mixture consisted of the assay buffer and both substrates A and B. The mixtures were incubated for 1 hour at 25°C for the condensation reaction to proceed. All proteins were kept at equimolar ratio, each at a final protein concentration of 100 μM in a reaction volume of 100 μl.

For the activity assays using FL Tom proteins, TomA and either TomB or TomBΔN90 were incubated with their respective substrates for 1 hour at 25°C in assay buffer. These reactions were then mixed in two combinations: (i) TomA and TomB; and (ii) TomA and TomBΔN90. The final mixtures were incubated for another hour at 25°C for the condensation reaction to proceed. All proteins were kept at equimolar ratio, each at a final concentration of 20 μM and with substrate concentrations of 25 μM each. Three mixtures were used as controls: (i) TomA in assay buffer and substrate; (ii) either TomB or TomBΔN90 in assay buffe r and substrate A; and (iii) assay buffer with substrates A and B.

The SDS-PAGE images of all proteins used in the assays are shown in fig. S16.

The condensation reactions were stopped by addition of 1.5 volumes of ice-cold acetonitrile. The samples were then freeze-thawed and centrifuged at high speed to remove the precipitated proteins. Samples were probed for the presence of the product by mass spectrometry using an analytical LC-MS instrument consisting of a Waters mass-directed autopurification system (Waters 2767 autosampler and Waters 2545 pump attached to a C18 column (Phenomenex Kinetex 2.6 μm, C18, 100 Å, 4.6 mm by 100 mm), a photodiode array detector in the range of 210 to 600 nm (Waters 2998, Photodiode Array Detector), and an electrospray ionization mass detector in the detection range of 100 to 1000 m/z (Waters, SQ Detector 2). A linear gradient program of 15-min duration, transitioning from a mixture of 10% acetonitrile/90% water (with 0.05% formic acid) to 90% acetonitrile/10% water (with 0.05% formic acid) at a flow rate of 1 ml/min was used. The sample volume per run was 20 μl. The resulting data were analyzed using the software MassLynx (Waters).

### Design of mutants for PRE experiments and conditions for the spin labeling reaction

The surface accessibility of native cysteine residues in BN-BC was assessed by the program NACCESS (http://bioinf.manchester.ac.uk/naccess/); C32 was identified as surface-exposed and therefore mutated to serine. Starting from this construct (C32S), 15 single-point mutants with a single surface-exposed cysteine residue were generated (A36C, A47C, S102C, D132C, S155C, A185C, A229C, E269C, T277C, A320C, T331C, D396C, S406C, S450C, and S478C). The sites were chosen such that all surface regions of BC could be probed for interaction with APCP.

APCP does not have any native cysteine residues. Residues were selected on each of the main helices to generate three single-point cysteine mutants (D17C, S45C, and E67C).

The tag carrying the unpaired electron (spin label) was coupled to the one exposed cysteine residue on the protein of interest. In the case of APCP_load_, the loading reaction was carried out prior to spin labeling. The spin label used was 3-(2-iodoacetamido)-proxyl radical (Sigma-Aldrich). Pure protein samples at ~0.1 mM were reduced with up to 100-fold excess of dithiothreitol (DTT) for several hours. DTT was then removed by rapid buffer exchange (via a desalting column) into 50 mM Tris and 150 mM NaCl at pH 8.0. The samples were immediately treated with 3- to 10-fold molar excess of the spin label and incubated overnight in the dark at 16°C. Unreacted spin label was removed by exchange into buffer B (50 mM sodium phosphate, 150 mM NaCl, and 0.02% w/v azide, pH 7.0) via a desalting column. After the acquisition of the NMR experiments with the sample in the paramagnetic state, the tag was reduced by addition of ascorbic acid to a final concentration of 10 mM and then measured in the diamagnetic state.

A list of all mutant constructs generated in this work is provided in table S5.

### Sequence alignment

Sequences were aligned using Clustal Omega or EMBOSS Needle (*44*) and formatted using ESPript 3.0 (*45*). For the BC domain (fig. S1B), the protein BC^1–533^ was aligned against the seed sequences for the C domain family in the PFAM entry PF00668 (*46*).

### X-ray crystallography

BN-BC structure determination followed standard protocols using SeMet derivative crystals for experimental phasing and native crystals to build the final structure. Briefly, initial crystallization conditions were identified with automated procedures using the sitting-drop vapor diffusion method and then optimized by grid screening. All crystallization experiments were conducted at room temperature. For SeMet crystals, growth occurred in 100 mM Tris (pH 8.5) and 3.0 M potassium formate. Native crystals were grown in 100 mM Tris (pH 8.5) and 3.5 M sodium formate. The crystallization solution contained BN-BC (30 mg/ml) in 50 mM potassium phosphate buffer (pH 7.0) and 150 mM NaCl. All crystals were grown at 4°C and cryo-protected with 25% w/v glycerol prior to flash freezing. Diffraction data were collected at 100 K. SeMet derivative data were collected on beamline 14.1 at BESSY (Berlin, Germany) and native data on beamline ID 29 at ESRF (Grenoble, France).

Data processing was conducted using AutoPROC (*47*) (Global Phasing), utilizing XDS (*48*), STARANISO (*49*), Pointless (*50*), and Aimless (*51*). The structure of BN-BC was solved through single anomalous dispersion using data collected at the selenium absorption edge at 0.9797 Å. Initial phases were calculated using AutoSol (*52*), and a partial model was generated by running AutoBuild (*53*), both components of the PHENIX software package (*54*). The output model was analyzed in Coot (*55*) and manually corrected to obtain a reliable search model for subsequent molecular replacement with Phaser (*56*) against the dataset of the native BN-BC crystal. The final structural model was built through iterative rounds of manual building in Coot (*55*) and crystallographic refinement with Phenix.refine from the PHENIX software suite (*54*). Data collection and refinement statistics are summarized in table S1.

### NMR spectroscopy

NMR spectra were recorded on 600- and 850-MHz Bruker Avance III HD spectrometers, equipped with cryogenic inverse HCN 5-mm probe heads (N_2_- and He-cooled, respectively), and running Bruker Topspin software (v3.2). All spectra were recorded at a temperature of 298 K. Samples were dissolved in NMR buffer [50 mM sodium phosphate (pH 7.0), 150 mM NaCl, 0.5 to 1 mM TCEP, and 0.02% w/v NaN_3_], which was made with either pure D_2_O (for methyl-detected experiments) or a 90:10 H_2_O:D_2_O mixture (for amide-detected experiments). Protein concentrations ranged from 50 to 800 μM depending on the proteins and the experiments to be recorded, and four different types of NMR sample tubes were used (3 and 5 mm in diameter standard tubes and 3 and 5 mm in diameter Shigemi microtubes). A variety of different isotope-labeling schemes were used, as described below, but all samples for methyl-detected experiments (except for BN, which was uniformly ^13^C,^15^N-labeled) were prepared with selective methyl group ^13^C-labeling and 100% deuteration at all side-chain positions apart from the methyl groups to be detected.

### Methyl group NMR spectroscopy

2D ^1^H-^13^C correlation spectra of methyl-labeled samples were recorded with an HMQC-based pulse sequence to exploit the methyl TROSY effect (*57*). For methyl HMQC spectra requiring the highest resolution, the pulse sequence incorporated a purge element to explicitly suppress residual contributions from the rapidly relaxing coherences to the final 2D line shape (*58*). Typical acquisition times (*t*_1,max_) were 80 to 100 and 40 to 60 ms in the ^1^H and ^13^C dimensions, respectively.

Assignment of the Ile-δ1, Leu-δ1/δ2, Val-γ1/γ2, and Met-ε1 methyl groups was done manually by collating information from a collection of different NMR experiments, which were analyzed in conjunction with the atomic-resolution structure of BN-BC determined by x-ray diffraction. In brief, the process was initiated from a subset of “anchor point” assignments obtained by careful inspection of the methyl HMQC spectra recorded on a small number of single-point mutants and the two BN-BC subdomains (BN and BC). The assignment was then expanded from these anchor points by analysis of inter-methyl NOESY spectra, guided by supplementary information derived from intra-BC PRE measurements, stereo- and residue-specifically labeled samples, and J coupling–based methyl–to–side chain correlation experiments.

The following methyl-methyl NOESY spectra were recorded (all at 850 MHz): 3D HCH-NOESY [^13^C-HMQC–NOESY pulse sequence (*59*, *60*)], 3D (H)CCH-NOESY [^13^C-HMQC–NOESY–^13^C-HMQC pulse sequence (*61*, *62*)], and 4D HCCH-NOESY [^13^C-HMQC–NOESY–^13^C-HMQC pulse sequence with diagonal peak suppression (*33*)] on an IMLV[rac]-labeled sample and 4D HCCH-NOESY on an LV[rac]-labeled sample ([rac] denotes racemic labeling of the prochiral LV methyl groups). The 3D NOESY spectra were recorded with uniform sampling in all dimensions. The 3D HCH-NOESY spectrum was recorded with double semiconstant time evolution in the ^1^H indirect dimension and four scans per increment; the NOESY mixing time and recycle delay were set to 0.4 and 1.0 s, respectively, and the *t*_1,max_ values were 25 and 40 ms for the indirect ^13^C and ^1^H dimensions, respectively. The 3D (H)CCH-NOESY spectrum was recorded with eight scans per increment; the mixing time and recycle delay were set to 0.4 and 0.8 s, respectively, and the *t*_1,max_ values for the two indirect ^13^C dimensions were both 25 ms. The 4D HCCH-NOESY spectra were recorded with sparse nonuniform sampling (NUS) in all indirect dimensions. To improve the quality of NUS reconstruction, both 4D HCCH-NOESY spectra were recorded with diagonal peak suppression (*33*). Briefly, diagonal peak suppression was implemented by acquiring two datasets in an interleaved fashion: the first with the standard pulse sequence (“cross-peak” dataset) and the second with a modified pulse sequence that generates only diagonal peaks (“diagonal-only” dataset). The final diagonally suppressed NOESY spectrum is generated by first subtracting the diagonal-only dataset from the cross-peak dataset and then proceeding with NUS reconstruction in the usual manner. The 4D HCCH-NOESY spectrum on the LV[rac]-labeled sample was generated by recording the two datasets with eight scans per increment, a mixing time of 0.45 s (for both the cross-peak and diagonal-only datasets), and a recycle delay of 0.6 s. The *t*_1,max_ values were 20 ms for the two indirect ^13^C dimensions and 19 ms for the indirect ^1^H dimension. The nominal NUS sparsity was 0.55%, but the spectral widths in the indirect dimensions were set to approximately twice the respective chemical shift dispersions in those dimensions (22 ppm in the two indirect ^13^C dimensions and 4 ppm for the indirect ^1^H dimension). This translates to an underlying Nyquist grid with twofold oversampling in all three dimensions, for which the number of hypercomplex points is a factor of 8 (2^3^) greater than in the corresponding nonoversampled grid that would be used for the equivalent uniformly sampled dataset (with the same *t*_1,max_ values in all dimensions). The effective NUS sparsity was therefore 0.55% × 2^3^ = 4.4% (35% per indirect dimension). The degree of diagonal peak suppression for this spectrum was optimized in an empirical fashion by applying a scaling factor (1.45) to the diagonal-only dataset prior to subtraction from the cross-peak dataset. The 4D HCCH-NOESY spectrum on the IMLV[rac]-labeled sample was also recorded using eight scans per increment, mixing times of 0.4 and 0.28 s for the cross-peak and diagonal-only datasets, respectively, and a recycle delay of 0.6 s. The *t*_1,max_ values were 19 ms for the two indirect ^13^C dimensions and 20 ms for the indirect ^1^H dimension. The nominal NUS sparsity was 0.21%, but the spectral widths in the indirect dimensions were again set to approximately twice the respective chemical shift dispersions in those dimensions (39 ppm in the two indirect ^13^C dimensions and 6 ppm for the indirect ^1^H dimension), so the effective NUS sparsity was 1.7% (26% per indirect dimension). In this case, no scaling was applied to the diagonal-only dataset prior to subtraction from the cross-peak dataset.

NUS schedules were generated according to the Poisson gap sampling method, using the online schedule generator available on the nus@HMS web server (http://gwagner.med.harvard.edu/intranet/hmsIST/gensched_new.html). Reconstruction of uniformly sampled time-domain data matrices was achieved using the approach of iterative soft thresholding, as implemented in the program hmsIST (*63*). Subsequent processing of the reconstructed data was performed with NMRPipe (v10.1) (*64*). All spectra were analyzed in CcpNMR Analysis (v2.4) (*65*).

Three intra–BN-BC methyl PRE datasets were used to aid methyl group assignment. The PRE tags were attached to BN-BC S102C, A185C, and S406C point mutants. These PREs were measured in the same way as the interprotein PREs used to provide restraints for the structure determination of the BN-BC–APCP_load_ complex (vide infra).

Residue-type identification and stereospecific assignment of the diastereotopic (prochiral) methyl groups of Leu and Val were assisted by inspection of the methyl HMQC spectra recorded on samples of BN-BC prepared with the following terminal ^1^H,^13^C-methyl labeling schemes: LV[rac] (^1^H,^13^C-Leu-δ1/δ2 and ^1^H,^13^C-Val-γ1/γ2; racemically labeled methyl groups), IMLV[rac] (^1^H,^13^C-Ile-δ1, ^1^H,^13^C-Met-ε1, ^1^H,^13^C-Leu-δ1/δ2, and ^1^H,^13^C-Val-γ1/γ2; racemically labeled Leu and Val methyl groups), MV[pro-S] (^1^H,^13^C-Met-ε1 and ^1^H,^13^C-Val-γ2), and LV[pro-R] (^1^H,^13^C-Leu-δ1 and ^1^H,^13^C-Val-γ1).

Anchor point assignments were obtained by inspection of the methyl HMQC spectra recorded on samples of BN-BC, BC, and BN (the last two constructs corresponding to TomB residues 91 to 533 and residues 1 to 91, respectively) and those of the following BN-BC single-point mutants: I53A, I80A, I104A, I107A, L159A, L183A, V187A, I221A, L270A, L328A, V334A, V363A, L365A, L381A, I388A, V394A, L409A, I449A, I485A, V497A, and L504A. Samples of the point mutants were prepared with either Ile-δ1 labeling (for the Ile mutants) or LV[rac] labeling (for the Leu and Val mutants). The sample of BC was also prepared with LV[rac] labeling, and that of BN was uniformly protonated and ^13^C-labeled. The 2D ^1^H-^13^C correlation spectrum of BN was therefore recorded with a constant-time HSQC sequence (*66*).

The identification of methyl group pairs belonging to the same Leu and Val residue was done with the assistance of a 3D through-bond experiment in which the ^1^H and ^13^C resonances of the methyl groups were correlated with the ^13^C resonances of the directly bonded methine carbon (C^γ^ and C^β^ for Leu and Val, respectively), similar to an experiment previously described by Sprangers and Kay (*67*). This experiment was recorded on an LV[rac]-labeled sample with full-chain ^13^C labeling with an in-house pulse sequence comprising an out-and-back magnetization transfer pathway starting and ending on the methyl protons, using correlation spectroscopy–type transfers between the methyl and methine carbons and constant-time chemical shift evolution periods for both indirect ^13^C dimensions.

In the end, only 12 of 70 Leu, 4 of 41 Val, and 1 of 14 Ile of BN-BC remained unassigned.

### Titrations

^15^N-HSQC spectra (*68*–*70*) of 0.10 to 0.15 mM uniformly ^15^N-labeled BN in NMR buffer (50 mM potassium phosphate, 150 mM NaCl, 5 mM DTT, and 0.02% w/v NaN_3_, pH 7.0) were recorded at 298 K in the absence and presence of either APCP_ppant_ or BPCP_ppant_ (1:2.5 molar ratio). ^13^C-HSQC and ^15^N-HSQC spectra of uniformly ^13^C,^15^N-labeled BN were recorded in the absence and presence of 5 molar equiv of APCP_load_.

^15^N-HSQC spectra of 0.08 mM APCP_ppant_ in NMR buffer were recorded in the absence and presence of BN (1:2 molar ratio). In addition, ^15^N-HSQC spectra of 0.12 mM APCP_ppant_ or APCP-S37A were recorded in the absence and presence of BN-BC (1:1.5 molar ratio). ^15^N-HSQC spectra of APCP_load_ and mutants APCP_load_-ΔN5, APCP_load_-ΔC11, APCP_load_-F61S, and APCP_load_-Q13V/K49V in NMR buffer at a concentration of 0.12 mM were recorded in the absence and presence of either BN-BC or BC (1:1.5 molar ratio).

^13^C-HSQC spectra of ^1^H,^13^C-ILV-methyl–labeled ^2^H-BN-BC at a concentration of 0.05 mM in NMR buffer were recorded in the absence and presence of 1.8, 3.2, 5.6, and 12 molar equiv of APCP_load_.

Proteins for each titration point were prepared separately by diluting from aliquots of a concentrated stock. The same buffer stocks used for the final SEC of the individual proteins were used for the preparation of the respective NMR samples. For each titration point, relative concentrations of APCP were further verified by comparing the peak intensities in 1D ^1^H NMR experiments in the range of 2 to 5 ppm; for BN-BC, the peak intensity of V533 (C-terminal residue of BC) was used. Translational diffusion coefficients were determined for samples at the starting point and endpoints of the titration series. The samples at the endpoint of the titrations were also analyzed by SEC.

CSPs were calculated according to the formula

$$\text{CSP}=\sqrt{{\left(∆\delta_{H}\right)}^{2}+{\left(0.15\times ∆\delta_{N}\right)}^{2}}$$ (1)

where Δδ_H_ and Δδ_N_ are the ^1^H and ^15^N chemical shift differences between the peaks in the ^15^N-HSQC spectra recorded in the absence and presence of the binding partner.

### Structures of APCP_ppant_, APCP_load_, and BN

2D ^15^N-HSQC spectra were recorded with a standard ^15^N-HSQC pulse sequence (*68*) using WATERGATE with water flip-back for the suppression of the water signal (*69*, *70*). 2D ^1^H-^13^C correlation spectra for side-chain assignment were recorded as ^13^C-HSQCs with both real-time and constant-time ^13^C chemical shift evolution periods (*66*). Backbone resonance assignments were obtained from a standard suite of 3D triple-resonance out-and-back–type spectra, comprising HNCO (*71*), HN(CA)CO (*72*, *73*), HNCA and HNCACB (*74*, *75*), and HN(CO)CA and HN(CO)CACB (*76*, *77*) spectra. The side-chain assignments were obtained through H(CCCO)NH, (H)CC(CO)NH (*78*, *79*), HBHA(CBCACO)NH and TOCSY-HSQC (*80*), and HC(C)H-TOCSY (*81*, *82*) experiments. Aromatic assignments were aided by analysis of (HB)CB(CGCD)HD and (HB)CB(CGCDCE)HE spectra (*83*). 3D NOESY–^15^N-HSQC (*60*, *84*) and 3D NOESY–^13^C-HSQC spectra (*59*, *85*) were recorded with a mixing time of 120 ms. Samples were in NMR buffer at a concentration of 0.5 to 0.8 mM. All NMR data were processed with NMRPipe and hmsIST (*63*, *64*) and analyzed in CcpNmr Analysis v2.4 (*65*).

For APCP_load_, the assignments were transferred from the ^15^N- and ^13^C-HSQCs of APCP_ppant_ to APCP_load_ and further confirmed by analysis of 3D NOESY–^15^N-HSQC and 3D NOESY–^13^C-HSQC spectra. For both APCP_ppant_ and APCP_load_, the assignment of the ppant arm was achieved by analysis of HC(C)H-TOCSY and NOESY spectra.

Backbone dihedral angle were predicted either by DANGLE (*86*) within CcpNmr Analysis using experimentally observed backbone ^1^H^N^, ^1^H^α^, ^13^C^α^, ^13^C′, ^15^N, and side-chain ^13^C^β^ chemical shifts (for APCP_ppant_ and BN) or by TALOS-N (*87*) using experimentally observed backbone ^1^H^N^, ^1^H^α^, ^13^C^α^, ^15^N, and side-chain ^13^C^β^ chemical shifts for APCP_load_. Dihedral angle predictions labeled “strong” and “generous” according to TALOS-N classification were used as dihedral angle restraints. The error bounds for the restraints were calculated by doubling and tripling the errors estimated by TALOS-N with minimum errors of 20° and 30° for strong and generous predictions, respectively.

Structure calculation was performed with the software package Ambiguous Restraints for Iterative Assignment (ARIA) v2.3 (*88*) using Crystallography and NMR System (CNS) v1.21 (*89*). NOE cross-peaks were assigned manually in all dimensions wherever possible. Otherwise, the resonance in the ^1^H dimension corresponding to the nonheteronuclear-edited proton was left unassigned. In regions of extensive overlap, all peak dimensions were left unassigned for the auto-assignment protocol within ARIA. Distance restraints were generated within ARIA using the relaxation matrix calibration protocol. For the modified residues ppant and ppant-substrate, the CNS directories were modified to include the topology, linkage, and parameter files [initially generated with the PRODRG server (*90*) and further modified]. All cross-peaks were used. A total of nine iterations and an additional water refinement step were used to generate a final ensemble of 10 structures (table S2).

### Paramagnetic relaxation enhancements

Except where stated otherwise, all PRE measurements were made using a “single time point” approach, wherein a standard 2D correlation spectrum was measured first on the sample containing the nitroxide group of the PROXYL tag in its paramagnetic state and then repeated on the same sample after reduction with ascorbic acid to generate a diamagnetic hydroxylamine group. The PREs were then extracted from the intensity ratios of the peaks in the paramagnetic and diamagnetic spectra, as described in detail below. The measured PREs were converted into distance restraints for the structure calculations. Intrasubunit PREs of BN-BC were used to derive an estimate for the electron–nucleus correlation time and to assist in the assignment of the BN-BC methyl groups.

Intersubunit PREs were measured on the amide groups of APCP_load_ in the presence of spin-labeled BN-BC. Spin labels were coupled individually to the following BN-BC mutants: A36C, A47C, D132C, S155C, A185C, A229C, E269C, T277C, A320C, T331C, D396C, S406C, S450C, and S478C. 2D ^1^H-^15^N correlation spectra of APCP_load_ were measured with a standard ^15^N-HSQC pulse sequence (*68*) using WATERGATE with water flip-back for the suppression of the water signal (*69*, *70*). APCP_load_ was present at a concentration of 0.1 to 0.12 mM and BN-BC at a molar ratio of 1:1.5. The total length of the fixed delays within the magnetization transfer pathway during which ^1^H magnetization is transverse was 10.3 ms. Intersubunit PREs were also measured on the methyl groups of BN-BC (at a concentration of 0.08 mM) in the presence of spin-labeled APCP_load_ (at a molar ratio of 1:2.5). Spin labels were coupled individually to the following APCP mutants: D17C, S45C, and E67C. 2D ^1^H-^13^C correlation spectra of BN-BC were recorded with the standard ^13^C-HMQC pulse sequence (no purge element was applied for PRE measurements). The total length of the fixed delays within the magnetization transfer pathway during which ^1^H magnetization is transverse was 7.7 ms.

Intrasubunit PREs of BN-BC were measured in 2D ^1^H-^13^C correlation spectra recorded with the standard ^13^C-HMQC pulse sequence. Three sets of intra–BN-BC PREs were acquired, corresponding to spin labels attached to the point mutants S102C, A185C, and S406C. The A185C and S406C PREs were measured with the single time point protocol described previously, while those for S102C were measured using a two time point approach, where the datasets corresponding to the paramagnetic and diamagnetic samples are each measured as a two-plane pseudo-3D ^1^H-*R*_2_ relaxation experiment. The pulse sequence used for these datasets was based on the ^13^C-HMQC pulse sequence, modified to incorporate a variable relaxation delay and also including the element to purge contributions from fast-relaxing coherences (*91*). The relaxation delays for the two 2D planes were set to 0 and 10 ms.

In the single time point approach, extraction of PREs from the ratios of peak heights in the paramagnetic and diamagnetic spectra requires knowledge of the diamagnetic transverse relaxation rates for the coherences present during the chemical shift evolution periods of the two dimensions of the 2D spectrum. Therefore, the diamagnetic transverse relaxation rates $R_{2,\text{dia}}^{H}$ and $R_{2,\text{dia}}^{\text{HC}-\text{MQ}}$ were measured for the methyl groups of wild-type, diamagnetic BN-BC using an IMLV[rac]-labeled sample. $R_{2,\text{dia}}^{H}$ rates were measured using the same pulse sequence as that used for the two time point PRE measurements, but without the purge element. The relaxation delays used were 0, 2, 5, 10, 15, 20, 30, and 40 ms. $R_{2,\text{dia}}^{\text{HC}-\text{MQ}}$ rates were measured using a pulse sequence from Tugarinov and Kay (*92*). The relaxation delays used were 0, 2, 5, 10, 15, 20, 25, and 30 ms.

All PRE and diamagnetic relaxation spectra were processed with NMRPipe (v10.1) (*64*). PRE spectra were processed with pure exponential apodization in both dimensions, which is mandatory for extraction of the PREs using the single time point approach in combination with peak height intensity ratios. After processing, peak volumes and linewidths of both PRE and diamagnetic relaxation spectra were extracted using the line shape fitting software FuDA (https://ucl.ac.uk/hansen-lab/fuda/); where peak heights were required for the subsequent analysis, these were back-calculated from the fitted volumes and linewidths. For the diamagnetic relaxation spectra, FuDA was also used to extract the relaxation rates by fitting the exponential decay curves.

The PRE effects were quantified as follows. The transverse PRE for any spin I is denoted $\Gamma_{2}^{I}$ . The additional contribution to the time-dependent decay of transverse proton magnetization due to the PRE is given by the factor $e^{-\Gamma_{2}^{H}t}$ so that the overall decay due to transverse relaxation is described by the term $e^{-\left(R_{2,\text{dia}}^{H}+\Gamma_{2}^{H}\right)t}$ , where $R_{2,\text{dia}}^{H}$ is the diamagnetic proton transverse relaxation rate. In the two time point approach (used for intra-BC methyl PREs measured with the PRE tag at S102C), the overall relaxation rate $R_{2,\text{dia}}^{H}+\Gamma_{2}^{H}$ can be extracted from the intensity ratio of the peaks in the two planes for the paramagnetic sample, *I*_ratio,para_ = *I*_para_(τ_2_)/*I*_para_(τ_1_), where τ_1_ and τ_2_ are the two relaxation delays (τ_2_ > τ_1_), and the corresponding intensity ratio for the diamagnetic sample, *I*_ratio,dia_. The PRE is given by

$$\Gamma_{2}^{H}=\frac{-1}{\Delta \tau }\text{ln}\left(\frac{I_{\text{ratio},\text{para}}}{I_{\text{ratio},\text{dia}}}\right)$$ (2)

where Δτ = τ_2_ − τ_1_. The equation remains the same if peak volumes are used instead of peak intensities.

For the single time point approach—provided the peak volumes in the paramagnetic (*V*_para_) and diamagnetic (*V*_dia_) spectra can be measured accurately (used for inter-PREs measured on amides of APCP)—the PRE is given by

$$\Gamma_{2}^{H}=\frac{-1}{\Delta }\text{ln}\left(\frac{V_{\text{para}}}{V_{\text{dia}}}\right)$$ (3)

where Δ is the total length of the fixed delays within the magnetization transfer pathway during which ^1^H magnetization is transverse.

If, instead, peak heights are measured (all PREs measured on methyls of BC, except for the S102C intra-PREs), then the expression for the peak height ratio, $\frac{I_{\text{para}}}{I_{\text{dia}}}$ , of peaks in the paramagnetic and diamagnetic methyl HMQC spectra can be written as

$$\frac{I_{\text{para}}}{I_{\text{dia}}}=e^{-\Gamma_{2}^{H}\cdot \Delta }\frac{\left(R_{2,\text{dia}}^{\text{HC}-\text{MQ}}+\pi \cdot {\text{LB}}_{F1}\right)\cdot \left(R_{2,\text{dia}}^{H}+\pi \cdot {\text{LB}}_{F2}\right)}{\left(R_{2,\text{dia}}^{\text{HC}-\text{MQ}}+\pi \cdot {\text{LB}}_{F1}+\Gamma_{2}^{\text{HC}-\text{MQ}}\right)\cdot \left(R_{2,\text{dia}}^{H}+\pi \cdot {\text{LB}}_{F2}+\Gamma_{2}^{H}\right)}$$ (4)

where $\Gamma_{2}^{\text{HC}-\text{MQ}}$ is the PRE for the ^1^H/^13^C MQ coherence present during the indirect evolution time and ${\text{LB}}_{F1}$ and ${\text{LB}}_{F2}$ are the line broadening factors (in hertz) applied for the exponential apodization of time-domain data in the indirect (F1) and direct (F2) dimensions, respectively. $\Gamma_{2}^{\text{HC}-\text{MQ}}$ can be expressed as the sum of the transverse PREs on the methyl protons and methyl carbon, i.e., $\Gamma_{2}^{\text{HC}-\text{MQ}}=\Gamma_{2}^{H}+\Gamma_{2}^{C}$ . If it is assumed that the distances to the paramagnetic center and order parameters of the corresponding nucleus–electron vectors are the same for the protons and the carbon nucleus of any methyl group and if it is further assumed that all contributions to the respective spectral density functions at nonzero frequencies are negligible relative to the contribution at zero frequency, then $\Gamma_{2}^{C}$ can be approximated as $\Gamma_{2}^{C}\approx {\left(\frac{\gamma_{C}}{\gamma_{H}}\right)}^{2}\cdot \Gamma_{2}^{H}$ and hence

$$\Gamma_{2}^{\text{HC}-\text{MQ}}\approx \left[1+{\left(\frac{\gamma_{C}}{\gamma_{H}}\right)}^{2}\right]\cdot \Gamma_{2}^{H}=1.063\;\Gamma_{2}^{H}$$ (5)

The final equation relating the single time point, methyl HMQC peak height ratio to the methyl proton PRE is

$$\frac{I_{\text{para}}}{I_{\text{dia}}}=e^{-\Gamma_{2}^{H}\cdot \Delta }\frac{\left(R_{2,\text{dia}}^{\text{HC}-\text{MQ}}+\pi \cdot {\text{LB}}_{F1}\right)\cdot \left(R_{2,\text{dia}}^{H}+\pi \cdot {\text{LB}}_{F2}\right)}{\left(R_{2,\text{dia}}^{\text{HC}-\text{MQ}}+\pi \cdot {\text{LB}}_{F1}+1.063\;\Gamma_{2}^{H}\right)\cdot \left(R_{2,\text{dia}}^{H}+\pi \cdot {\text{LB}}_{F2}+\Gamma_{2}^{H}\right)}$$ (6)

This equation is noninvertible with respect to $\Gamma_{2}^{H}$ . Methyl PREs were extracted numerically from peak height ratios using a collection of in-house MATLAB scripts. Uncertainties in the extracted PREs were estimated from the uncertainties in the experimentally measured parameters ( $I_{\text{para}}$ , $I_{\text{dia}}$ , $R_{2,\text{dia}}^{H}$ , and $R_{2,\text{dia}}^{\text{HC}-\text{MQ}}$ ) using a Monte Carlo approach implemented in MATLAB.

$\Gamma_{2}^{H}$ values were translated into structural restraints as follows. The Solomon-Bloembergen equation for the ^1^H transverse PRE, $\Gamma_{2}^{H}$ , elicited by the nitroxide spin label can be written as

$$\Gamma_{2}^{H}=\frac{\kappa_{H}}{r_{eN}^{6}}\left[4\cdot J_{\text{SBMF}}\left(0\right)+3\cdot J_{\text{SBMF}}\left(\omega_{H}\right)\right]$$ (7)

where *r*_*e*N_ is the electron–nucleus distance, *J*_SBMF_(ω) is the Solomon-Bloembergen model-free (SBMF) spectral density at angular frequency ω (radians per second), and κ_H_ is given by

$$\kappa_{H}={\left(\gamma_{H}g_{e}\beta \right)}^{2}\cdot {\left(\frac{\mu_{0}}{4\pi }\right)}^{2}\cdot \frac{S\left(S+1\right)}{15}=1.2311\times 10^{16}{Å}^{6}s^{-2}$$ (8)

in which physical constants are denoted by their usual symbols and *S* is the electronic spin quantum number ( $S=\frac{1}{2}$ for nitroxide radicals). The full form of *J*_SBMF_(ω) is

$$J_{\text{SBMF}}\left(\omega \right)=\frac{S^{2}\tau_{1}}{1+\omega^{2}\tau_{1}^{2}}+\frac{\left(1-S^{2}\right)\tau_{t}}{1+\omega^{2}\tau_{t}^{2}}$$ (9)

τ_1_ is the electron–nucleus correlation time accounting for global rotational diffusion (mean correlation time $=\tau_{m}$ ) and longitudinal electron relaxation (time constant $=T_{1e}$ ): $\tau_{1}^{-1}=\tau_{m}^{-1}+T_{1e}^{-1}$ . $\tau_{t}$ is the corresponding correlation time describing the contribution to the electron–nucleus correlation function from internal motion (correlation time $=\tau_{i}$ ) of the electron–nucleus vector in the molecular frame: $\tau_{t}^{-1}=\tau_{1}^{-1}+\tau_{i}^{-1}$ . $S^{2}$ is the (squared) order parameter for the corresponding internal motion. If the timescale of the internal motion is fast compared to the rotational diffusion and the electronic relaxation rate and the order parameter is not too small ($S^{2}$> 0.5), then the second term in *J*_SBMF_(ω) can be neglected. With this simplification, the expression for $\Gamma_{2}^{H}$ becomes

$$\Gamma_{2}^{H}=\frac{\kappa_{H}}{r_{eN}^{6}}S^{2}\tau_{1}\left(4+\frac{3}{1+\omega^{2}\tau_{1}^{2}}\right)$$ (10)

This was the expression used to convert the measured intersubunit PREs into initial nucleus-electron distances, which were then converted into distance restraints for High Ambiguity Driven Biomolecular Docking (HADDOCK) structure calculation runs. The squared order parameter $S^{2}$ was set to 1.0, and the electron–nucleus correlation time τ_1_ was set to 20 ns.

The value of 20 ns for the electron-nucleus correlation time was derived by fitting the intra–BN-BC methyl PREs measured for the S406C mutant to the crystal structure of BN-BC via the molecular dynamics–based protocol of Iwahara *et al.* (*93*), as implemented in Xplor-NIH (*94*) using scripts derived from templates written by N. Anthis. This protocol optimizes an ensemble of 10 tag conformers in combination with the electron–nucleus correlation time. The PRE potential was set to SBMF mode, with the following parameter settings: rlxType = “r2dd,” tcType = “opt,” sbmfType = “taut,” tTtype = “fix,” and tauT = 0. These settings correspond to PREs mediated by the dipolar mechanism and the form of the SBMF spectral density function given by Eq. 9. The electron-nucleus correlation time was optimized in the range of 5 to 40 ns. The final optimized value was 19.2 ± 1.1 ns.

### Determination of the structure of the encounter complex

As starting structures, we used the five lowest energy conformers of the solution structures of APCP_load_ and BN. The residues of the disordered N- and C-terminal tails of both proteins, which did not contribute to the interactions, were removed for simplicity, as well as the ppant-substrate element. The structures therefore composed of APCP_load_^5–76^ and BN^3–75^.

Residues showing a CSP greater than the average by at least 1 SD, in NMR experiments where one component of the complex was mixed with the other, were chosen as interacting residues. Interacting residues that had a relative surface accessibility of >15% (as calculated by the program NACCESS) and formed a continuous surface on the proteins were chosen to generate ambiguous interaction restraints (AIRs) in the docking software HADDOCK v2.4 (*95*). In HADDOCK, interacting residues are classified as either “active” or “passive.” AIRs are generated between any atom of active residues of protein A and any atom of both active and passive residues of protein B with a maximum value of 2.0 Å. We defined as active and passive those residues showing CSPs greater than the average by at least 1.5 SD and 1 SD, respectively. Residues of protein A whose resonances became weak or lost intensity upon addition of the protein B and were located close to active residues of protein A were classified as passive. In total, we identified six and seven active residues for APCP (amino acids 5, 9, 10, 52, 67, and 72) and BN (amino acids 6 to 8, 49, 50, 73, and 74), respectively. The number of passive residues was nine for APCP (amino acids 6, 8, 11, 50, 51, 70, and 73 to 75) and four for BN (amino acids 3 to 5 and 75).

In the first step of the protocol (rigid body docking; it0) 10,000 structures were calculated, of which 400 were carried forward to the second stage (semiflexible refinement in torsion angle space; it1) and then to the final refinement in explicit water (in Cartesian space). All 25 combinations of the five starting structures of each protein were used in it0. In it1, residues at the interface (defined as being within 5 Å of the other component) were defined as flexible by HADDOCK. The final water-refined structures were clustered according to their interface ligand root mean square deviation (il-RMSD) with a cutoff of 2.0 Å and ranked according to the HADDOCK score. The cluster with the lowest HADDOCK score was chosen as the final structural ensemble.

### Determination of the structure of the final BN-BC–APCP_load_ complex

As we have no structural information about the linker joining BC with the folded domain of BN in the BN-BC–APCP_load_ complex, we used the two individual components (i.e., BC and BN^2–77^) rather than FL BN-BC in the docking calculations. The sequence of BC was further reduced to eliminate both flexible ends and comprised amino acids 94 to 525. As starting structures for APCP_load_ and BN, we used the lowest energy conformers from the solution-state structural ensembles solved by NMR (Fig. 2). The residues of the disordered N- and C-terminal tails were removed for simplicity to yield APCP_load_^6–80^ and BN^2–77^, respectively. As starting structures for BC, we used the two crystallographic structures with H223 directed inward, i.e., in the catalytic conformation. The loop 428 to 432, which was missing from all experimental structures, was rebuilt using Modeller (*96*). Starting structures were protonated at N^δ1^ of histidine 135, 188, 223, 282, 351, 411, and 520 and at N^ε2^ of histidine 222, 308, 316, 372, 451, 464, 482, and 506, according to predictions from MolProbity (*97*).

The PRE effects measured for BN-BC and APCP_load_ were quantified and translated into distances, as explained above. Only PRE intensity ratios lower than 0.8 were considered. For PRE effects observed on BN-BC, some methyl groups showed one individual peak (or two partially overlapping peaks) while others showed two distinct peaks representing the unbound and APCP_load_-bound states. For consistency, when the two peaks were distinct, the ratio *I*_para_/*I*_dia_ was calculated as the ratio of the sum of the intensities of unbound and APCP_load_-bound peaks.

For PRE effects observed on APCP_load_ and BN-BC, distance restraints were given between the amide nitrogen of APCP_load_ and the C^β^ carbon of BN-BC and between the C^β^ carbon of APCP_load_ and the methyl carbon of BN-BC, respectively. To account for the length of the tag and for errors in the quantification of the PRE effects, an empirical error bound of 6 Å was added to the extracted distance value. The distance calculated from the PRE effect was given as upper bound with no lower bound to account for the fact that—because the observed protein is never completely saturated with its binding partner—the apparent distance derived from the PREs is necessarily larger than the actual distance in the complex.

The PRE effects observed for amino acids 2 to 77 of BN-BC (i.e., in the BN domain) due to interactions with spin-labeled APCP_load_ could stem from both the encounter and final complexes. To avoid contamination of PRE effects stemming from the encounter complex, we eliminated all PRE-derived restraints that could be satisfied in the encounter complex (6 of 14). Furthermore, few and sparse PRE effects stemming from either spin-labeled APCP-D17C or S45C mutants were observed on α1, β2, β4, and loop α3–β5 of the N-lobe for exposed ILV residues. These effects were incompatible with all the others. Because of their diffuse nature, which involved a large surface on BC, they cannot stem from a specific interaction but are rather the result of a process where APCP_load_ explores the accessible surface of BC in the encounter complex (Fig. 5). Thus, these PREs were not considered in the structure calculation of the final complex.

The PRE effects observed for APCP_load_ amide groups due to interactions with BN-BC spin-labeled in the BN domain could also stem from both the encounter and final complexes. To avoid contamination of PRE effects stemming from the encounter complex, we eliminated all PRE-derived restraints that could be satisfied in the encounter complex, such as all data for the A47C mutant (fig. S13). In the presence of spin-labeled BN-BC-A36C mutant, PRE effects were observed within the N-terminal tail of APCP_load_. These PREs were represented with a single restraint between the C^β^ carbon of A36 and the amide nitrogen of L7 at the end of helix α1 as the N-terminal tail is not present in the structure of APCP_load_ used to calculate the structure of the BN-BC–APCP_load_ complex.

In total, for the calculation of the final complex, we used 67 and 9 PRE-derived distance restraints to define the position and orientation of APCP_load_ relative to BC and BN, respectively.

To ensure that BN^2–77^ and BC remain at a distance that is compatible with the length of the linker BN-BC^78–94^, we restrained the distance between the C^α^ of the C-terminal residue of BN^2–77^ and the C^α^ of the N-terminal residue of BC to a maximum distance of 59.5 Å.

Last, two kinds of synthetic restraints were applied to the loaded ppant arm—one to “unfurl” the arm from the starting curled conformation adopted in the APCP_load_ structures (distance between the ppant thiol S and the phosphate P atoms set to 14.0 to 16.5 Å) and the other to make the arm reach the active site of BC (distance between the ppant thiol S atom and the BC-H223 C^ε1^ atom set to a maximum of 8.0 Å). These restraints were introduced in the it1 stage of the HADDOCK calculations and were not used during the rigid body energy minimization stage (it0).

The structural ensemble of the final BN-BC–APCP_load_ complex was calculated in two stages. We first determined the structure of the BC component with APCP_load_ and then docked the BN component to this complex. For the first step, two-body docking of BC and APCP_load_^6–80^ was carried out with HADDOCK v2.4 using the starting structures described above. A total of 4000 structures were calculated in the rigid-body energy minimization stage (it0), of which the best 300 were passed on to the semiflexible simulated annealing stage (it1) and thus to the final water refinement stage. The ppant group of APCP was allowed to be flexible (both backbone and side chain) at all stages of the semiflexible simulated annealing stage (it1). HADDOCK was allowed to designate the interface residues (defined as residues with any atom within 5.0 Å of any atom on the other component) as semiflexible regions (both backbone and side chain) during the semiflexible refinement stages of it1. Topology and parameter files for the ppant-substrate moiety generated previously for ARIA/CNS were also used for HADDOCK. The final structures were clustered according to their interface-ligand RMSD (il-RMSD) with a cutoff of 2.0 Å and ranked by their HADDOCK score. The il-RMSD is calculated by first superimposing the structures on the backbone C^α^ atoms of the interface of the largest molecule and then calculating the RMSD of the backbone C^α^ atoms of the interface of the other (smaller) molecules. The standard HADDOCK scoring for protein-protein complexes, which is a weighted sum of the van der Waals intermolecular energy, electrostatic intermolecular energy, desolvation energy, and the distance-restraint violation energy, was used. Forty-five structures with a HADDOCK score of below −100 converged to one conformation. Out of these, five structures were chosen for the next step.

BN^2–77^ was then docked to this dimeric complex, also using HADDOCK, and with settings similar to those given above. Briefly, 5000, 400, and 400 structures were calculated in the it0, it1, and water refinement stages, respectively, and the semiflexible regions in it1 were defined automatically by HADDOCK as described above. The resulting solutions were ranked based on their HADDOCK score and clustered according to il-RMSD with a cutoff of 5.0 Å. Structures were visualized using PyMOL v2.5 (*98*).

## Supplementary Materials

### This PDF file includes:

Figs. S1 to S16 Tables S1 to S5 References
