# Supplementary material for: The specificity of intermodular recognition in a prototypical nonribosomal peptide synthetase depends on an adaptor domain
Source: Sci Adv. 2024 Jun 19;10(25):eadm9404. doi: 10.1126/sciadv.adm9404 (PMC11186497; doi:10.1126/sciadv.adm9404)
Supplement: Supplementary file 1 — Figs. S1 to S16 Tables S1 to S5 References [file sciadv.adm9404_sm.pdf]

Science Advances
AAAS

Supplementary Materials for

The specificity of intermodular recognition in a prototypical nonribosomal peptide synthetase depends on an adaptor domain

Megha N. Karanth *et al.*

Corresponding author: Teresa Carlomagno, t.carlomagno@bham.ac.uk

Sci. Adv. **10**, eadm9404 (2024)
DOI: 10.1126/sciadv.adm9404

This PDF file includes:

Figs. S1 to S16
Tables S1 to S5
References

A

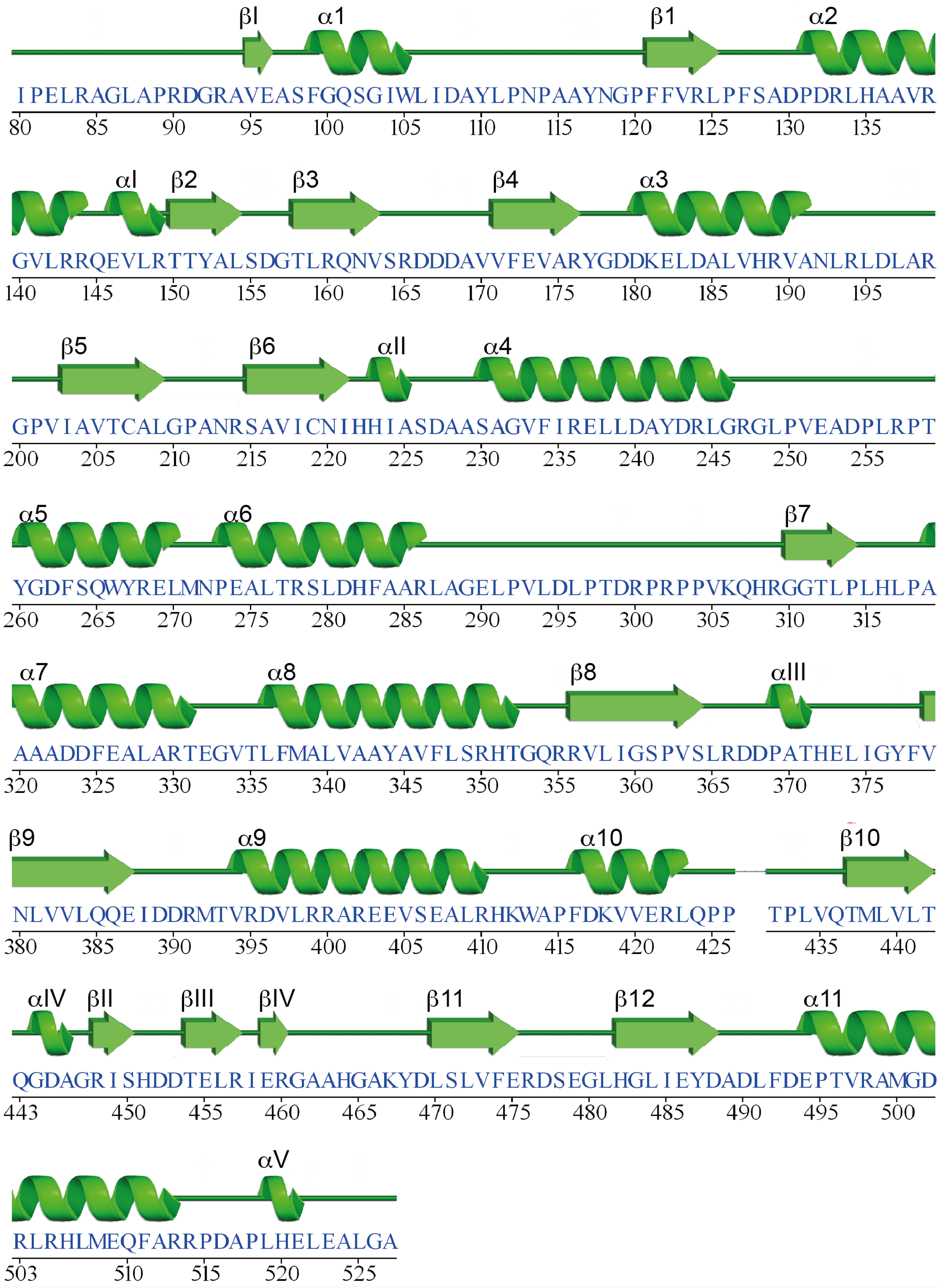
βI α1 β1 α2
IPELRAGLAPRDGRAVEASFGQSGIWLIDAYLPNPAAYNGPFFVRLPFSADPDRLHAAVR
80 85 90 95 100 105 110 115 120 125 130 135
αI β2 β3 β4 α3
GVLRRQEVLRTTYALSDGTLRQNVSRDDDAVVFEVARYGDDKELDALVHRVANLRLDLAR
140 145 150 155 160 165 170 175 180 185 190 195
β5 β6 αII α4
GPVIAVTCALGPANRSAVICNIHHIASDAASAGVFIRELLDAYDRLGRGLPVEADPLRPT
200 205 210 215 220 225 230 235 240 245 250 255
α5 α6 β7
YGDFSQWYRELMNPEALTRSLDHFAARLAGELPVLDLPTDRPRPPVKQHRGGTLPLHLPA
260 265 270 275 280 285 290 295 300 305 310 315
α7 α8 β8 αIII
AAADDFEALARTEGVTLFMALVAAYAVFLSRHTGQRRVLIGSPVSLRDDPATHELIGYFV
320 325 330 335 340 345 350 355 360 365 370 375
β9 α9 α10 β10
NLVVLQQEIDDRMTVRDVLRRAREEVSEALRHKWAPFDKVVERLQPP TPLVQTMLVLT
380 385 390 395 400 405 410 415 420 425 435 440
αIV βII βIII βIV β11 β12 α11
QGDAGRISHDDTELRIERGAAHGAKYDLSLVFERDSEGLHGLIEYDADLFDEPTVRAMGD
443 450 455 460 465 470 475 480 485 490 495 500
αV
RLRHLMEQFARRPDAPLHELEALGA
503 510 515 520 525

B

```
                          1        10        20        30        40        50        60
TomB-C                    MNSPLRTTVLDLARTTLGSADLTAHEPLADRCEHPALLDDLATTLTAVFAVEITGADLAA
PPSB_BACSU|1052-1487      ............................................................
PPSA_BACSU|1048-1483      ............................................................
SRFAA_BACSU|1058-1491     ............................................................
ACVS_EMENI|2018-2466      ............................................................
ACVS_EMENI|944-1398       ............................................................
SRFAB_BACSU|6-451         ............................................................
SRFAC_BACSU|9-451         ............................................................
PPSC_BACSU|7-450          ............................................................
PPSB_BACSU|7-456          ............................................................
ENTF_ECOLI|1-442          ............................................................
ACVS_EMENI|3105-3539      ............................................................
SRFAA_BACSU|3121-3576     ............................................................
SRFAB_BACSU|3117-3567     ............................................................
PPSB_BACSU|2088-2546      ............................................................
PPSA_BACSU|2089-2547      ............................................................
PPSA_BACSU|2-444          ............................................................
SRFAA_BACSU|2-447         ............................................................
PKSJ_BACSU|690-1141       ............................................................
SRFAA_BACSU|2097-2531     ............................................................
SRFAB_BACSU|2093-2527     ............................................................
SRFAB_BACSU|1054-1485     ............................................................

                                   70        80        90        100       110
TomB-C                    GATVADVAARMDDRRDAPRIPELRAGLAPRDGRAVEASFGQSGIWLIDQYLPN.PAAYNG
PPSB_BACSU|1052-1487      ..............................QRDVYPLSFSQKRLYALHQLADD.STGYNM
PPSA_BACSU|1048-1483      ..............................KQETYPVSSAQKRIYVLQQLEDG.GTGYNM
SRFAA_BACSU|1058-1491     ..............................KQEHYPLSLAQQRTYIVSQFEDA.GVGYNM
ACVS_EMENI|2018-2466      ..............................NRRKIAVSPAQERLLFIHELEGG.GNAYNI
ACVS_EMENI|944-1398       ..............................NANVYLANSLQQGFVYQFLKNMGRSEAYVM
SRFAB_BACSU|6-451         ..............................IQKVYALTPMQEGMLYHAMLDPH.SSSYFT
SRFAC_BACSU|9-451         ..............................VQDMYYLSPMQEGMLFHAILNPG.QSFYLE
PPSC_BACSU|7-450          ..............................IQDIYPLSFMQEGMLFHSLYDEQ.SRAYFE
PPSB_BACSU|7-456          ..............................IQDIYPLSHMQEGMLFHSLMDFS.SKAYIE
ENTF_ECOLI|1-442          ..............................MSQHLPLVAAQPGIWMAEKLSEL.PSAWSV
ACVS_EMENI|3105-3539      ..............................VLGDAPLLPIQT..WFLSKSLQH.PSHWNH
SRFAA_BACSU|3121-3576     ..............................VEGEVILTPIQR..WFFEKNFTN.KHHWNQ
SRFAB_BACSU|3117-3567     ..............................VEGEVILTPIQR..WFFEKNFTN.KHHWNQ
PPSB_BACSU|2088-2546      ..............................VEGEITWTPIQQ..WFFSQSL.E.SHHFNQ
PPSA_BACSU|2089-2547      ..............................AEGEAELTPIQR..RFFGQVHAF.HYHYNQ
PPSA_BACSU|2-444          ..............................SEHTYSLTHAQRRVWFTELLEPD.TSICNL
SRFAA_BACSU|2-447         ..............................EITFYPLTDAQKRIWYTEKFYPH.TSISNL
PKSJ_BACSU|690-1141       ..............................QATFQPLSEVQKGLWTLQKMSPE.KSAYHV
SRFAA_BACSU|2097-2531     ..............................KQDTYPVSSPQKRMYVLQQLEDA.QTSYNM
SRFAB_BACSU|2093-2527     ..............................KQDMYPLSSAQKRMYVLNQLDRQ.TISYNM
SRFAB_BACSU|1054-1485     ..............................YQQHYPVSPAQRRMYILNQLGQA.NTSYNV

                          120       130       140       150        160       170
TomB-C                    PFFVRLPFS..ADPDRLHAAVRGVLRRQEVLRTTYALS...DGTLRQNVSRD..DDAVVF
PPSB_BACSU|1052-1487      PAVLELRGN..LNRQRLRSVLTELVNRHEALRTVFVLD...RDEPVQIIYPE..MAFD.L
PPSA_BACSU|1048-1483      PAVLELEGK..LNPERMERAFKELIKRHESLRTSFEQD..AGGDPVQRIHDE..VPFT.L
SRFAA_BACSU|1058-1491     PAAAILEGP..LDIQKLERAFQGLIRRHESLRTSFVLE...NSTPRQKIHDS..VDFN.I
ACVS_EMENI|2018-2466      DAAFELPPY..IDQSRVEEALYTILSRHEALRTFLLRDQ.ATGTFYQKILTT..DEAKCM
ACVS_EMENI|944-1398       QSVLRYDVN..INPDLFKKAWKQVQHMLPTLRLRFQWGQ....DVLQVIDED..QPLN.W
SRFAB_BACSU|6-451         QLELGIHGA..FDLEIFEKSVNELIRSYDILRTVFVHQ..QLQKPRQVVLAE..RKTK.V
SRFAC_BACSU|9-451         QITMKVKGS..LNIKCLEESMNVIMDRYDVFRTVFIHE..KVKRPVQVVLKK..RQFH.I
PPSC_BACSU|7-450          QASFTIHGQ..LDLERFQKSMDAVFDRYDIFRTAFIYK..NVAKPRQVVLKQ..RHCP.I
PPSB_BACSU|7-456          QTSFTITGN..LCVDSFQKSLNLLVSRYDIFRTIFIKEVPDLTGPQQVVLSN..RELT.V
ENTF_ECOLI|1-442          AHYVELTGE..VDSPLLARAVVAGLAQADTLRMRFTED...NGEVWQWVDDA..LTFELP
ACVS_EMENI|3105-3539      TFYLRT.PE..LDVTTLSTAVAELQLYHDAFRMRLR.Q..IDGRTVQCFAD.DISPVQ.L
SRFAA_BACSU|3121-3576     SVMLHAKKG..FDPERVEKTLQALIEHHDALRMVYR.E..GQEDVIQYNRGLEAASAQ.L
SRFAB_BACSU|3117-3567     SVMLHAKKG..FDPERVEKTLQALIEHHDALRMVYR.E..ENGDIVQVYKPIGESKVS.F
PPSB_BACSU|2088-2546      SVMIYRAER..FDEAALRKVLKSLVTHHDALRIVCR.H..EDGRQVQINRGIDLSDEE.L
PPSA_BACSU|2089-2547      SVMLFSEKG..FNANALHLALRKITEHHDAIRMIFQRD..QNGHVIQFNRGINHKDHE.L
PPSA_BACSU|2-444          TACVKFKGN..IELDTLEGALNHSISRNDAIRFQLLEG..EELEPRLHLTEY..KYYP.L
SRFAA_BACSU|2-447         AGIGKLVSADAIDYVLVEQAIQEFIRRNDAMRLRLRLD..ENGEPVQYISEY..RPVD.I
PKSJ_BACSU|690-1141       PLCFKFSSG..LHHETFQQAFGLVLNQHPILKHVIQEK...DGVPFLKNEPA..LSIE.I
SRFAA_BACSU|2097-2531     PAVLRLTGE..LDVERLNSVMQQLMQRHEALRTTFEIK...DGETVQRIWEE..AECE.I
SRFAB_BACSU|2093-2527     PSVLLMEGE..LDISRLRDSLNQLVNRHESLRTSFMEA...NGEPVQRIIEK..AEVD.L
SRFAB_BACSU|1054-1485     PAVLLLEGE..VDKDRLENAIQQLINRHEILRTSFDMI...DGEVVQTVHKN..ISFQ.L
```

```
                                      180       190        200       210
TomB-C                EVARYG...........DDKELDALVHRVANLRLDLA.RGPVIAVTCALGPA.NRSAVIC
PPSB_BACSU|1052-1487  KELEME...........SEQMLESAIE.TFIKPFYLS.SGPLFRACVITMGN.NRGFLLL
PPSA_BACSU|1048-1483  QTTVLG...........E.RTEQEAAA.AFIKPFDLS.QAPLFRAQIVKISD.ERHLLLV
SRFAA_BACSU|1058-1491 EMIERG...........G.RSDEAIMA.SFVRTFDLA.KAPLFRIGLLGLEE.NRHMLLF
ACVS_EMENI|2018-2466  LIIEKS.A..V.....STIDQIDSIVGRLSQHIFRLD.SELPWLAHIVTHKT.GNLYLTL
ACVS_EMENI|944-1398   WFLHLADDSAL.....PEEQKLLELQRRDLAEPYDLA.AGSLFRIYLIEHSS.TRFSCLF
SRFAB_BACSU|6-451     HYEDISHADEN.....RQKEHIERYKQDVQRQGFNLA.KDILFKVAVFRLAA.DQLYLVW
SRFAC_BACSU|9-451     EEIDLTHLTGS.....EQTAKINEYKEQDKIRGFDLT.RDIPMRAAIFKKAE.ESFEWVW
PPSC_BACSU|7-450      HIEDISHLNER.....DKEHCTEAFKEQDKSKGFDLQ.TDVLMRISILKWAP.DHYVCIW
PPSB_BACSU|7-456      YREDISRLADQ.....EQQTLIDAFMTKDREKGFDLQ.KDPLMRLALFDRGD.SQYTCVW
ENTF_ECOLI|1-442      EIIDLRTN..I.....DPHGTAQALMQADLQQDLRVDSGKPLVFHQLIQVAD.NRWYWYQ
ACVS_EMENI|3105-3539  RVLNVKDVDG........SAAIDQQLQ.KYQSDFDLE.KGPICAAAYLHGYEDRSARVWF
SRFAA_BACSU|3121-3576 EVIQIEGQAA...D...YEDRIEREAE.RLQSSIDLQ.EGGLLKAGLFQAE..DGDHLLL
SRFAB_BACSU|3117-3567 EIVDLYGSDE...EM..LRSQIKLLAN.KLQSSLDLR.NGPLLKAEQYRTE..AGDHLLI
PPSB_BACSU|2088-2546  YALELFDVKD...SLTEARNTIEEAAS.RMQEHIRLE.TGPLLHAGLFRTE..NGDHLFL
PPSA_BACSU|2089-2547  FGLYISDWTKASLERAHLDEKLAAEET.VIQSKMNVE.KGPLLQAGLFKTA..EGDHLLI
PPSA_BACSU|2-444      RIIDFS....N.....VEMIEIEQWIQDQASIPFKLF.NSPLFQFYLLRIDS.HEVWLFA
SRFAA_BACSU|2-447     KHTDTTED..P.....NAIEFISQWSREETKKPLPLY.DCDLFRFSLFTIKE.NEVWFYA
PKSJ_BACSU|690-1141   KTENIS.S..L.....KE.SDIPAFLRKKVKEPYVKE.NSPLVRVMSFSRSE.QEHFLLV
SRFAA_BACSU|2097-2531 AYFEAP...........E.EETERIVS.EFIKPFKID.QLPLFRIGLIKHSD.TEHVLLF
SRFAB_BACSU|2093-2527 HVFEAK...........E.DEADQKIK.EFIRPFDLN.DAPLIRAALLRIEA.KKHLLLL
SRFAB_BACSU|1054-1485 EAAKGR...........E.EDAEEIIK.AFVQPFELN.RAPLVRSKLVQLEE.KRHLLLI

                   220       230       240        250        260       270
TomB-C                NIHHIASDAASAGVFLRELLDAYDRLGR.GLPVEADPLRPTYGDFSQWYRELMNPEALTR
PPSB_BACSU|1052-1487  DMHHIIADGVSMSTLVQEFTDLYCGK.......ELPALNLHYKDFAVWQQEKHPKELYKK
PPSA_BACSU|1048-1483  DMHHIISDGVSVNILIREFGELYNNR.......NLPALRIQYKDYAVWREGFKTGDAYKT
SRFAA_BACSU|1058-1491 DMHHLISDGVSIGIMLEELARIYKGE.......QLPDLRLQYKDYAVWQSRQ.AAEGYKK
ACVS_EMENI|2018-2466  SFHHTCFDAWSLKIFERELRVFCASNEKGGNMPILPMPQVQYKEYAEHHRRRLGKNQIQK
ACVS_EMENI|944-1398   SCHHAILDGWSLPLLFRKTHGTYLHLLHGHSLR...TLEDPYRQSQQYL.QD....HRED
SRFAB_BACSU|6-451     SNHHIMMDGWSMGVLMKSLFQNYEALRAGRTPA..NGQGKPYSDYIKWLGKQ....DNEE
SRFAC_BACSU|9-451     SYHHIILDGWCFGIVVQDLFKVYNALREQKPYS..LPPVKPYKDYIKWLEKQ....DKQA
PPSC_BACSU|7-450      SHHHILMDGWCLGIVIKDFLHIYQALGKGQLPD..LPPVQPYGTYIKWLMQQ....DREE
PPSB_BACSU|7-456      THHHIIMDGWCLGIILKEFFSMYDSLKNNSPVQ..LGSTVPYSRYIEWLGEQ....DQEE
ENTF_ECOLI|1-442      RYHHLLVDGFSFPAITRQIANIYCTWLRGEPTP..ASPFTPFADVVEEYQQYRESEAWQR
ACVS_EMENI|3105-3539  SVHHIIIDIVSWQILARDLQILYEGGT.......LGRKSSSVRQWAEALQSYQGSA...S
SRFAA_BACSU|3121-3576 AIHHLVVDGVSWRILLEDFAAVYTQLEQGNEP.VLPQKTHSFAEYAERLQDFANSKAFLK
SRFAB_BACSU|3117-3567 AVHHLVVDGVSWRILLEDFASGYMQAEKEESL.VFPQKTNSFKDWAEELAAFSQSAHLLQ
PPSB_BACSU|2088-2546  TIHHLVVDAVSWRILFEDFSTAYKQAVSGESI.KLPQKTDSYLTYSQRIADYSISRQVQR
PPSA_BACSU|2089-2547  ALHHLVIDGVSWRILLEDLAAAYQQALEKKEI.QLPPKTDSYLSYADGLTQIAESKQLLS
PPSA_BACSU|2-444      KFHHIIMDGISLNVMGNQIIDLYQKMKKKDPLP..DQPEPSYLSYIEKESQYLQSPRFAK
SRFAA_BACSU|2-447     NVHHVISDGISMNILGNAIMHIYLELASGSETK..EGISHSFIDHVLSEQEYAQSKRFEK
PKSJ_BACSU|690-1141   VIHHLIFDGVSSVTFIRSLFDTYQLLLKGQQP.EKAVSPAIYHDFAAWEKNMLAGKDGVK
SRFAA_BACSU|2097-2531 DMHHIISDGASVGVLIEELSKLYDGE.......TLEPLRIQYKDYAVWQQQFIQSELYKK
SRFAB_BACSU|2093-2527 DMHHIIADGVSRGIFVKELALLYKGE.......QLPEPTLHYKDFAVWQNEAEQKERMKE
SRFAB_BACSU|1054-1485 DMHHIITDGSSTGILIGDLAKIYQGA.......DLELPQIHYKDYAVWHKEQ...TNYQK

                     280       290       300                310        320
TomB-C                SLDHFAARLAGEL.PVLDLPTDRPRP.....PVKQH....RGGTLPLHLP.AAAADDFEA
PPSB_BACSU|1052-1487  QEAYWLGQLGGSL.PTLELPLDKTRP.....RLPDF....RGGTIEVNID.KDMADELHR
PPSA_BACSU|1048-1483  QEAYWLKQLEGEL.PVLDLPADHARP.....PVRSF....AGDKVSFTLD.QEVASGLHK
SRFAA_BACSU|1058-1491 DQAYWKEVFAGEL.PVLQLLSDYPRP.....PVQSF....EGDRVSIKLD.AGVKDRLNR
ACVS_EMENI|2018-2466  LSDFWLQRLDGLE.P.LQLLPDYPRP.....AQFNY....DGGDLSVILD.GVVLETLRG
ACVS_EMENI|944-1398   HLRYWAGIVNQIE.ERCDMNALLNERSRYKIQLADYDKVEDQQQLTLTVPDASWLSKLRQ
SRFAB_BACSU|6-451     AESYWSERLAGFE.QPSVLPGRLPVK......KDEY....VNKEYSFTWD.ETLVARIQQ
SRFAC_BACSU|9-451     SLRYWREYLEGFE.GQTTFAEQRKK......QKDGY....EPKELLFSLS.EAETKAFTE
PPSC_BACSU|7-450      AAEYWKKRLQHFE.KSTPLPKRTDQI.....PNGTL......QQITFAIP.EKETAELQK
PPSB_BACSU|7-456      TAAYWSEYLKEYG.NTASIPRIKRRT.....ADGNY....KADQVSFSLA.PDMVEKLTE
ENTF_ECOLI|1-442      DAAFWAEQRRQLP.PPASLS.PAP.L.....PGRSA....SADILRLKLE.FTDG.EFRQ
ACVS_EMENI|3105-3539  ERAYWEGLLAQTAANISALPPVTGTR.....TR...........LARTWS.DDRTV.ILL
SRFAA_BACSU|3121-3576 EKEYWRQLEEQAVAA..KLPKDRESG.....DQ.RM...KHTKTIEFSLT.AEETEQLTT
SRFAB_BACSU|3117-3567 QAEYWSQIAAEQV.S..PLPKDCETE.....QR.IV...KDTSSVLCELT.AEDTKHLLT
PPSB_BACSU|2088-2546  EAAYWDECENRHI.Q..PIPKDNDAA.....SN.TF...KDTEVIDFELS.RHHTELLLT
PPSA_BACSU|2089-2547  EKTYWQTILDAHT.A..FLPKDIENV.....PD.KL..QMNSDAAAFVLS.GDWTEKLLF
PPSA_BACSU|2-444      DRLFWTQTFEHPL.E.YHSLADQTSL.....QKQST....SASRDTIILS.PDLEQTIRI
SRFAA_BACSU|2-447     DKAFWNKQFESVP.E.LVSLKRNASA.....GG.SL....DAERFSKDVP.EALHQQILS
PKSJ_BACSU|690-1141   HRTYWQKQLSGTL.PNLQLPNVSASS.....VDSQF....REDTYTRRLS.SGFMNQVRT
SRFAA_BACSU|2097-2531 QEEHWLKELDGEL.PVLTLPTDYSRP.....AVQTF....EGDRIAFSLE.AGKADALRR
SRFAB_BACSU|2093-2527 HEAYWMSVLSGEL.PELDLPLDYARP.....PVQSF....KGDTIRFRTG.SETAKAVEK
SRFAB_BACSU|1054-1485 DEEYWLDVFKGEL.PILDLPADFERP.....AERSF....AGERVMFGLD.KQITAQIKS
```

```
                        330       340       350       360          370       380
TomB-C                  L.ARTEGVTLFMALVAAYAVFLSRHTGQRRVLIGSPVSLRDDP.....ATHELIGYFVNL
PPSB_BACSU|1052-1487    L.MAETGTTLYMILLAVYSILLSKLSGQEDIVVGSPAAGRPHA.....DLERVIGMFVNT
PPSA_BACSU|1048-1483    L.ARENGSTLYMVLLAAYTAFLSRLSGQEDIIVGSPIAGRPHK.....DLEPILGMFVNT
SRFAA_BACSU|1058-1491   L.AEQNGATLYMVMLSAYYTLLSKYTGQDDIIVGTPSAGRNHS.....DTEGIIGMFVNT
ACVS_EMENI|2018-2466    I.AKDHGVTLYAVLLAVYCLMLSTYTHQVDIAVGVPISHRTHP.....LFQSIVGFFVNM
ACVS_EMENI|944-1398     T.CSAQGITLHSILQFVWHAVLHAYGGGTHTVTGTTISGRNLPV...SGIERSVGLYINT
SRFAB_BACSU|6-451       T.ANLHQVTGPNLFQAVWGIVLSKYNFTDDVIFGTVVSGRPSEI...NGIETMAGLFINT
SRFAC_BACSU|9-451       L.AKSQHTTLSTALQAVWSVLISRYQQSGDLAFGTVVSGRPAEI...KGVEHMVGLFINV
PPSC_BACSU|7-450        I.AAASGATLNTVFQALWGIMLQKVNRSSDAVFGSVISGRPSEL...KDVENMVGLFINT
PPSB_BACSU|7-456        A.AQNWGVTLNTLFMSIWGVLLHRYNAADDAVFGSVISGRPSAI...DGIESMVGLFINT
ENTF_ECOLI|1-442        LATQLSGVQRTDLALALAALWLGRLCNRMDYAAGFIFMRRLGS.....AALTATGPVLNV
ACVS_EMENI|3105-3539    NEASNQNASIQDLLLAAVGLALQQVTPGSPSMITLEGHGREEIVDPTLDLSRTLGWFTSM
SRFAA_BACSU|3121-3576   KVHEAYHTEMNDILTAFGLAMKEWTGQDRVSVHLEGHGREEII.EDLTISRTVGWFTSM
SRFAB_BACSU|3117-3567   DVHQPYGTEINDILLSALGLTMKEWTKGAKIGINLEGHGREDII.PNVNISRTVGWFTAQ
PPSB_BACSU|2088-2546    AAHKAYSTEMNDILLTALGLALQKWTGNNQFKISMEGHGRESYL.EDIDISRTVGWFTSI
PPSA_BACSU|2089-2547    ETQQAYGTDANELLLTALGMALSEWTGHDQIVISTEGHGREGHV.PNIDISRTVGWFTSI
PPSA_BACSU|2-444        F.CEEHKINIISLFMASFYICISRITSKKDLAIGTYYGNRGSK.....AEKEMLGMFVSS
SRFAA_BACSU|2-447       F.CEANKVSVLSVFQSLLAAYLYRVSGQNDVVTGTFMGNRTNA.....KEKQMLGMFVST
PKSJ_BACSU|690-1141     F.AKEHSVNVTTVFLSCYMMLLGRYTGQKEQIVGMPAMVRPEE.....RFDDAIGHFLNM
SRFAA_BACSU|2097-2531   L.AKETDSTLYMVLLASYSAFLSKISGQDDIIVGSPVAGRSQA.....DVSRVIGMFVNT
SRFAB_BACSU|2093-2527   L.LAETGTTLHMVLHAVFHVFLSKISGQRDIVIGSVTAGRTNA.....DVQDMPGMFVNT
SRFAB_BACSU|1054-1485   L.MAETDTTMYMFLLAAFNVLLSKYASQDDIIVGSPTAGRTHP.....DLQGVPGMFVNT
```

```
                            390       400       410          420       430
TomB-C                  VVLQQEIDD..RMTVRDVLRRAREEVSEALRHK...WAPFDKVVE..RLQPPRSSGYTPL
PPSB_BACSU|1052-1487    LAMRSKPEG..HKTFSSYLHDIRHLALTAYEHQ...DYPFEELAD..KLDTNREVNRNPL
PPSA_BACSU|1048-1483    LALRTRPEG..GKPFVQYLQEVRETALEAFEHQ...DYPFEELVD..KLELTRDMSRNPV
SRFAA_BACSU|1058-1491   LAIRSEVKQ..NETFTQLISRVRKRVLDAFSHQ...DYPFEWLVE..DLNIPRDVSRHPL
ACVS_EMENI|2018-2466    VVVRVDVKD...FAVHDLIRRVMKALVDAQLHQ...DMPFQDVTK..LLRVDNDASRHPL
ACVS_EMENI|944-1398     LPLVINQLAYKNKTVLEAIRDVQAIVNGMNSRG...NVELGRLQK...N.....ELKHGL
SRFAB_BACSU|6-451       IPVRVKVER..DAAFADIFTAVQQHAVEAERYD...YVPLYEIQK..RS.....ALDGNL
SRFAC_BACSU|9-451       VPRRVKLSE..GITFNGLLKRLQEQSLQSEPHQ...YVPLYDIQS..QA.....D.QPKL
PPSC_BACSU|7-450        IPIRAQS.D..SLSFSDLVRRMQKDMNEAEAYS...YFPLYDIQA..QS.....ALKQEL
PPSB_BACSU|7-456        VPVRIRSAE..GITFSSLVKAVQEDILSSEQHG...YYPLYEIQN..HS.....PLKQGL
ENTF_ECOLI|1-442        LPLGIHIAA..QETLPELATRLAAQLKKMRRHQ...RYDAEQIVR..DS..GRAAGDEPL
ACVS_EMENI|3105-3539    YPFEIPPLNVE......TLSQGIASLRECLRQVPARGIGFGSLYGYC.........KHQM
SRFAA_BACSU|3121-3576   YPMVLDMKHAD......DLGYQLKQMKEDIRHVPNKGVGYGILRYLTAPEHKEDVAFSIQ
SRFAB_BACSU|3117-3567   YPVVLDISDAD......A.SAVIKTVKENLRRIPDKGVGYGILRYFTETAET....KGFT
PPSB_BACSU|2088-2546    YPVWLDMRDSDHKDKEERLGHLIKQTKDMLHRIPHKGAGYGVLKYISKRWGS....QKNS
PPSA_BACSU|2089-2547    YPILLDMGIPEP..FEDQLAYRIKTTKDMLRRVPNKGTGYGLLTHIGELRHK.......E
PPSA_BACSU|2-444        LPIRITVDP..DTDFLSFVRTIGREQLSVMRHQ...RFPYNLLVN..ELRNEQKD.LHNL
SRFAA_BACSU|2-447       VPLRTNIDG..GQAFSEFVKDRMKDLMKTLRHQ...KYPYNLLIN..DLRETKSS.LTKL
PKSJ_BACSU|690-1141     LPIRSELNP..ADTFSSFISKLQLTILDGLDHA...AYPFPKMVR..DLNIPRSQAGSPV
SRFAA_BACSU|2097-2531   LALRTYPKG..EKTFADYLNEVKETALSAFDAQ...DYPLEDLIG..NVQVQRDTSRNPL
SRFAB_BACSU|2093-2527   LALRMEAKE..QQTFAELLELAKQTNLSALEHQ...EYPFEDLVN..QLDLPRDMSRNPL
SRFAB_BACSU|1054-1485   VALRTAPAG..DKTFAQFLEEVKTASLQAFEHQ...SYPLEELIE..KLPLTRDTSRSPL
```

```
                          440       450                    460       470
TomB-C                  VQTMLVLT.....QGDAGRISHD.........DTELRI...ERGAAHGAKYDL..SLVFE
PPSB_BACSU|1052-1487    FDAMLVLQ.....SSEDFRFEVP.........GLSISS...VTPKHDISKFDL..TLHAE
PPSA_BACSU|1048-1483    FDAMFILQ.....NVEKQDIDLR.........EIKVRP...ANFAHHISLFDI..TLIAT
SRFAA_BACSU|1058-1491   FDTMFSLQ.....NATEGIPAVG.........DLSLSV...QETNFKIAKFDL..TVQAR
ACVS_EMENI|2018-2466    VQTVFNFE.....SDMDKEFETT..PSIQD..TATIAPYQSVQRIKSVAKFDL..NATAT
ACVS_EMENI|944-1398     FDSLFVLENYPILDKS....EEM...RQKSELKY..T....IEGNIEKLDYPLAVIAREV
SRFAB_BACSU|6-451       LNHLVAFENYPLDQEL....ENG...SMEDRLGFSIK....VESAFEQTSFDFNLIVY..
SRFAC_BACSU|9-451       IDHIIVFENYPLQDAK....NEE...SSEN..GFDMV....DVHVFEKSNYDLNLMAS..
PPSC_BACSU|7-450        IDHIIVFENTPTQQEI....EEL...NQAGSFDFSVK....DFEMEEVTNYSCSVKVI..
PPSB_BACSU|7-456        IDHIFVFENYPVQLHQALSVESE...NDEG..ALKLS....DISMSEQTNYDFNIVIV..
ENTF_ECOLI|1-442        FGPVLNIKVFDYQLDIPD.VQAQ..................THTLATGPVNDLELAL.FP
ACVS_EMENI|3105-3539    PQVTFNYLGQLTSKQSITDQWALAVGDGEMQYGLTTSP.....ADRDQSSFAV..DITAS
SRFAA_BACSU|3121-3576   PDVSFNYLGQFDEMSDAGLFT...R..SELPSGQSLS.......PETEKPNAL..DVVGY
SRFAB_BACSU|3117-3567   PEISFNYLGQFDSEVKTDFFE...P..SAFDMGRQVS.......GESEALYAL..SFSGM
PPSB_BACSU|2088-2546    PEISFNYLGQFDQDIQSNAFE...V..SDIKPGNEIS.......PNWERPYAL..DISGA
PPSA_BACSU|2089-2547    PEVSFNYLGQFSEEKEVETFQ...L..SYYQPRYEIA.......GEREREYEL..DINAL
PPSA_BACSU|2-444        IGISMQYQPLQWHNADDFDYETA...............L.....YFSGYTANELSVQIQER
SRFAA_BACSU|2-447       FTVSLEYQVMQWQKEEDLAFLTE...............P.....IFSGSGLNDVSIHVKDR
PKSJ_BACSU|690-1141     FQTAFFYQ.....NFLQSGSYQSLLSRYAD..FFSVD...FVEYIHQEGEYEL..VFELW
SRFAA_BACSU|2097-2531   FDAVFSMQ.....NANIKDLTMK.........GIQLEP...HPFERKTAKFDL..TLTAD
SRFAB_BACSU|2093-2527   FNVMVTTE.....NPDKEQLTLQ.........NLSISP...YEAHQGTSKFDL..TLGGF
SRFAB_BACSU|1054-1485   FSVMFNMQ.....NMEIPSLRLG.........DLKISS...YSMLHHVAKFDL..SLEAV
```

```
                        480       490       500       510         520       530
TomB-C                  RDSEGLHGLIEYDADLFDEPTVRAMGDRLRHLMEQFARR....PDAPLHELEALGAQERR
PPSB_BACSU|1052-1487    EHLSGIRCRFEYSTALFEEETITQWASYFIELVKGVTAD....TEMRISNMQLLPAAERR
PPSA_BACSU|1048-1483    EISGSICCEMEFSTEVFLKATIERWADHFIEFLHEALST....PETSLAQINILSDKEKQ
SRFAA_BACSU|1058-1491   ETDEGIEIDVDYSTKLFKQSTADRLLTHFARLLEDAAAD....PEKPISEYKLLSEEEAA
ACVS_EMENI|2018-2466    ESGSALKINFNYATSLFRKETIQGFLETYRHLLLQLSYL....GSQGLK........EDT
ACVS_EMENI|944-1398     DLTGGFTFTICYARELFDEIVISELLQMVRDTLLQVAKH....LDDPVRSLEYLSSAQMA
SRFAB_BACSU|6-451       .PGKTWTVKIKYNGAAFDSAFIERTAEHLTRMMEAAVDQ....PAAFVREYGLVGDEEQR
SRFAC_BACSU|9-451       .PGDEMLIKLAYNENVFDEAFILRLKSQLLTAIQQLIQN....PDQPVSTINLVDDRERE
PPSC_BACSU|7-450        .PGRTLYVRIHFQTSAYQPSMMSEIKDYLLHMVSDVISD....PSLPVSKMTLLDEDKTR
PPSB_BACSU|7-456        .PGESFYIKFSYNADVYEREEMLRIQGHLKQALDCILTN....PDVAVSDINIVPPEEQQ
ENTF_ECOLI|1-442        DVHGDLSIEILANKQRYDEPTLIQHAERLKMLIAQFAAD....PALLCGDVDIMLPGEYA
ACVS_EMENI|3105-3539    CVNGALSVEMNS...AWSLEKSMRFISRIEEVLNMILSGTLAQQATPVLTPQVFNEEMY.
SRFAA_BACSU|3121-3576   IENGKLTMSLAYHSLEFHEKTVQTFSDSFKAHLLRIIEHCLSQDGT.ELTPSDLGDDDLT
SRFAB_BACSU|3117-3567   IRNGRFVLSCSYNEKEFERATVEEQMERFKENLLMLIRHCTEKEDK.EFTPSDFSAEDLE
PPSB_BACSU|2088-2546    VSSGCLNMHIIYNRFQFEEKTIQTFSRHFKQTLENIIEHCTGKENQ.EWSASDFTDEDLT
PPSA_BACSU|2089-2547    ITDGRLHVKAVYTQ.VFSKHSIECFMDRFHRHLIETIEHCSQKKAR.EKTLSDFSNKELT
PPSA_BACSU|2-444        IDNGTIQLNFDYQNTLFSLEDIKRIQSHLLTILENALKH....PHSFIRELDMTNTREKQ
SRFAA_BACSU|2-447       WDTGKLTIDFDYRTDLFSREEINMICERMITMLENALTH....PEHTIDELTLISDAEKE
PKSJ_BACSU|690-1141     ETEEKMELNIKYNTGLFDAASISAMFDHFVYVTEQAMLN....PSQPLKEYSLLPEAEKQ
SRFAA_BACSU|2097-2531   ETDGGLTFVLEYNTALFKQETIERWKQYWMELLDAVTGN....PNQPLSSLSLVTETEKQ
SRFAB_BACSU|2093-2527   TDENGIGLQLEYATDLFAKETAEKWSEYVLRLLKAVADN....PNQPLSSLLLVTETEKQ
SRFAB_BACSU|1054-1485   EREEDIGLSFDYATALFKDETIRRWSRHFVNIIKAAAN....PNVRLSDVDLLSSAETA
```

```
TomB-C                  SVL.
PPSB_BACSU|1052-1487    LLL.
PPSA_BACSU|1048-1483    KIV.
SRFAA_BACSU|1058-1491   SQI.
ACVS_EMENI|2018-2466    KLLL
ACVS_EMENI|944-1398     QL..
SRFAB_BACSU|6-451       QIV.
SRFAC_BACSU|9-451       FLL.
PPSC_BACSU|7-450        KIV.
PPSB_BACSU|7-456        VI..
ENTF_ECOLI|1-442        QL..
ACVS_EMENI|3105-3539    ....
SRFAA_BACSU|3121-3576   ....
SRFAB_BACSU|3117-3567   ....
PPSB_BACSU|2088-2546    ....
PPSA_BACSU|2089-2547    ....
PPSA_BACSU|2-444        KLL.
SRFAA_BACSU|2-447       KLL.
PKSJ_BACSU|690-1141     MIL.
SRFAA_BACSU|2097-2531   ALL.
SRFAB_BACSU|2093-2527   ALL.
SRFAB_BACSU|1054-1485   ALL.
```

C

```
                    α1                        α2
APCP        ℓℓℓℓℓℓℓℓℓℓℓℓℓℓℓℓ      TT       ℓℓℓℓℓℓℓℓℓℓℓℓℓℓℓ
            1         10        20        30        40        50
APCP   .....TAVQNPLETVVLQAWKDISGADDFTTTDSFLGHGGNSLHFVQLASRLQKIFGVEV
BPCP   PAPPGRPPRPGLEQRIAREWADVLKVPAVGRDEDFFSVGGNSIIATELVYRLRRAFDQDL
```

```
          α3          α4          η1
APCP   ℓℓℓℓℓℓℓℓ  ℓℓℓℓℓℓℓℓℓℓℓℓℓℓℓℓℓ
          60        70        80
APCP   STEDVFRHGTVEQLARFVEQSRDTGRNPAAQTQ
BPCP   SLRALLENPTVAGMAARLRS......GPGAP..
```

Fig. S1. Secondary structure and sequence alignment of C and PCP domains. (**A**) Secondary structure of BC. Helices are labelled as α followed by either an Arabic or a Roman number if either longer than or equal to one turn, respectively. β-strands are labelled as β followed by an Arabic number if equal to or longer than 5 residues. Shorter β-strands are labelled as β followed by a Roman number. The figure was prepared with PDBsum1 (*99*). (**B**) Alignment of TomB$^{1-533}$ with the seed sequences of the condensation domain family in PFAM (*46*). PPSA/B/C_BACSU is the Plipastatin synthetase subunit A/B/C from *B. subtilis*. SRFAA/B_BACSU is the Surfactin synthetase subunit 1/2 from *B. subtilis.* ACVS_EMENI is the N-(5-amino-5-carboxypentanoyl)-L-cysteinyl-D-valine synthetase from *Emericella nidulans*. ENTF_ECOLI is the Enterobactin synthetase component F from *E.coli*. PKSJ_BACSU is the Polyketide synthase PksJ from *B. subtilis*. (**C**) Secondary structure and alignment of APCP and BPCP.

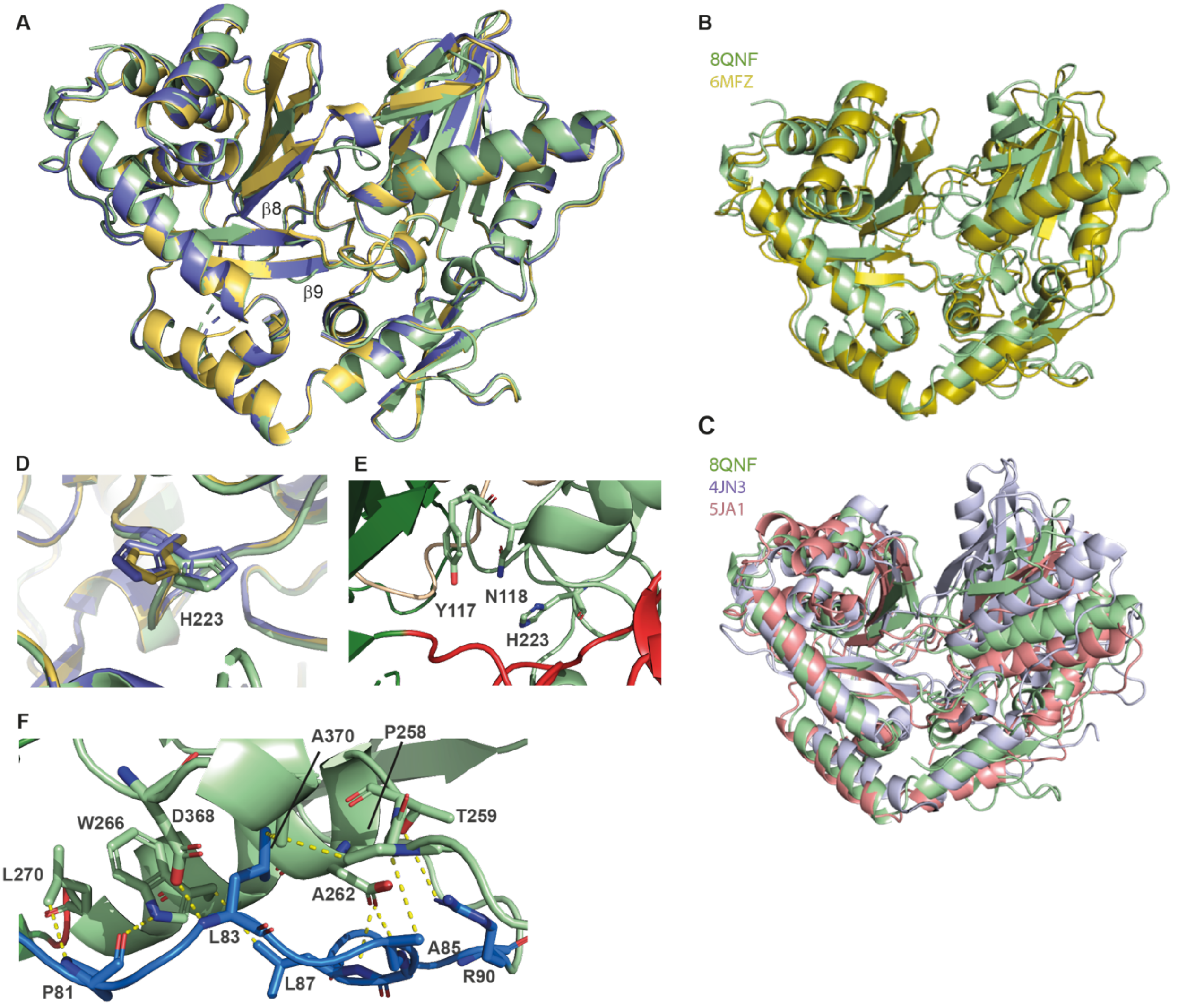

Fig. S2. The crystal structures of BN-BC.

(A) Overlay of the three crystallographic structures of BN-BC determined in this study. Light green: structure of wild-type BN-BC (N-form). Yellow and blue: structures of the two chains in the asymmetric unit cell of Se-Met BN-BC (S-form). Strands β8 and β9 are each three amino acids longer in the S-form than in the N-form. There is no resolvable electron density for the first 79 amino acids. **(B)** Overlay of the crystallographic structure of BN-BC in the N-form (PDB ID 8QNF, green) with that of the condensation domain of LgrA (PDB ID 6MFZ, gold). **(C)** Overlay of the crystallographic structure of BN-BC in the N-form (PDB ID 8QNF, green) with those of the condensation domain of the CDA synthetase (PDB ID 4JN3, violet) and the condensation domain of the Enterobactin synthaetse component F (PDB ID 5JA1, pink). The structures have been superposed on the C-lobe to highlight the differences in the inter-lobe orientations.(**D**) Close-up view of the catalytic histidine H223 in the S-form, with an empty active-site tunnel, where H223-$N^{\varepsilon 2}$ faces outwards, away from the tunnel and towards helix α1, and in the N-form, with a glycerol molecule in the tunnel (not shown), where H223-$N^{\varepsilon 2}$ faces inwards. (**E**) The sidechains of residues

Y117 and N118 in the α1–β1 loop protruding into the active site with the floor loop coloured in red. **(F)** Contacts of residues 80–90 of TomB (in blue) with the BC domain. Distances of less than 4 Å are indicated by dashed yellow lines. Hydrophobic contacts include the sidechains of P81–L270, L83–A370, L83–P258, L87–W266, A85–P258; hydrogen bonds include P81-C–W266-$N^{\varepsilon 1}$, L83-N–D368-$O^{\delta 2}$, L87-N–D262-$O^{\delta 1}$, G86-N–D262-$O^{\delta 1}$, R90-$N^{\eta 1}$–T259-$O^{\gamma 1}$.

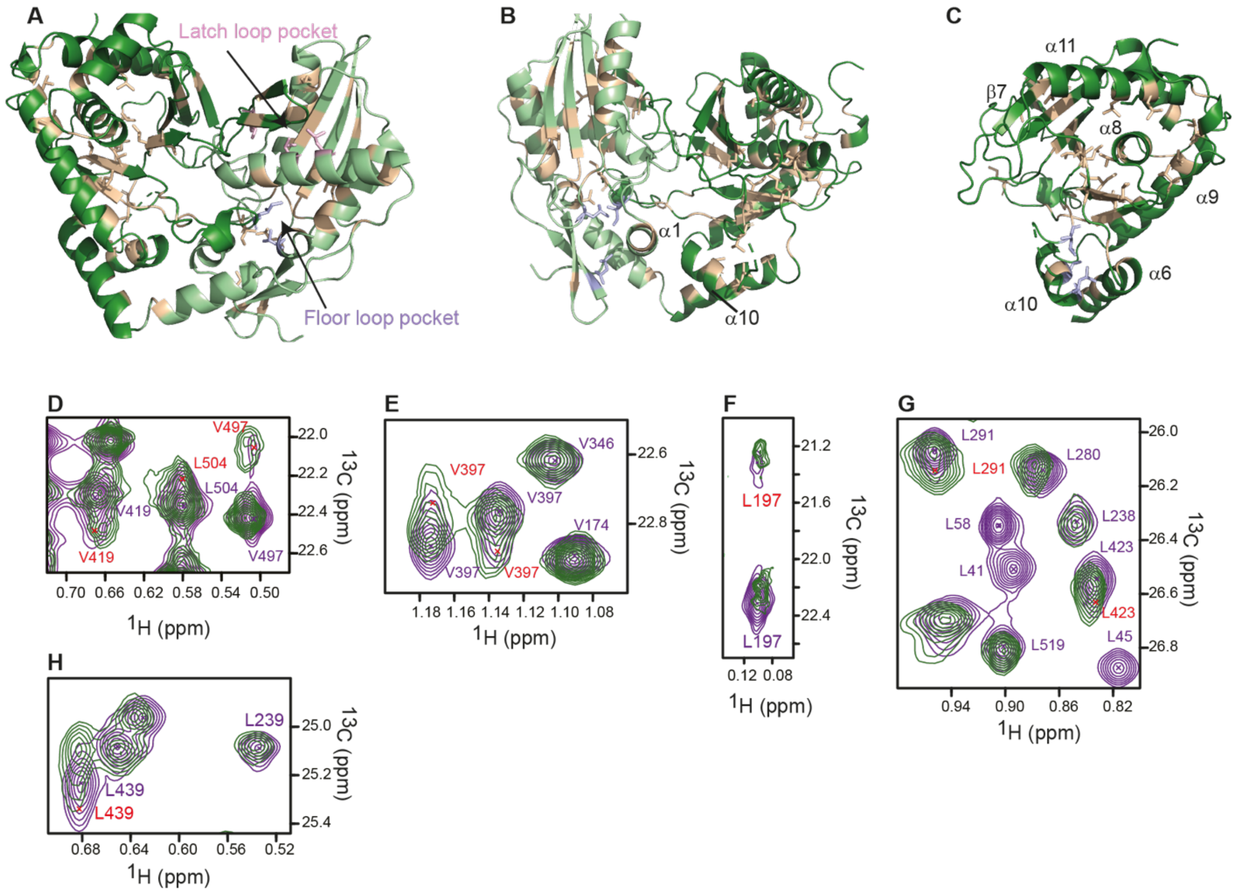

Fig. S3. Conformational heterogeneity of the BC domain. Three views of the crystallographic structure (N-form) of BN-BC (in green) with the ILV residues in wheat and the particular ILV residues displaying multiple conformations in NMR spectra of BN-BC shown additionally in stick representation. (**A**) Two clusters of ILV residues showing multiple conformations are localized within the two pockets in the N-lobe (light green) that accommodate the latch loop (light pink pocket) and the floor loop (light violet pocket). (**B**) A third cluster of ILV residues showing multiple conformations is found in the pocket accommodating helix α1 in the N-lobe (light violet pocket). (**C**) A few ILV residues in the C-lobe (dark green) show conformational heterogeneity and form a large cluster around the central helix α8 and a smaller cluster (light violet) involving helix α10 and its interface with helix α6. (**D–H**) Overlay of excerpts of the ^{1}H,^{13}C-methyl-HMQC spectra of BN-BC (purple) and BC (green) showing methyl groups that adopt two conformations. Purple and red labels indicate the major and minor conformations, respectively. Excerpts in (D) and (F) correspond to those in Fig. 3C and 3D, respectively.

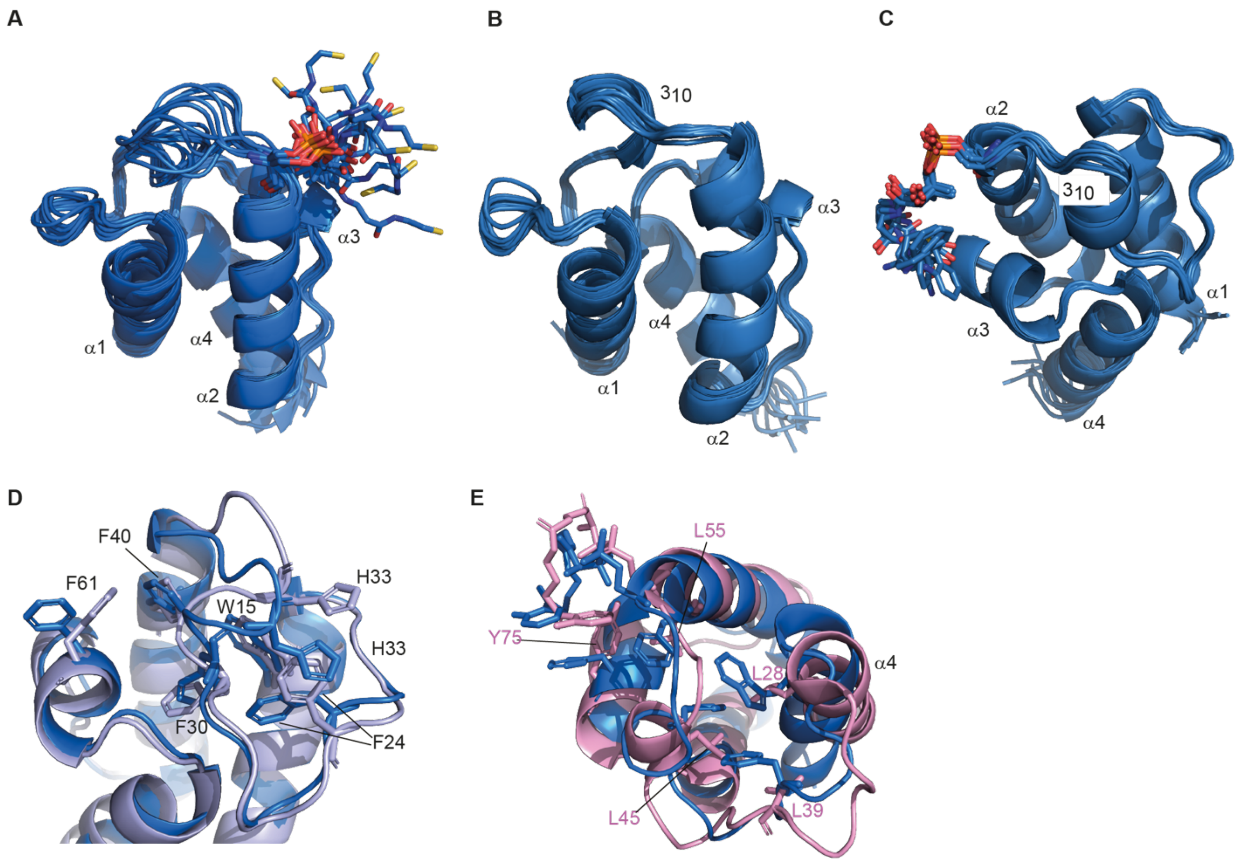

Fig. S4. The NMR structures of $APCP_{ppant}$ and $APCP_{load}$. (**A**) Overlay of the 10 lowest-energy structures of $APCP_{ppant}$ (aa 5–81). The ppant arm is shown in stick representation and is largely disordered. (**B**) Overlay of the 10 lowest-energy structures of $APCP_{load}$ (aa 5–81, ppant arm not shown). The loop α1–α2 adopts a well-defined conformation and forms a short 3_{10} helix. (**C**) Same as in (B) but showing the ppant arm in stick representation. The ppant arm curls back on itself and the substrate nestles on the top of helix α3. **(D)** Overlay of the representative structures of $APCP_{ppant}$ (light blue) and $APCP_{load}$ (marine blue) (same structures as in Fig. 2), showing the differences in the orientation of F61 and of the residues forming the aromatic cluster. **(E)** Overlay of the structures of $APCP_{load}$ (marine blue) and $ArCP_{load}$ (PDB ID 2N6Z, pink). The residues of ArCP corresponding to those forming the aromatic cluster in APCP are shown in sticks and are annotated. The ppant arm, the substrate and the aromatic residue interacting with the substrate (APCP-F61 and ArCP-Y75) are also shown in sticks in both structures. In ArCP, α1 and α4 are respectively longer and shorter in ArCP than in APCP, which leads to a slightly different packing of these helices against each other.

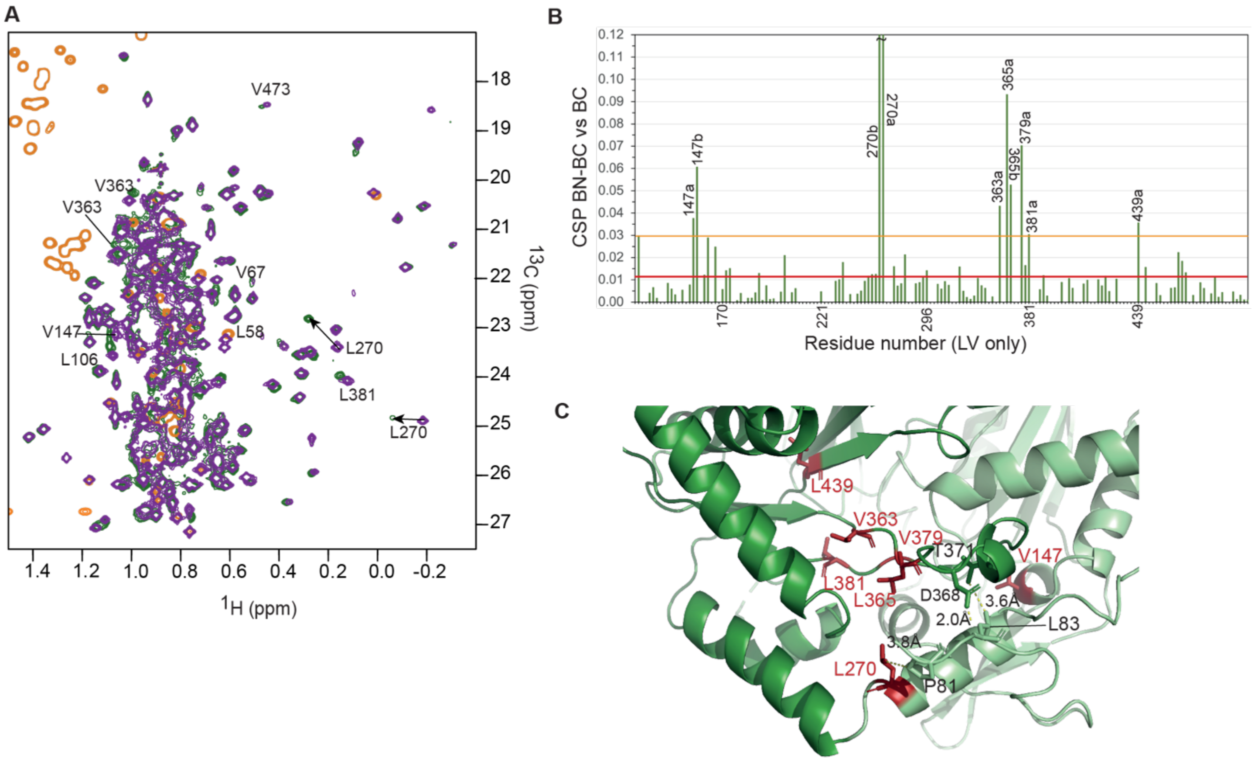

Fig. S5. BN behaves as an independent sub-domain in the BN-BC construct. (**A**) Overlay of the ^{1}H,^{13}C-methyl-HMQC spectra of BN-BC (purple), BC (green) and BN (orange). BN is uniformly ^{13}C labelled, rather than selectively ILV-methyl labelled, and thus its spectrum contains other side-chain signals besides those from ILV methyl groups. The close overlap of the combination of the BC and BN spectra with the spectrum of BN-BC demonstrates that BN maintains its fold in the context of BN-BC, in which it behaves as a largely independent sub-domain. Some well-resolved peaks are annotated. (**B**) Differences in chemical shifts (CSPs) between BC-LV methyl group peaks in the BN-BC and BC constructs. Large chemical shift differences are observed for L270. The mean of all CSPs (excluding L270) is indicated by the red line. The orange line indicates the mean + one standard deviation. **(C)** The methyl groups that exhibit the largest chemical shift differences between BN-BC and BC (higher than the orange line in panel B) are annotated and displayed in red on the BN-BC structure. These are the methyl groups of L270, which contacts P81 in the linker between the BC and the globular domain of BN, and those of V363, L365, V379 and L381 in the floor loop, whose position is also stabilized by contacts with the same linker in BN-BC (for example, through a hydrogen bond between the side-chain of D368 and the backbone amide of L83 and a hydrophobic contact between the methyl group of T371 and the side-chain of L83). V147 is also in contact with the floor loop.

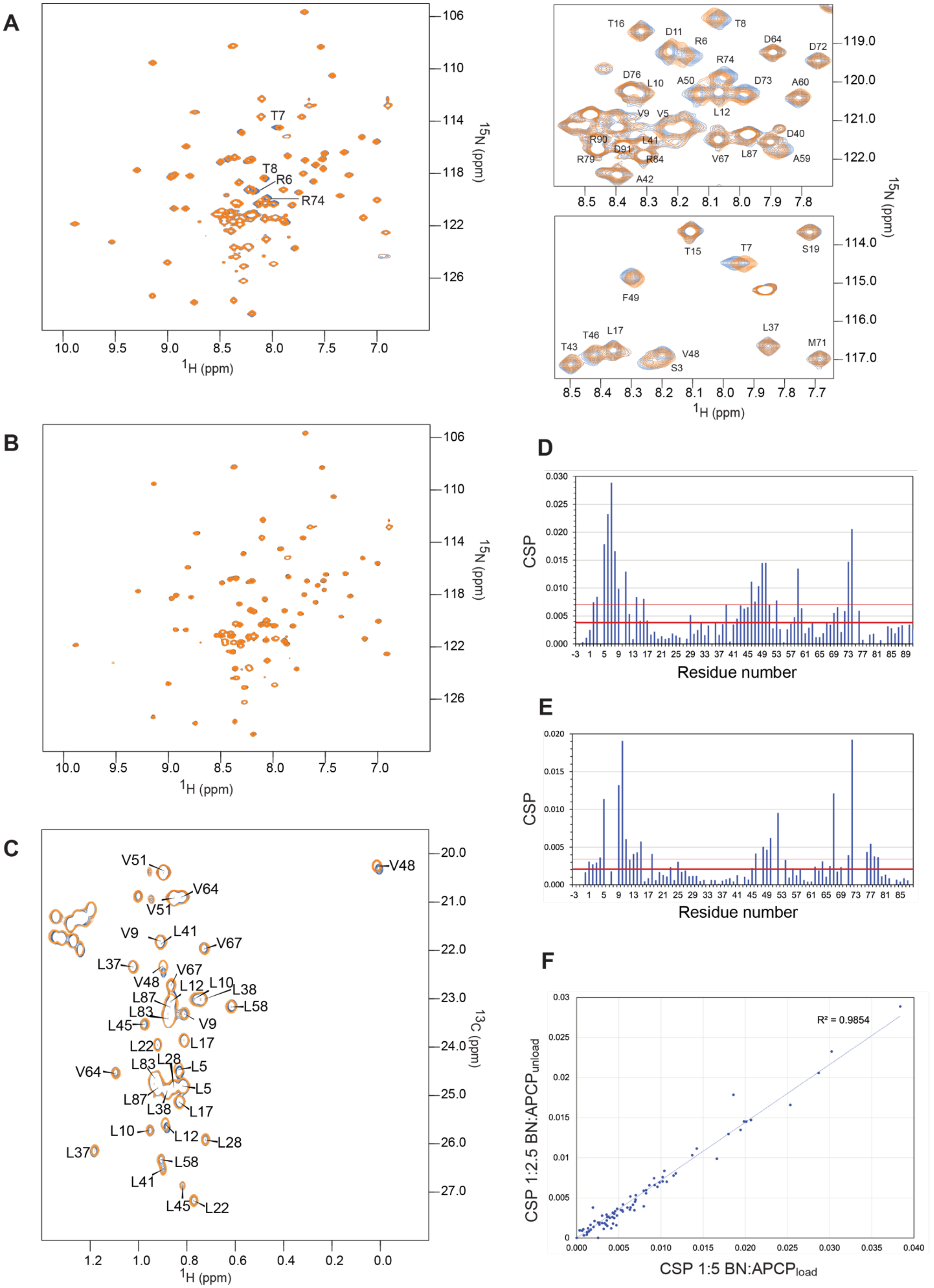

A
T7
T8
R6
R74
106
110
114
118
122
126
10.0
9.5
9.0
8.5
8.0
7.5
7.0
^{1}H (ppm)
^{15}N (ppm)
T16
D11
R6
T8
D64
D72
R74
D76
L10
A50
D73
A60
V9
V5
L12
R90
L41
D40
D91
L87
R79
R84
V67
A59
A42
119.0
120.0
121.0
122.0
8.5
8.4
8.3
8.2
8.1
8.0
7.9
7.8
T15
T7
S19
F49
L37
T46
L17
M71
T43
V48
S3
114.0
115.0
116.0
117.0
8.5
8.4
8.3
8.2
8.1
8.0
7.9
7.8
7.7
^{1}H (ppm)
^{15}N (ppm)
B
106
110
114
118
122
126
10.0
9.5
9.0
8.5
8.0
7.5
7.0
^{1}H (ppm)
^{15}N (ppm)
C
V51
V48
V64
V51
V9
L41
V67
L37
V67
V48
L12
L10
L38
L87
L58
L83
V9
L45
L22
L17
L83
L28
L5
V64
L5
L87
L38
L17
L10
L12
L28
L37
L58
L41
L45
L22
20.0
21.0
22.0
23.0
24.0
25.0
26.0
27.0
1.2
1.0
0.8
0.6
0.4
0.2
0.0
^{1}H (ppm)
^{13}C (ppm)
D
0.030
0.025
0.020
0.015
0.010
0.005
0.000
CSP
-3 1 5 9 13 17 21 25 29 33 37 41 45 49 53 57 61 65 69 73 77 81 85 89
Residue number
E
0.020
0.015
0.010
0.005
0.000
CSP
-3 1 5 9 13 17 21 25 29 33 37 41 45 49 53 57 61 65 69 73 77 81 85
Residue number
F
R^2 = 0.9854
0.03
0.025
0.02
0.015
0.01
0.005
0
0.000 0.005 0.010 0.015 0.020 0.025 0.030 0.035 0.040
CSP 1:2.5 BN:APCP$_{unload}$
CSP 1:5 BN:APCP$_{load}$

Fig. S6. BN interacts with APCP but not with BPCP. (A) Left, Overlay of ^{1}H,^{15}N-HSQC spectra of ^{15}N-labelled BN at a concentration of 100 μM in isolation (orange) and upon addition of 2.5 equivalents of $APCP_{ppant}$ (in blue). Peaks exhibiting the most significant CSPs are annotated. Right, expansions of two spectral regions of (A) displaying CSPs. **(B)** Overlay of ^{1}H,^{15}N-HSQC spectra of ^{15}N-labelled BN at a concentration of 100 μM in isolation (orange) and upon addition of 2.5 equivalents of $BPCP_{ppant}$ (in brown). (**C**) Overlay of ^{1}H,^{13}C-HMQC spectra of 100 μM ^{13}C,^{15}N-labelled BN in isolation (orange) and upon addition of 5 equivalents of $APCP_{load}$ (blue). (**D–E**) CSPs measured either for BN from the spectra shown in panel A (D) or for ^{15}N-labelled $APCP_{ppant}$ at a concentration of 80 μM upon addition of 2 molar equivalents of BN (E). The thick red line represents the 10%-trimmed mean of the CSPs, while the thin orange line represents one standard deviation from the trimmed mean. (**F**) Correlation of the CSPs measured from the spectra shown in panel A with those measured from ^{1}H,^{15}N-HSQC spectra of 100 μM ^{13}C,^{15}N-labelled BN upon addition of 5 molar equivalents of $APCP_{load}$. The value of the squared Pearson correlation coefficient (R^2) is given in the panel.

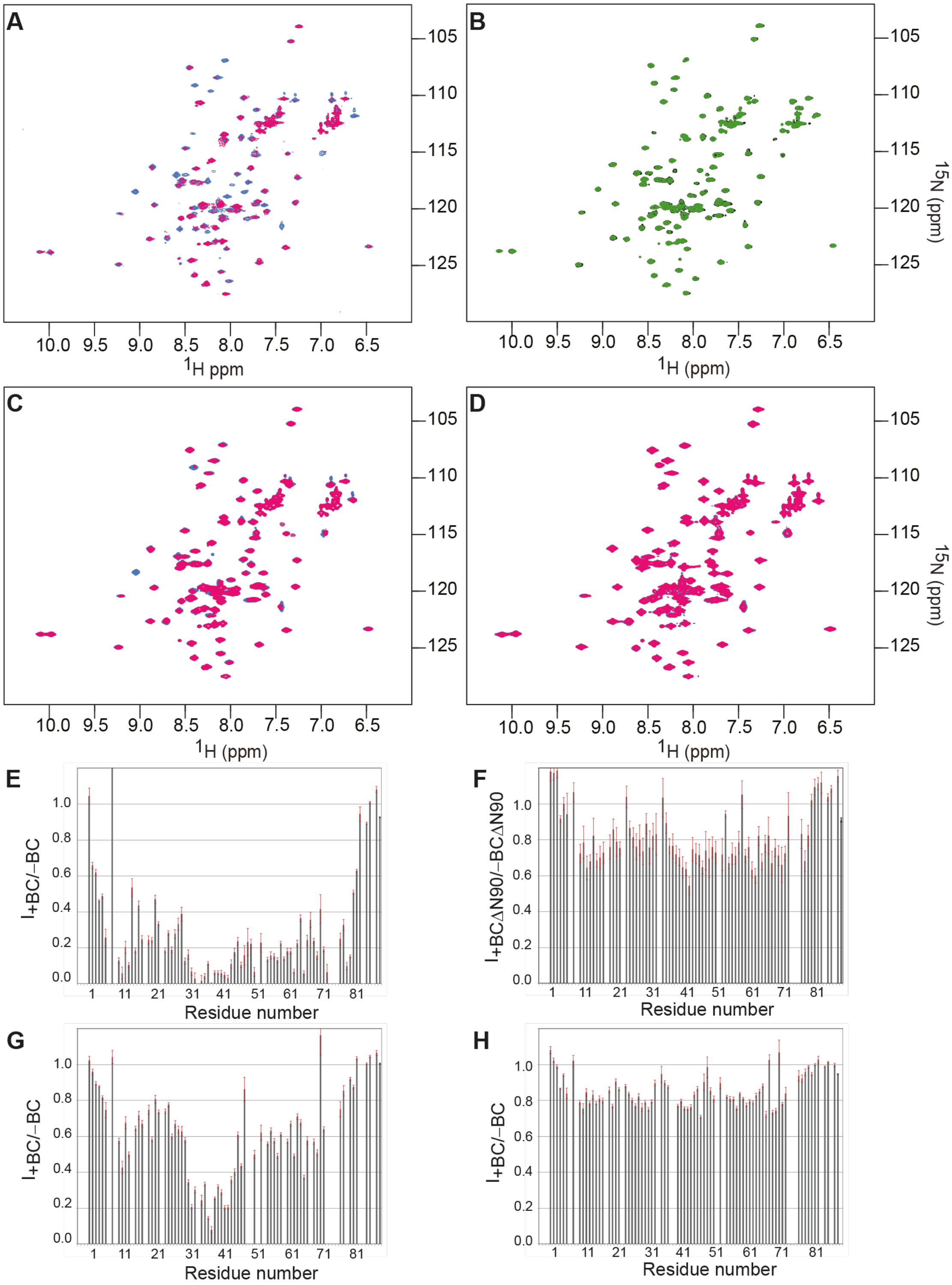

Fig. S7. BN is needed for the recruitment of APCP to BN-BC. (**A**) Overlay of the $^{1}H,^{15}N$-HSQC spectra of 120 µM ^{15}N-labelled $APCP_{load}$ alone (light blue) and in the presence of 1.5 equivalents of BN-BC (magenta). (**B**) Overlay of the $^{1}H,^{15}N$-HSQC spectra of 120 µM ^{15}N-labelled $APCP_{load}$ alone (light blue) and in the presence of 1.5 equivalents of BC (green). (**C**) Overlay of the $^{1}H,^{15}N$-HSQC spectra of 120 µM ^{15}N-labelled $APCP_{ppant}$ alone (light blue) and in the presence of 1.5

equivalents of BN-BC (magenta). (**D**) Overlay of the ^{1}H,^{15}N-HSQC spectra of 120 μM ^{15}N-labelled APCP mutant S37A alone (light blue) and in the presence of 1.5 equivalents of BN-BC (magenta). (**E–F**) Relative intensities of $APCP_{load}$ peaks in the presence of either BN-BC (E) or BC (F) with respect to peak intensities of $APCP_{load}$ alone. (**G–H**) Relative intensities of $APCP_{ppant}$ (G) and APCP-S37A mutant (H) peaks in the presence of BN-BC with respect to the peak intensities of, respectively, $APCP_{ppant}$ and the APCP-S37A mutant alone. The amino acids before residue number 1 are left at the protein N-terminus after HRV3C protease cleavage.

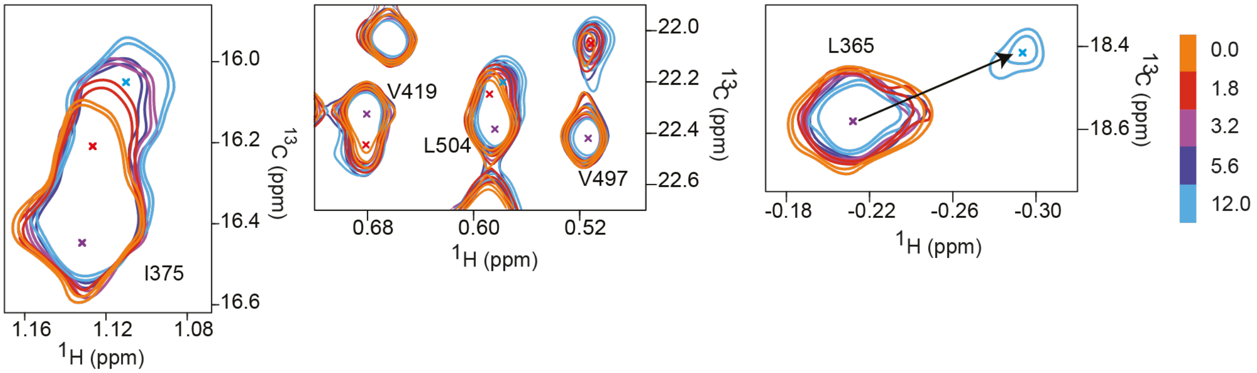

Fig. S8. BN-BC interacts with APCP$_{load}$ with weak affinity. Overlay of excerpts of ^{1}H,^{13}C-methyl-HMQC spectra of 50 μM ^{1}H,^{13}C-ILV-methyl-labelled ^{2}H-BN-BC upon addition of 1.8–12 equivalents of APCP$_{load}$. The spectra are color-coded according to the number of APCP$_{load}$ equivalents, as indicated by the key on the right. Shown are five peaks belonging to the BC domain. The positions and intensities of the peaks continue to change up to the final addition of APCP$_{load}$ showing that BN-BC was not fully bound even at 12 equivalents of APCP$_{load}$. The color-code of the crosses marking the peaks is the same as in Fig. 3.

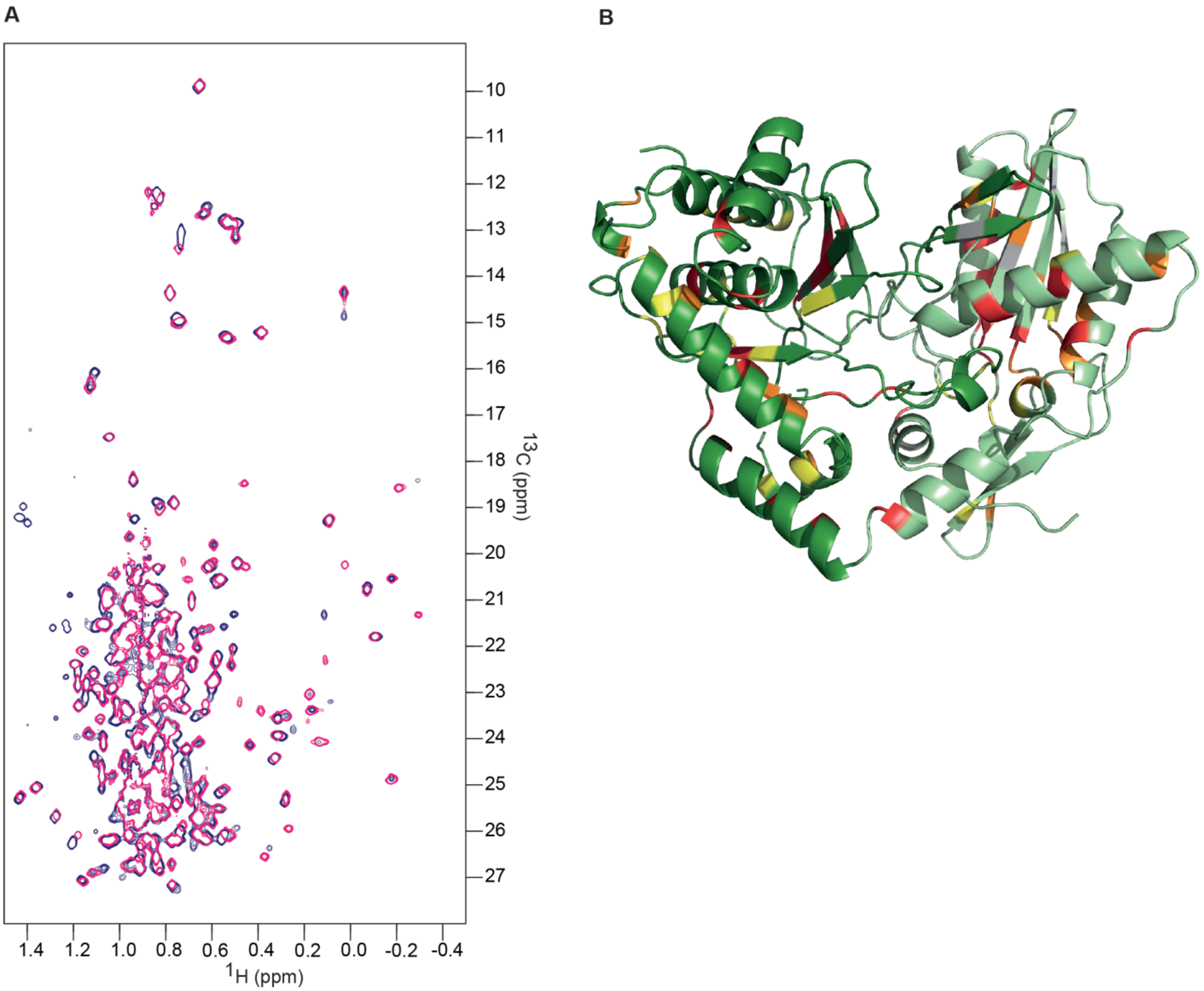

Fig. S9. Binding of APCP$_{load}$ causes widespread and significant CSPs throughout the BC domain. (**A**) Overlay of the ^{1}H,^{13}C-methyl-HMQC spectra of 100 µM ^{1}H,^{13}C-ILV-methyl-labelled ^{2}H-BN-BC in isolation (magenta) and after addition of 12 equivalents of APCP$_{load}$ (blue). (**B**) CSPs of the BN-BC methyl-group peaks upon addition of APCP$_{load}$ (calculated from the spectra in panel A) mapped onto the crystallographic structure of BN-BC. The color-code is as follows: grey, ILV residues for which the position and intensity of the methyl-group peaks do not change; yellow, ILV residues whose methyl groups show two conformations, and for which either the intensity or both the intensity and position of the minor-state methyl-group peak change upon addition of APCP$_{load}$; orange and red, ILV residues whose methyl-group peaks display either moderate (≤ linewidth, orange) or substantial (> linewidth, red) CSPs upon addition of APCP$_{load}$.

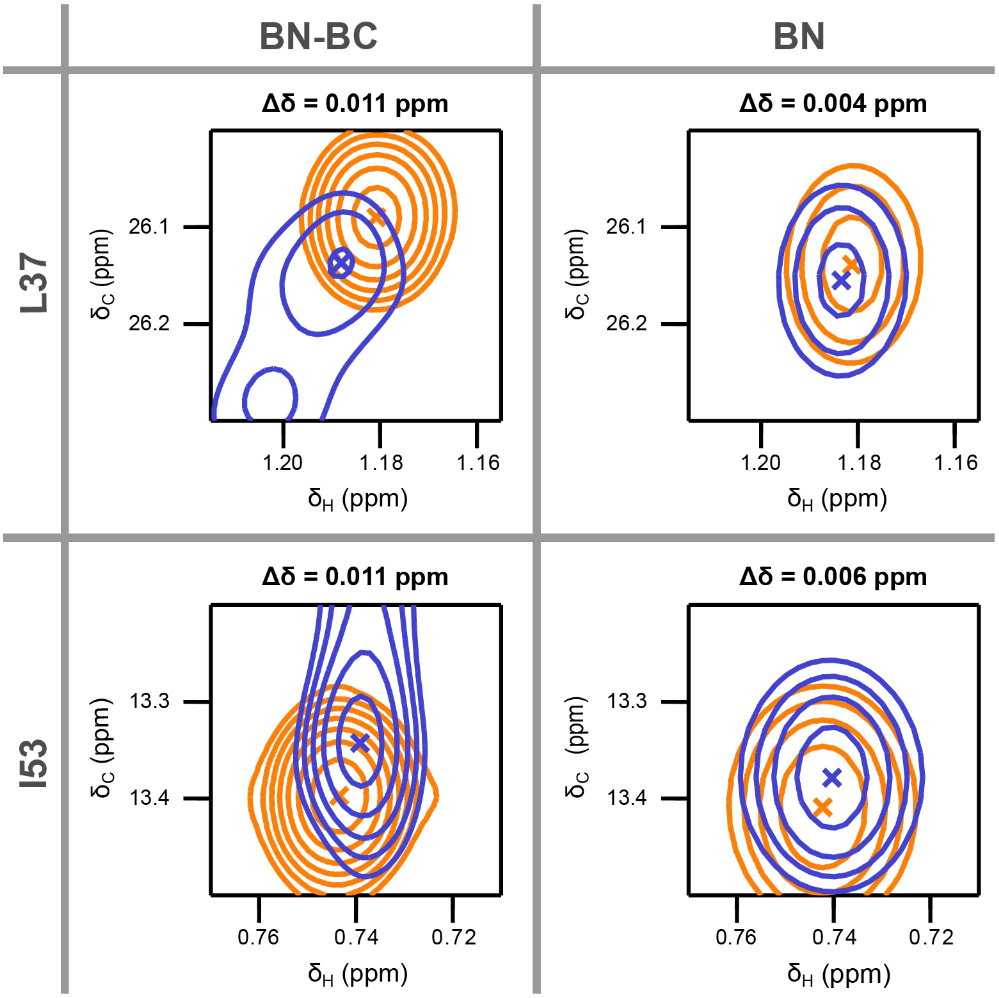

Fig. S10. The $APCP_{load}$-induced fast-exchange CSPs within the BN domain of the BN-BC construct correspond to the $APCP_{load}$-induced CSPs of isolated BN. Methyl-group CSPs of residues L37 (top) and I53 (bottom) upon addition of $APCP_{load}$ to BN-BC (left) and BN (right). In all panels, the apo spectrum is shown in orange, and the spectrum after addition of $APCP_{load}$ is show in blue. For the BN-BC spectra, the BN-BC concentration was 50 µM; $APCP_{load}$ was added to a $APCP_{load}$:BN-BC molar ratio of 5.6:1. For the BN spectra, the BN concentration was 100 µM; $APCP_{load}$ was added to an $APCP_{load}$:BN molar ratio of 5:1.

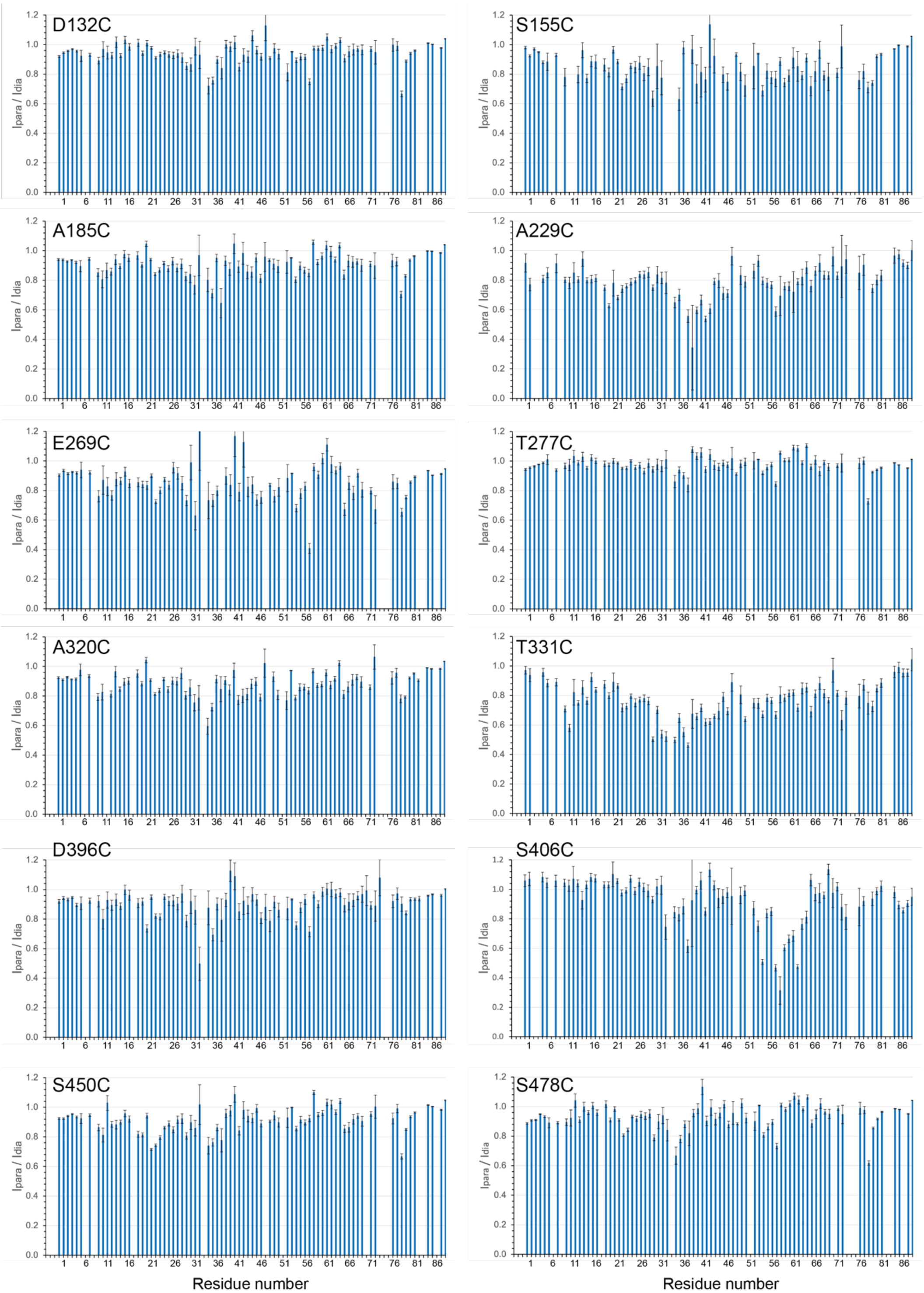

Fig. S11. Paramagnetic relaxation enhancement effects observed on APCP$_{load}$. Quantification of I_{para}/I_{dia} for paramagnetic relaxation enhancement experiments performed by recording $^{1}H,^{15}N$-HSQC spectra of 120 μM APCP$_{load}$ in the presence of 1.5 molar equivalents of paramagnetically tagged BN-BC. Paramagnetic tags (spin-labels) were coupled to each of 12 mutant BN-BC constructs containing a single cysteine, as indicated in each panel. Peak intensities were taken as

the peak heights (without line-shape fitting). This first approximate quantification was used to determine which paramagnetic tag positions yielded the greatest PRE effects on $APCP_{load}$. The amino acids before residue number 1 are left at the protein N-terminus after HRV3C protease cleavage.

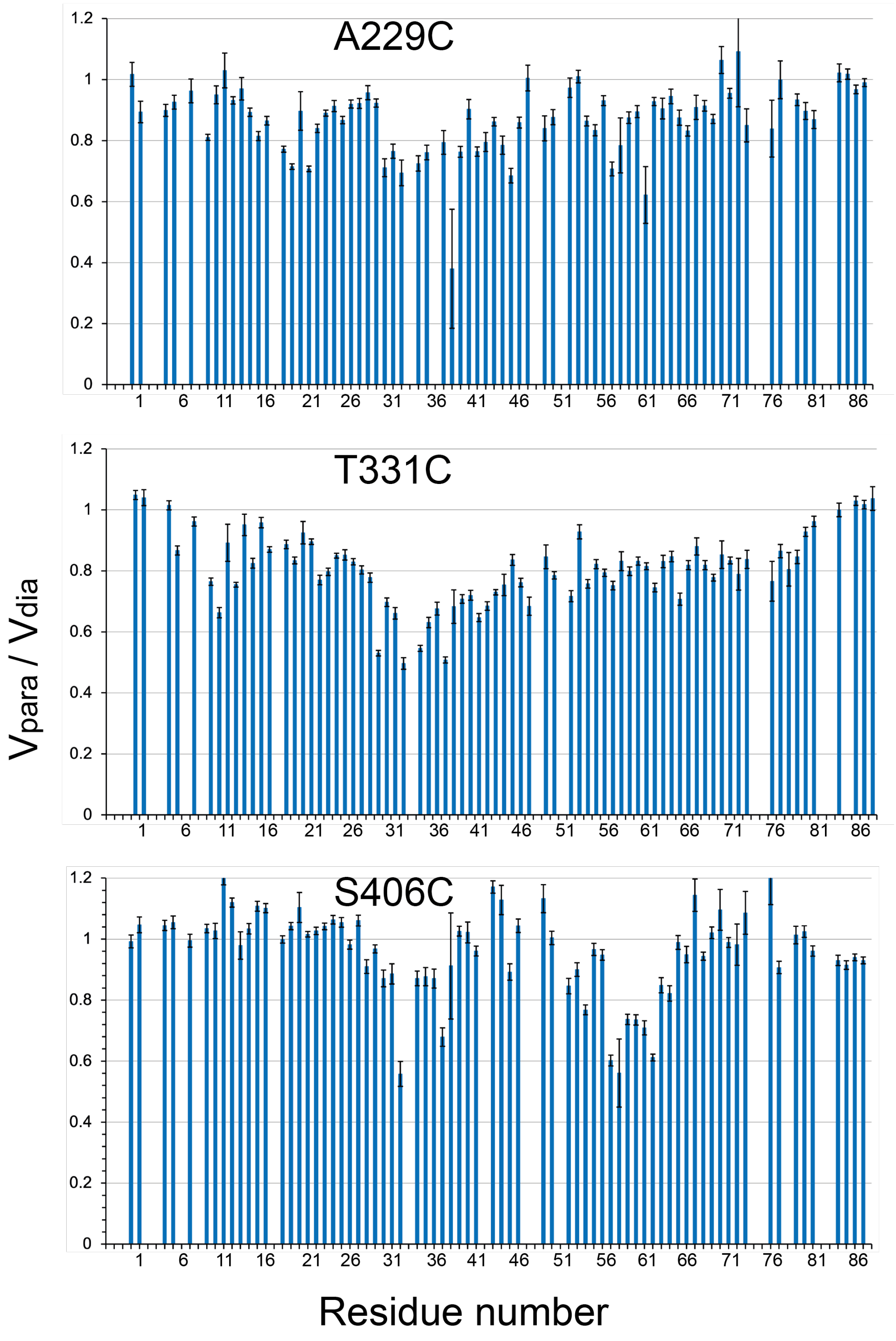

Fig. S12. Paramagnetic relaxation enhancement effects observed on APCP$_{load}$ for paramagnetic tags coupled to three BN-BC mutants. Quantification of PRE effects in ^{1}H,^{15}N-HSQC spectra of 120 μM APCP$_{load}$ in the presence of 1.5 molar equivalents of paramagnetically tagged BN-BC (same experiments as in fig. S11). The paramagnetic tag was coupled to each of three BN-BC mutant constructs: A229C, T331C and S406C. Peak volumes and linewidths of both

the paramagnetic and diamagnetic spectra were extracted using the lineshape-fitting software FuDA (https://www.ucl.ac.uk/hansen-lab/fuda/).

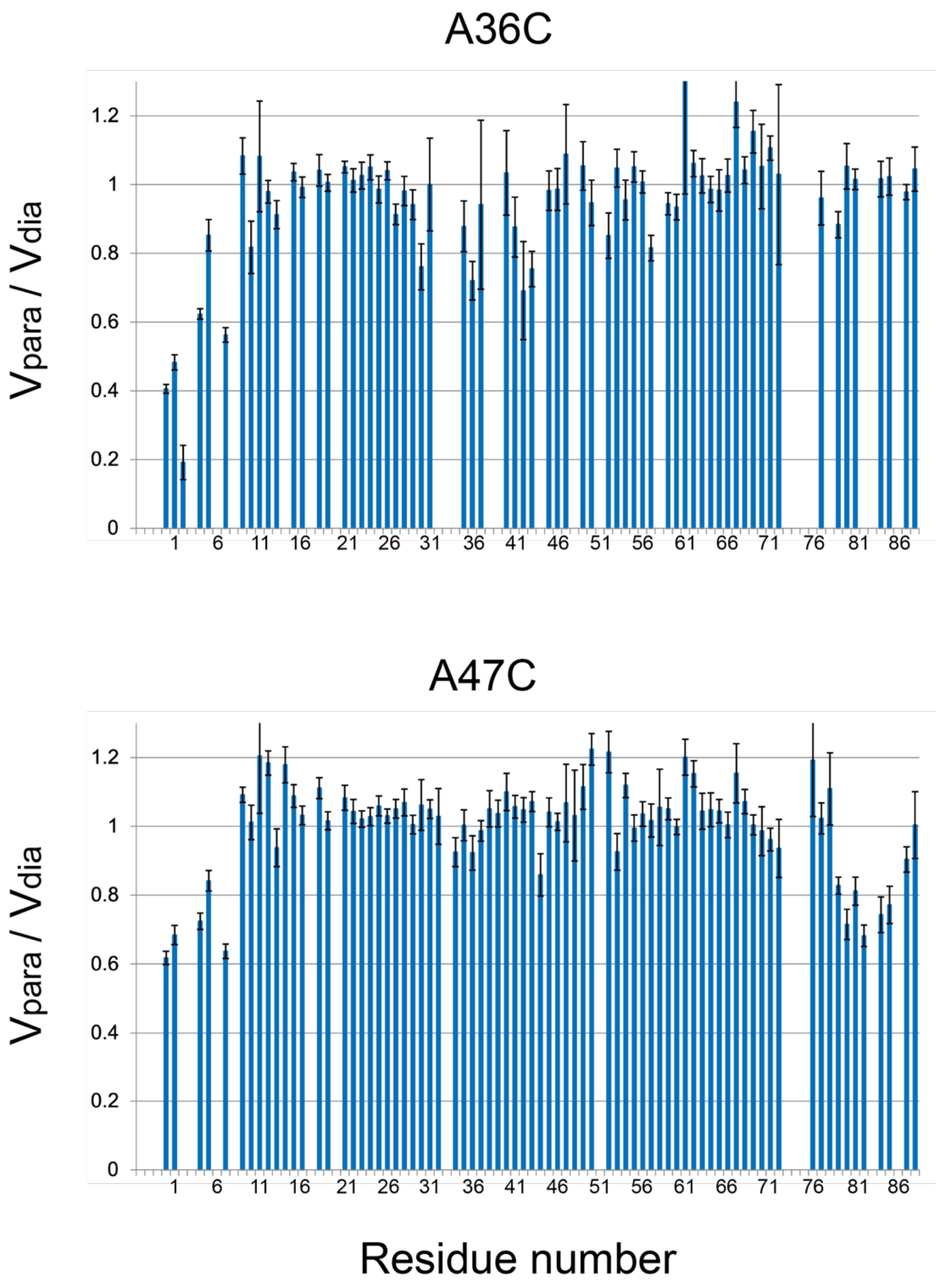

Fig. S13. Paramagnetic relaxation enhancement effects observed on APCP$_{load}$ for paramagnetic tags coupled to two BN-BC mutants in the BN domain. Quantification of PRE effects in ^{1}H,^{15}N-HSQC spectra of 100 μM APCP$_{load}$ in the presence of 1.5 molar equivalents of paramagnetically tagged BN-BC. The paramagnetic tag was coupled to each of two BN-BC mutant constructs: A36C and A47C. The amino acids before residue number 1 are left at the protein N-terminus after HRV3C protease cleavage. Peak volumes and linewidths of both the paramagnetic and diamagnetic spectra were extracted using the lineshape-fitting software FuDA (https://www.ucl.ac.uk/hansen-lab/fuda/).

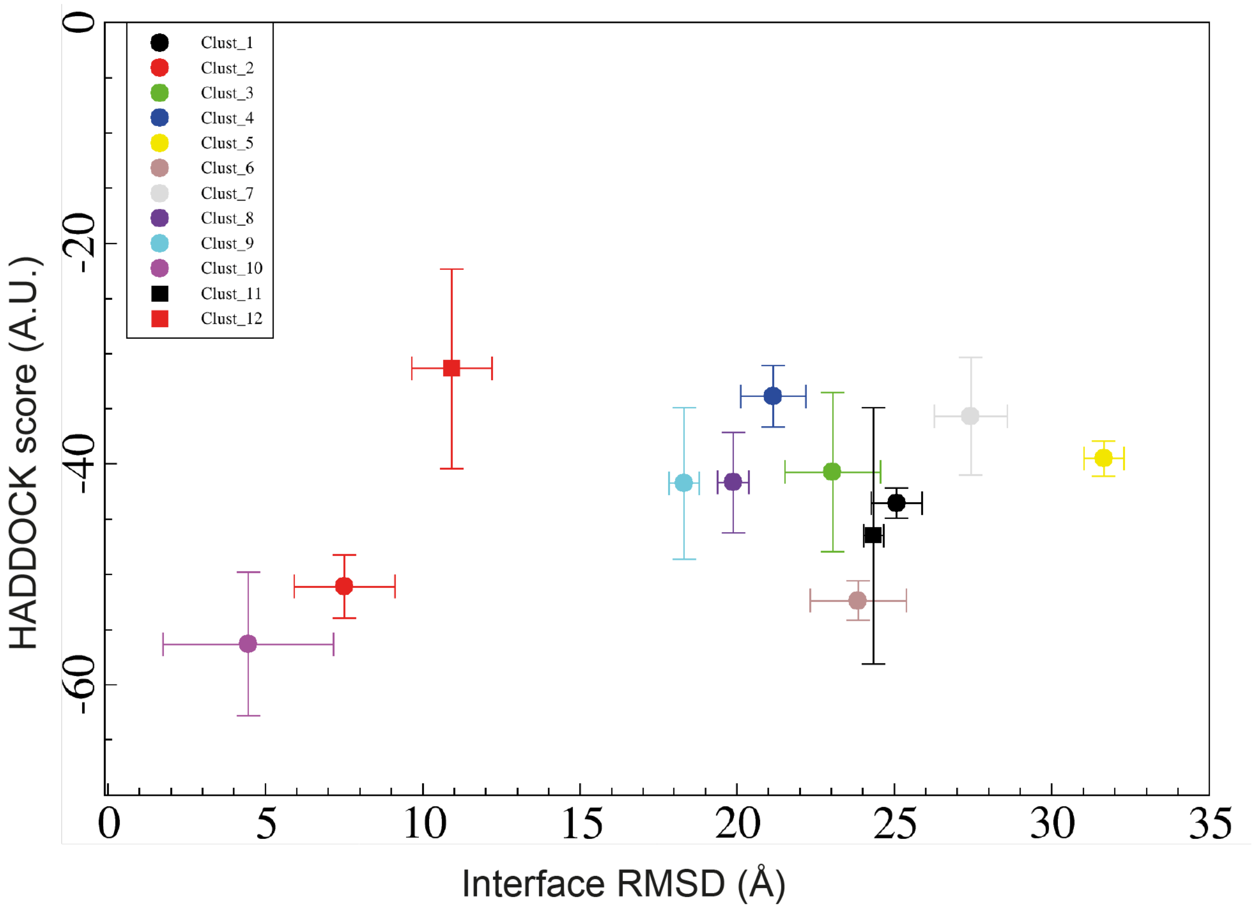

Fig. S14. Cluster analysis of the structures derived by docking BN to the BC–APCP$_{load}$ complex. The plot shows the correlation between il-RMSD and HADDOCK score for the set of 12 clusters derived from the complete ensemble of water-refined structures. The interface il-RMSD is calculated with respect to the lowest-scoring clustered structure (with the interface corresponding to that between BN and the BC–APCP$_{load}$ complex). The round or square markers correspond to the average values of the four lowest-scoring structures of each cluster, with the error bars representing the corresponding standard deviations over those four structures.

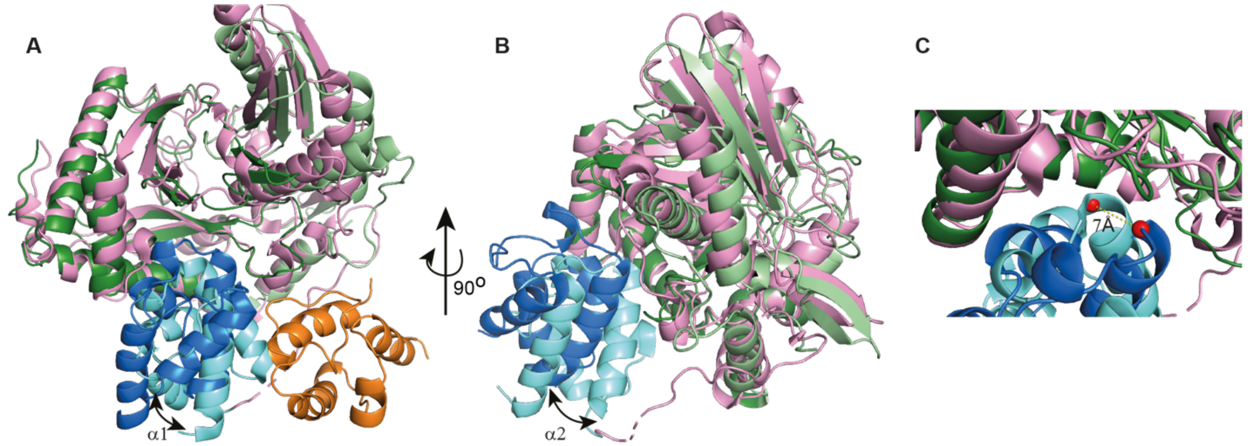

Fig. S15. Comparison of the structure of the BN-BC–APCP$_{load}$ complex with the structure of the LgrA NRPS. (A–B) Overlay of the structure of the BN-BC–APCP$_{load}$ complex determined here and the structure of the substrate-loaded donor PCP bound to the C domain from a five-domain construct of LgrA (PDB ID 6MFY). The structures have been aligned on the C lobe of BC. LgrA-C domain, pink; LgrA-PCP donor domain, cyan; TomB C domain (BC), green; TomA PCP$_{load}$ (APCP$_{load}$), marine blue; TomB N domain (BN), orange. The structures in A and B are related by a rotation of 90º around the vertical axis. The BN domain is omitted in B for clarity. The arrows next to the structures indicate the relative rotations of helix α1 and α2 between the two structures. **(C)** Expanded view of (A) showing the position of the C$^{\alpha}$ atom of the serine residue carrying the ppant arm in the donor PCPs of the two structures.

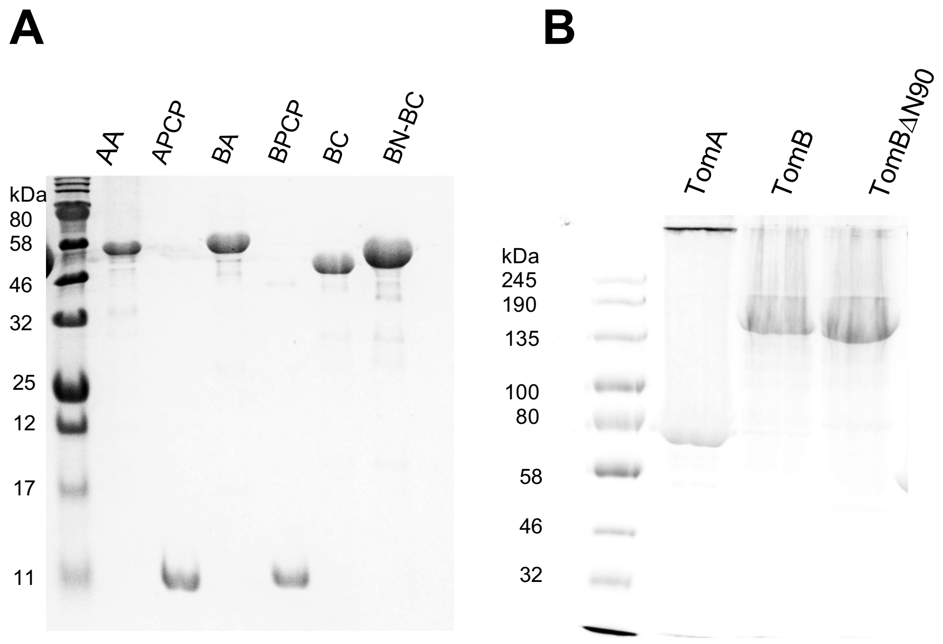

Fig. S16 SDS-PAGE analysis of proteins used in the activity assays. (A) SDS-PAGE of excised domains used in the activity assays: AA (55.2 kDa); APCP (10.4 kDa); BA (56.6 kDa); BPCP (9.9 kDa); BC (49.8 kDa); BN–BC (59.2 kDa). **(B)** SDS-PAGE of full-length proteins used in the activity assays: TomA (65.1 kDa); TomB (167.1 kDa); TomBΔN90 (157.8 kDa). Protein markers with sizes in kDa are shown on the left-hand side of each gel.

Table S1. X-ray crystallography data collection and refinement statistics. Statistics for the highest-resolution shell are shown in parentheses.

	**TomBC_native**	**TomBC_SeMet**
**Wavelength**		
**Resolution range**	19.85–1.65 (1.709–1.65)	48.91–2.3 (2.4–2.3)
**Space group**	F 2 2 2	C 1 2 1
**Unit cell**	121.65 160.06 163.9<br>90 90 90	123.35 160.87 100.15<br>90 125.109 90
**Total reflections**	394767 (16074)	147267 (179240)
**Unique reflections**	81329 (2759)	139112 (16569)
**Multiplicity**	4.9 (5.8)	10.6 (10.8)
**Completeness (%)**	0.85 (0.29)	0.99 (0.99)
**Mean I/sigma(I)**	12.43 (2.01)	9.9(2.1)
**Wilson B-factor**	27.88	32.48
**R-merge**	0.06379 (0.7985)	
**R-meas**	0.07165 (0.8738)	
**CC1/2**	0.997 (0.694)	0.994 (0.747)
**CC***	0.999 (0.905)	
**Reflections used in refinement**	81326 (2759)	70285 (6946)
**Reflections used for R-free**	4067 (138)	3515 (347)
**R-work**	0.1771 (0.3097)	0.172 (0.2341)
**R-free**	0.1968 (0.3344)	0.216 (0.2943)
**Number of non-hydrogen atoms**	4548	7521
**macromolecules**	4134	6901

**ligands**	54	34
**Protein residues**	448	881
**RMS(bonds)**	0.015	0.011
**RMS(angles)**	1.42	1.01
**Ramachandran favored (%)**	97.3	97.02
**Ramachandran allowed (%)**	2.7	2.98
**Ramachandran outliers (%)**	0.0	0
**Rotamer outliers (%)**	1.8	0.71
**Clashscore**	6.6	3.57
**Average B-factor**	38.35	37.79
**macromolecules**	37.59	34.97
**ligands**	54.27	61.83
**solvent**	44.64	40.95
**PDB code**	8QNF	8RZ6

Table S2. Statistics of the NMR structures

	$APCP_{ppant}$	$APCP_{load}$	BN
**Restraint counts**			
Unique distance restraints	1843	1857	1593
Distance restraint type			
Unambiguous	1507	1439	1350
Ambiguous	336	418	243
Intra-residue [i = j]<br>Sequential [| i–j| = 1]<br>Medium range [1 < |i–j| ≤ 5]<br>Long range [|i–j| > 5]	696.7<br>382.9<br>377.2<br>386.3	700.7<br>346.5<br>337.1<br>472.8	758.6<br>339.6<br>242.5<br>252.2
Dihedral angle restraints (ϕ and ψ)	152	136	170
**Structural quality parameters**			
**Structural RMSD (fitting to mean) (Ensemble average)**	aa 6–79	aa 6–79	aa 4–74
Backbone	0.99 (±0.17)	0.41 (±0.08)	0.51 (±0.09)
Heavy atoms	1.24 (±0.13)	0.93 (±0.18)	0.99 (±0.12)
**RMSDs from experimental restraints**			
NOE distances (Å)	0.18 (±0.02)	0.017 (±0.001)	0.017(±0.002)
Dihedral angles (°)	1.3 (±0.2)	0.4 (±0.1)	1.30 (±0.08)
Number violated > 0.5 Å	1	0	0
Dihedral angles > 5°	2.80 (±0.97)	0.00	3.30 (±0.64)
**RMSDs from idealized geometry**			
Bond-lengths (Å)	0.00357 (±0.00006)	0.00313 (±0.00006)	0.0033 (±0.0001)
Bond angles (°)	0.56 (±0.03)	0.502 (±0.008)	0.52 (±0.02)
**Assignment coverage**			
Backbone nuclei (%)			
H^N	96.6	95.5	100
N	91.3	91.3	91.6
C`	89.1	0	91.6
C^α	97.8	97.8	98.9
H^α	97.0	97.0	96.0
Side-chain by element (%)			
H	86.9	83.1	85.8
C/N	75.5	71.0	71.0
PDB ID	8QPY	8QRX	8QSX
BMRB Entry ID	34868	34869	34870

Table S3. Relative intensity change of APCP amide resonances upon addition of 1.5 molar equivalents of BN-BC. $APCP_{load}\Delta C11$ and $APCP_{load}\Delta N5$ indicate APCP constructs missing the last 11 and first 5 residues, respectively.

**APCP construct**	**BN-BC construct**	**Relative intensity change (± SD)**
$APCP_{load}$	Wild-type	0.279 ± 0.003
$APCP_{ppant}$	Wild-type	0.620 ± 0.003
APCP-S37A	Wild-type	0.858 ± 0.002
$APCP_{load}$-F61S	Wild-type	0.734 ± 0.002
$APCP_{load}$	BC-D11A/H25A	0.375 ± 0.002
$APCP_{load}$-Q13V/K49V	Wild-type	0.401 ± 0.002
$APCP_{load}\Delta C11$	Wild-type	0.295 ± 0.001
$APCP_{load}\Delta N5$	Wild-type	0.299 ± 0.001

Table S4. Paramagnetic relaxation enhancement effects used in the calculation of the structure of the BN-BC-APCP$_{load}$ complex.

Paramagnetic tag position	Protein observed	Residue affected	Peak quantified	I_{para}/I_{dia}
BN-BC-C36	APCP$_{load}$	7	NH	0.56 ± 0.02
BN-BC-C229	APCP$_{load}$	18	NH	0.77 ± 0.01
BN-BC-C229	APCP$_{load}$	19	NH	0.72 ± 0.01
BN-BC-C229	APCP$_{load}$	21	NH	0.71 ± 0.01
BN-BC-C229	APCP$_{load}$	30	NH	0.71 ± 0.03
BN-BC-C229	APCP$_{load}$	31	NH	0.77 ± 0.02
BN-BC-C229	APCP$_{load}$	32	NH	0.69 ± 0.04
BN-BC-C229	APCP$_{load}$	34	NH	0.73 ± 0.03
BN-BC-C229	APCP$_{load}$	35	NH	0.76 ± 0.02
BN-BC-C229	APCP$_{load}$	37	NH	0.79 ± 0.04
BN-BC-C229	APCP$_{load}$	38	NH	0.38 ± 0.20
BN-BC-C229	APCP$_{load}$	39	NH	0.76 ± 0.02
BN-BC-C229	APCP$_{load}$	41	NH	0.76 ± 0.02
BN-BC-C229	APCP$_{load}$	44	NH	0.79 ± 0.03
BN-BC-C229	APCP$_{load}$	45	NH	0.69 ± 0.02
BN-BC-C229	APCP$_{load}$	57	NH	0.71 ± 0.02
BN-BC-C229	APCP$_{load}$	58	NH	0.78 ± 0.09
BN-BC-C229	APCP$_{load}$	61	NH	0.62 ± 0.09
BN-BC-C331	APCP$_{load}$	9	NH	0.77 ± 0.01
BN-BC-C331	APCP$_{load}$	10	NH	0.66 ± 0.02
BN-BC-C331	APCP$_{load}$	12	NH	0.76 ± 0.01
BN-BC-C331	APCP$_{load}$	22	NH	0.77 ± 0.02
BN-BC-C331	APCP$_{load}$	28	NH	0.78 ± 0.02
BN-BC-C331	APCP$_{load}$	29	NH	0.53 ± 0.01
BN-BC-C331	APCP$_{load}$	30	NH	0.70 ± 0.01
BN-BC-C331	APCP$_{load}$	31	NH	0.66 ± 0.02
BN-BC-C331	APCP$_{load}$	32	NH	0.50 ± 0.02
BN-BC-C331	APCP$_{load}$	34	NH	0.55 ± 0.01
BN-BC-C331	APCP$_{load}$	35	NH	0.63 ± 0.02
BN-BC-C331	APCP$_{load}$	36	NH	0.68 ± 0.02
BN-BC-C331	APCP$_{load}$	37	NH	0.51 ± 0.01
BN-BC-C331	APCP$_{load}$	38	NH	0.68 ± 0.06
BN-BC-C331	APCP$_{load}$	39	NH	0.71 ± 0.01
BN-BC-C331	APCP$_{load}$	40	NH	0.72 ± 0.02

BN-BC-C331	$APCP_{load}$	41	NH	0.65 ± 0.01
BN-BC-C331	$APCP_{load}$	42	NH	0.69 ± 0.01
BN-BC-C331	$APCP_{load}$	43	NH	0.73 ± 0.01
BN-BC-C331	$APCP_{load}$	44	NH	0.75 ± 0.04
BN-BC-C331	$APCP_{load}$	46	NH	0.76 ± 0.01
BN-BC-C331	$APCP_{load}$	47	NH	0.68 ± 0.03
BN-BC-C331	$APCP_{load}$	50	NH	0.79 ± 0.01
BN-BC-C331	$APCP_{load}$	52	NH	0.72 ± 0.02
BN-BC-C331	$APCP_{load}$	54	NH	0.76 ± 0.01
BN-BC-C331	$APCP_{load}$	56	NH	0.79 ± 0.01
BN-BC-C331	$APCP_{load}$	57	NH	0.75 ± 0.01
BN-BC-C331	$APCP_{load}$	62	NH	0.75 ± 0.01
BN-BC-C331	$APCP_{load}$	65	NH	0.71 ± 0.02
BN-BC-C331	$APCP_{load}$	69	NH	0.78 ± 0.01
BN-BC-C331	$APCP_{load}$	72	NH	0.79 ± 0.05
BN-BC-C331	$APCP_{load}$	76	NH	0.77 ± 0.07
BN-BC-C406	$APCP_{load}$	32	NH	0.56 ± 0.04
BN-BC-C406	$APCP_{load}$	37	NH	0.68 ± 0.03
BN-BC-C406	$APCP_{load}$	54	NH	0.77 ± 0.02
BN-BC-C406	$APCP_{load}$	57	NH	0.60 ± 0.02
BN-BC-C406	$APCP_{load}$	58	NH	0.56 ± 0.11
BN-BC-C406	$APCP_{load}$	59	NH	0.74 ± 0.02
BN-BC-C406	$APCP_{load}$	60	NH	0.74 ± 0.02
BN-BC-C406	$APCP_{load}$	61	NH	0.71 ± 0.02
BN-BC-C406	$APCP_{load}$	62	NH	0.61 ± 0.01
$APCP_{load}$-C17	BN-BC	10	$C^{\delta2}H^{\delta2}$	0.30 ± 0.10
$APCP_{load}$-C17	BN-BC	22	$C^{\delta1}H^{\delta1}$	0.48 ± 0.06
$APCP_{load}$-C17	BN-BC	22	$C^{\delta2}H^{\delta2}$	0.35 ± 0.01
$APCP_{load}$-C17	BN-BC	28	$C^{\delta1}H^{\delta1}$	0.74 ± 0.16
$APCP_{load}$-C17	BN-BC	37	$C^{\delta1}H^{\delta1}$	0.70 ± 0.04
$APCP_{load}$-C17	BN-BC	58	$C^{\delta1}H^{\delta1}$	0.81 ± 0.03
$APCP_{load}$-C17	BN-BC	64	$C^{\gamma1}H^{\gamma1}$	0.27 ± 0.11
$APCP_{load}$-C17	BN-BC	64	$C^{\gamma2}H^{\gamma2}$	0.27 ± 0.11
$APCP_{load}$-C17	BN-BC	233	$C^{\gamma2}H^{\gamma2}$	0.62 ± 0.06
$APCP_{load}$-C17	BN-BC	449	$C^{\delta1}H^{\delta1}$	0.72 ± 0.01
$APCP_{load}$-C45	BN-BC	233	$C^{\gamma2}H^{\gamma2}$	0.48 ± 0.16
$APCP_{load}$-C45	BN-BC	270	$C^{\delta1}H^{\delta1}$	0.56 ± 0.03
$APCP_{load}$-C45	BN-BC	270	$C^{\delta2}H^{\delta2}$	0.35 ± 0.03
$APCP_{load}$-C45	BN-BC	276	$C^{\delta2}H^{\delta2}$	0.63 ± 0.04

$APCP_{load}$-C45	BN-BC	280	$C^{\delta 1}H^{\delta 1}$	0.75 ± 0.04
$APCP_{load}$-C45	BN-BC	280	$C^{\delta 2}H^{\delta 2}$	0.86 ± 0.04
$APCP_{load}$-C45	BN-BC	365	$C^{\delta 2}H^{\delta 2}$	0.60 ± 0.03

Table S5. List of all mutant constructs generated in this work. The name of each plasmid in in the laboratory user database is provided to facilitate plasmid request. Information on the antibiotic resistance, affinity tag added for protein purification and protease cleavage site is given in the last two columns.

**Plasmid arbitrary number**	**Name of plasmid in the manuscript**	**Name of plasmid in USER database**	**Antibiotic Resistance**	**Tag**
**1**	APCP-Q13VK49V	pETM44 TomAPCP-Q17VK53V	Kan	N-6XHis/N-MBP/3C-site
**2**	APCP-D17C	pETM44 TomAPCP-D21C	Kan	N-6XHis/N-MBP/3C-site
**3**	APCP-S37A	pETM44 TomAPCP-S41A	Kan	N-6XHis/N-MBP/3C-site
**4**	APCP-S45C	pETM44 TomAPCP-S49C	Kan	N-6XHis/N-MBP/3C-site
**5**	APCP-F61S	pETM44 TomAPCP-F65S	Kan	N-6XHis/N-MBP/3C-site
**6**	APCP-E67C	pETM44 TomAPCP-E71C	Kan	N-6XHis/N-MBP/3C-site
**7**	APCP-ΔC11	pETM44 TomAPCP-delC11	Kan	N-6XHis/N-MBP/3C-site
**8**	APCP-ΔN5	pETM44 TomAPCP-delN5	Kan	N-6XHis/N-MBP/3C-site
**9**	BN-BC-C32S	pETM44 TomBC C36S	Kan	N-6XHis/N-MBP/3C-site
**10**	BN-BC-C32S-A36C	pETM44 TomBC C36S_A40C	Kan	N-6XHis/N-MBP/3C-site
**11**	BN-BC-C32S-A47C	pETM44 TomBC C36S_A51C	Kan	N-6XHis/N-MBP/3C-site
**12**	BN-BC-C32S-S102C	pETM44 TomBC C36S_S106C	Kan	N-6XHis/N-MBP/3C-site
**13**	BN-BC-C32S-A132C	pETM44 TomBC C36S_A136C	Kan	N-6XHis/N-MBP/3C-site
**14**	BN-BC-C32S-S155C	pETM44 TomBC C36S_S159C	Kan	N-6XHis/N-MBP/3C-site
**15**	BN-BC-C32S-A185C	pETM44 TomBC C36S_A189C	Kan	N-6XHis/N-MBP/3C-site
**16**	BN-BC-C32S-A229C	pETM44 TomBC C36S_A233C	Kan	N-6XHis/N-MBP/3C-site
**17**	BN-BC-C32S-E269C	pETM44 TomBC C36S_E273C	Kan	N-6XHis/N-MBP/3C-site
**18**	BN-BC-C32S-T277C	pETM44 TomBC C36S_T281C	Kan	N-6XHis/N-MBP/3C-site
**19**	BN-BC-C32S-A320C	pETM44 TomBC C36S_A324C	Kan	N-6XHis/N-MBP/3C-site
**20**	BN-BC-C32S-T331C	pETM44 TomBC C36S_T335C	Kan	N-6XHis/N-MBP/3C-site
**21**	BN-BC-C32S-D396C	pETM44 TomBC C36S_D400C	Kan	N-6XHis/N-MBP/3C-site
**22**	BN-BC-C32S-A406C	pETM44 TomBC C36S_S410C	Kan	N-6XHis/N-MBP/3C-site
**23**	BN-BC-C32S-T450C	pETM44 TomBC C36S_S454C	Kan	N-6XHis/N-MBP/3C-site
**24**	BN-BC-C32S-S478C	pETM44 TomBC C36S_S482C	Kan	N-6XHis/N-MBP/3C-site
**25**	BN-BC-I53A	pETM44 TomBC-I57A	Kan	N-6XHis/N-MBP/3C-site
**26**	BN-BC-I80A	pETM44 TomBC-I84A	Kan	N-6XHis/N-MBP/3C-site

**27**	BN-BC-I104A	pETM44 TomBC-I108A	Kan	N-6XHis/N-MBP/3C-site
**28**	BN-BC-I107A	pETM44 TomBC-I111A	Kan	N-6XHis/N-MBP/3C-site
**29**	BN-BC-L159A	pETM44 TomBC-L163A	Kan	N-6XHis/N-MBP/3C-site
**30**	BN-BC-L185A	pETM44 TomBC-L187A	Kan	N-6XHis/N-MBP/3C-site
**31**	BN-BC-V187A	pETM44 TomBC-V191A	Kan	N-6XHis/N-MBP/3C-site
**32**	BN-BC-I221A	pETM44 TomBC-I225A	Kan	N-6XHis/N-MBP/3C-site
**33**	BN-BC-L270A	pETM44 TomBC-L274A	Kan	N-6XHis/N-MBP/3C-site
**34**	BN-BC-L328A	pETM44 TomBC-L332A	Kan	N-6XHis/N-MBP/3C-site
**35**	BN-BC-V334A	pETM44 TomBC-V338A	Kan	N-6XHis/N-MBP/3C-site
**36**	BN-BC-I359A	pETM44 TomBC-I363A	Kan	N-6XHis/N-MBP/3C-site
**37**	BN-BC-V363A	pETM44 TomBC-V367A	Kan	N-6XHis/N-MBP/3C-site
**38**	BN-BC-L365A	pETM44 TomBC-L369A	Kan	N-6XHis/N-MBP/3C-site
**39**	BN-BC-L381A	pETM44 TomBC-L385A	Kan	N-6XHis/N-MBP/3C-site
**40**	BN-BC-I388A	pETM44 TomBC-I392A	Kan	N-6XHis/N-MBP/3C-site
**41**	BN-BC-V394A	pETM44 TomBC-V398A	Kan	N-6XHis/N-MBP/3C-site
**42**	BN-BC-L409A	pETM44 TomBC-L413A	Kan	N-6XHis/N-MBP/3C-site
**43**	BN-BC-I449A	pETM44 TomBC-I453A	Kan	N-6XHis/N-MBP/3C-site
**44**	BN-BC-I485A	pETM44 TomBC-I489A	Kan	N-6XHis/N-MBP/3C-site
**45**	BN-BC-L497A	pETM44 TomBC-L501A	Kan	N-6XHis/N-MBP/3C-site
**46**	BN-BC-L504A	pETM44 TomBC-L508A	Kan	N-6XHis/N-MBP/3C-site
**47**	BN-BC-D11A-H25A	pETM44 TomBC-D15AH29A	Kan	N-6XHis/N-MBP/3C-site
**48**	TomBΔN90	pSTW42 SUMO-TomBdelN95	Kan	N-6XHis/N-SUMO
**49**	TomA	pSTW42 SUMO-TomA	Kan	N-6XHis/N-SUMO
**50**	TomB	pSTW42 SUMO-TomB	Kan	N-6XHis/N-SUMO
**51**	APCP	pETM44-HIPS TomA-PCP	Kan	N-6XHis/N-MBP/3C-site
**52**	AA	pETM44-HIPS TomA-A	Kan	N-6XHis/N-MBP/3C-site
**53**	BN-BC	pETM44-HIPS TomB-C	Kan	N-6XHis/N-MBP/3C-site
**54**	BN	pETM44 TomBC-N95_only	Kan	N-6XHis/N-MBP/3C-site
**55**	BC	pETM44-HIPS TomB-CdelN	Kan	N-6XHis/N-MBP/3C-site
**56**	BA	pETM44-HIPS TomB-A	Kan	N-6XHis/N-MBP/3C-site
**57**	BPCP	pETM44-HIPS TomB-PCP	Kan	N-6XHis/N-MBP/3C-site

REFERENCES AND NOTES

1. R. D. Süssmuth, A. Mainz, Nonribosomal peptide synthesis-principles and prospects. *Angew. Chem. Int. Ed. Engl.* **56**, 3770–3821 (2017).

2. A. S. Brown, M. J. Calcott, J. G. Owen, D. F. Ackerley, Structural, functional and evolutionary perspectives on effective re-engineering of non-ribosomal peptide synthetase assembly lines. *Nat. Prod. Rep.* **35**, 1210–1228 (2018).

3. G. H. Hur, C. R. Vickery, M. D. Burkart, Explorations of catalytic domains in non-ribosomal peptide synthetase enzymology. *Nat. Prod. Rep.* **29**, 1074–1098 (2012).

4. M. A. Marahiel, T. Stachelhaus, H. D. Mootz, Modular peptide synthetases involved in nonribosomal peptide synthesis. *Chem. Rev.* **97**, 2651–2674 (1997).

5. K. Bloudoff, T. M. Schmeing, Structural and functional aspects of the nonribosomal peptide synthetase condensation domain superfamily: Discovery, dissection and diversity. *Biochim. Biophys. Acta Proteins Proteomics* **1865**, 1587–1604 (2017).

6. C. T. Walsh, H. Chen, T. A. Keating, B. K. Hubbard, H. C. Losey, L. Luo, C. G. Marshall, D. A. Miller, H. M. Patel, Tailoring enzymes that modify nonribosomal peptides during and after chain elongation on NRPS assembly lines. *Curr. Opin. Chem. Biol.* **5**, 525–534 (2001).

7. E. J. Drake, B. R. Miller, C. Shi, J. T. Tarrasch, J. A. Sundlov, C. L. Allen, G. Skiniotis, C. C. Aldrich, A. M. Gulick, Structures of two distinct conformations of holo-non-ribosomal peptide synthetases. *Nature* **529**, 235–238 (2016).

8. A. Tanovic, S. A. Samel, L.-O. Essen, M. A. Marahiel, Crystal structure of the termination module of a nonribosomal peptide synthetase. *Science* **321**, 659–663 (2008).

9. J. M. Reimer, M. N. Aloise, P. M. Harrison, T. M. Schmeing, Synthetic cycle of the initiation module of a formylating nonribosomal peptide synthetase. *Nature* **529**, 239–242 (2016).

10. J. Wang, D. Li, L. Chen, W. Cao, L. Kong, W. Zhang, T. Croll, Z. Deng, J. Liang, Z. Wang, Catalytic trajectory of a dimeric nonribosomal peptide synthetase subunit with an inserted epimerase domain. *Nat. Commun.* **13**, 592 (2022).

11. J. M. Reimer, M. Eivaskhani, I. Harb, A. Guarné, M. Weigt, T. M. Schmeing, Structures of a dimodular nonribosomal peptide synthetase reveal conformational flexibility. *Science* **366**, eaaw4388 (2019).

12. M.-J. C. Y. Kee, S. R. Bharath, S. Wee, M. W. Bowler, J. Gunaratne, S. Pan, L. Zhang, H. Song, Structural insights into the substrate-bound condensation domains of non-ribosomal peptide synthetase AmbB. *Sci. Rep.* **12**, 5353 (2022).

13. M. Hahn, T. Stachelhaus, Selective interaction between nonribosomal peptide synthetases is facilitated by short communication-mediating domains. *Proc. Natl. Acad. Sci. U.S.A.* **101**, 15585–15590 (2004).

14. J. Watzel, C. Hacker, E. Duchardt-Ferner, H. B. Bode, J. Wöhnert, A new docking domain type in the peptide-antimicrobial-Xenorhabdus peptide producing nonribosomal peptide synthetase from Xenorhabdus bovienii. *ACS Chem. Biol.* **15**, 982–989 (2020).

15. S. Kosol, A. Gallo, D. Griffiths, T. R. Valentic, J. Masschelein, M. Jenner, E. L. C. de los Santos, L. Manzi, P. K. Sydor, D. Rea, S. Zhou, V. Fülöp, N. J. Oldham, S.-C. Tsai, G. L. Challis, J. R. Lewandowski, Structural basis for chain release from the enacyloxin polyketide synthase. *Nat. Chem.* **11**, 913–923 (2019).

16. J. Watzel, E. Duchardt-Ferner, S. Sarawi, H. B. Bode, J. Wöhnert, Cooperation between a T domain and a minimal C-terminal docking domain to enable specific assembly in a multiprotein NRPS. *Angew. Chem. Int. Ed.* **60**, 14171–14178 (2021).

17. C. Hacker, X. Cai, C. Kegler, L. Zhao, A. K. Weickhmann, J. P. Wurm, H. B. Bode, J. Wöhnert, Structure-based redesign of docking domain interactions modulates the product spectrum of a rhabdopeptide-synthesizing NRPS. *Nat. Commun.* **9**, 4366 (2018).

18. C. D. Richter, D. Nietlispach, R. W. Broadhurst, K. J. Weissman, Multienzyme docking in hybrid megasynthetases. *Nat. Chem. Biol.* **4**, 75–81 (2008).

19. C. D. Fage, S. Kosol, M. Jenner, C. Öster, A. Gallo, M. Kaniusaite, R. Steinbach, M. Staniforth, V. G. Stavros, M. A. Marahiel, M. J. Cryle, J. R. Lewandowski, Communication breakdown: Dissecting the COM interfaces between the subunits of nonribosomal peptide synthetases. *ACS Catal.* **11**, 10802–10813 (2021).

20. D. P. Dowling, Y. Kung, A. K. Croft, K. Taghizadeh, W. L. Kelly, C. T. Walsh, C. L. Drennan, Structural elements of an NRPS cyclization domain and its intermodule docking domain. *Proc. Natl. Acad. Sci. U.S.A.* **113**, 12432–12437 (2016).

21. K. A. J. Bozhueyuek, J. Watzel, N. Abbood, H. B. Bode, Synthetic zippers as an enabling tool for engineering of non-ribosomal peptide synthetases. *Angew. Chem. Int. Ed. Engl.* **60**, 17531–17538 (2021).

22. C. Kegler, H. B. Bode, Artificial splitting of a non-ribosomal peptide synthetase by inserting natural docking domains. *Angew. Chem. Int. Ed.* **59**, 13463–13467 (2020).

23. M. Kaniusaite, R. J. A. Goode, J. Tailhades, R. B. Schittenhelm, M. J. Cryle, Exploring modular reengineering strategies to redesign the teicoplanin non-ribosomal peptide synthetase. *Chem. Sci.* **11**, 9443–9458 (2020).

24. H. G. Smith, M. J. Beech, J. R. Lewandowski, G. L. Challis, M. Jenner, Docking domain-mediated subunit interactions in natural product megasynth(et)ases. *J. Ind. Microbiol. Biotechnol.* **48**, kuab018 (2021).

25. K. Arima, M. Kosaka, G. Tamura, H. Imanaka, H. Sakai, Studies on tomaymycin, a new antibiotic. I. Isolation and properties of tomaymycin. *J. Antibiot. (Tokyo)* **25**, 437–444 (1972).

26. A. von Tesmar, M. Hoffmann, J. Pippel, A. A. Fayad, S. Dausend-Werner, A. Bauer, W. Blankenfeldt, R. Müller, Total biosynthesis of the pyrrolo[4,2]benzodiazepine scaffold tomaymycin on an in vitro reconstituted NRPS system. *Cell Chem. Biol.* **24**, 1216–1227.e8 (2017).

27. W. Li, S. Chou, A. Khullar, B. Gerratana, Cloning and characterization of the biosynthetic gene cluster for tomaymycin, an SJG-136 monomeric analog. *Appl. Environ. Microbiol.* **75**, 2958–2963 (2009).

28. T. A. Keating, C. G. Marshall, C. T. Walsh, A. E. Keating, The structure of VibH represents nonribosomal peptide synthetase condensation, cyclization and epimerization domains. *Nat. Struct. Biol.* **9**, 522–526 (2002).

29. E. Chovancova, A. Pavelka, P. Benes, O. Strnad, J. Brezovsky, B. Kozlikova, A. Gora, V. Sustr, M. Klvana, P. Medek, L. Biedermannova, J. Sochor, J. Damborsky, CAVER 3.0: A tool for the analysis of transport pathways in dynamic protein structures. *PLOS Comput. Biol.* **8**, e1002708 (2012).

30. B. R. Miller, E. J. Drake, C. Shi, C. C. Aldrich, A. M. Gulick, Structures of a nonribosomal peptide synthetase module bound to MbtH-like proteins support a highly dynamic domain architecture. *J. Biol. Chem.* **291**, 22559–22571 (2016).

31. D. F. Kreitler, E. M. Gemmell, J. E. Schaffer, T. A. Wencewicz, A. M. Gulick, The structural basis of N-acyl-α-amino-β-lactone formation catalyzed by a nonribosomal peptide synthetase. *Nat. Commun.* **10**, 3432 (2019).

32. L. Zhong, X. Diao, N. Zhang, F. Li, H. Zhou, H. Chen, X. Bai, X. Ren, Y. Zhang, D. Wu, X. Bian, Engineering and elucidation of the lipoinitiation process in nonribosomal peptide biosynthesis. *Nat. Commun.* **12**, 296 (2021).

33. J. Wen, P. Zhou, J. Wu, Efficient acquisition of high-resolution 4-D diagonal-suppressed methyl–methyl NOESY for large proteins. *J. Magn. Reson.* **218**, 128–132 (2012).

34. K. Bloudoff, D. Rodionov, T. M. Schmeing, Crystal structures of the first condensation domain of CDA synthetase suggest conformational changes during the synthetic cycle of nonribosomal peptide synthetases. *J. Mol. Biol.* **425**, 3137–3150 (2013).

35. S. A. Samel, G. Schoenafinger, T. A. Knappe, M. A. Marahiel, L. O. Essen, Structural and functional insights into a peptide bond-forming bidomain from a nonribosomal peptide synthetase. *Structure* **15**, 781–792 (2007).

36. S. H. Mishra, A. K. Kancherla, K. A. Marincin, G. Bouvignies, S. Nerli, N. Sgourakis, D. P. Dowling, D. P. Frueh, Global protein dynamics as communication sensors in peptide synthetase domains. *Sci. Adv.* **8**, eabn6549 (2022).

37. A. C. Goodrich, B. J. Harden, D. P. Frueh, Solution structure of a nonribosomal peptide synthetase carrier protein loaded with its substrate reveals transient, well-defined contacts. *J. Am. Chem. Soc.* **137**, 12100–12109 (2015).

38. M. J. Jaremko, D. J. Lee, S. J. Opella, M. D. Burkart, Structure and substrate sequestration in the pyoluteorin type II peptidyl carrier protein PltL. *J. Am. Chem. Soc.* **137**, 11546–11549 (2015).

39. C. Shi, B. R. Miller, E. M. Alexander, A. M. Gulick, C. C. Aldrich, Design, synthesis, and biophysical evaluation of mechanism-based probes for condensation domains of nonribosomal peptide synthetases. *ACS Chem. Biol.* **15**, 1813–1819 (2020).

40. J. R. Lai, M. A. Fischbach, D. R. Liu, C. T. Walsh, A protein interaction surface in nonribosomal peptide synthesis mapped by combinatorial mutagenesis and selection. *Proc. Natl. Acad. Sci. U.S.A.* **103**, 5314–5319 (2006).

41. J. Zhang, N. Liu, R. A. Cacho, Z. Gong, Z. Liu, W. Qin, C. Tang, Y. Tang, J. Zhou, Structural basis of nonribosomal peptide macrocyclization in fungi. *Nat. Chem. Biol.* **12**, 1001–1003 (2016).

42. N. Gaitatzis, B. Kunze, R. Müller, In vitro reconstitution of the myxochelin biosynthetic machinery of Stigmatella aurantiaca Sg a15: Biochemical characterization of a reductive release mechanism from nonribosomal peptide synthetases. *Proc. Natl. Acad. Sci. U.S.A.* **98**, 11136–11141 (2001).

43. S. Kozak, L. Lercher, M. N. Karanth, R. Meijers, T. Carlomagno, S. Boivin, Optimization of protein samples for NMR using thermal shift assays. *J. Biomol. NMR* **64**, 281–289 (2016).

44. F. Madeira, M. Pearce, A. R. N. Tivey, P. Basutkar, J. Lee, O. Edbali, N. Madhusoodanan, A. Kolesnikov, R. Lopez, Search and sequence analysis tools services from EMBL-EBI in 2022. *Nucleic Acids Res.* **50**, W276–W279 (2022).

45. X. Robert, P. Gouet, Deciphering key features in protein structures with the new ENDscript server. *Nucleic Acids Res.* **42**, W320–W324 (2014).

46. J. Mistry, S. Chuguransky, L. Williams, M. Qureshi, G. A. Salazar, E. L. L. Sonnhammer, S. C. E. Tosatto, L. Paladin, S. Raj, L. J. Richardson, R. D. Finn, A. Bateman, Pfam: The protein families database in 2021. *Nucleic Acids Res.* **49**, D412–D419 (2021).

47. C. Vonrhein, C. Flensburg, P. Keller, A. Sharff, O. Smart, W. Paciorek, T. Womack, G. Bricogne, Data processing and analysis with the autoPROC toolbox. *Acta Crystallogr. D Biol. Crystallogr.* **67**, 293–302 (2011).

48. W. Kabsch, XDS. *Acta Crystallogr. D Biol. Crystallogr.* **66**, 125–132 (2010).

49. I. J. Tickle, C. Flensburg, P. Keller, W. Paciorek, A. Sharff, C. Vonrhein, G. Bricogne, *STARANISO* (Global Phasing Ltd., 2016).

50. P. Evans, Scaling and assessment of data quality. *Acta Crystallogr. D Biol. Crystallogr.* **62**, 72–82 (2006).

51. P. R. Evans, G. N. Murshudov, How good are my data and what is the resolution? *Acta Crystallogr. D Biol. Crystallogr.* **69**, 1204–1214 (2013).

52. T. C. Terwilliger, P. D. Adams, R. J. Read, A. J. McCoy, N. W. Moriarty, R. W. Grosse-Kunstleve, P. V. Afonine, P. H. Zwart, L. W. Hung, Decision-making in structure solution using Bayesian estimates of map quality: The PHENIX AutoSol wizard. *Acta Crystallogr. D Biol. Crystallogr.* **65**, 582–601 (2009).

53. T. C. Terwilliger, R. W. Grosse-Kunstleve, P. V. Afonine, N. W. Moriarty, P. H. Zwart, L. W. Hung, R. J. Read, P. D. Adams, Iterative model building, structure refinement and density modification with the PHENIX AutoBuild wizard. *Acta Crystallogr. D Biol. Crystallogr.* **64**, 61–69 (2008).

54. P. D. Adams, P. V. Afonine, G. Bunkóczi, V. B. Chen, I. W. Davis, N. Echols, J. J. Headd, L. W. Hung, G. J. Kapral, R. W. Grosse-Kunstleve, A. J. McCoy, N. W. Moriarty, R. Oeffner, R. J. Read, D. C. Richardson, J. S. Richardson, T. C. Terwilliger, P. H. Zwart, PHENIX: A comprehensive Python-

based system for macromolecular structure solution. *Acta Crystallogr. D Biol. Crystallogr.* **66**, 213–221 (2010).

55. P. Emsley, B. Lohkamp, W. G. Scott, K. Cowtan, Features and development of Coot. *Acta Crystallogr. D Biol. Crystallogr.* **66**, 486–501 (2010).

56. A. J. McCoy, R. W. Grosse-Kunstleve, P. D. Adams, M. D. Winn, L. C. Storoni, R. J. Read, Phaser crystallographic software. *J. Appl. Cryst.* **40**, 658–674 (2007).

57. V. Tugarinov, P. M. Hwang, J. E. Ollerenshaw, L. E. Kay, Cross-correlated relaxation enhanced $^{1}H-^{13}C$ NMR spectroscopy of methyl groups in very high molecular weight proteins and protein complexes. *J. Am. Chem. Soc.* **125**, 10420–10428 (2003).

58. D. M. Korzhnev, K. Kloiber, V. Kanelis, V. Tugarinov, L. E. Kay, Probing slow dynamics in high molecular weight proteins by methyl-TROSY NMR spectroscopy: Application to a 723-residue enzyme. *J. Am. Chem. Soc.* **126**, 3964–3973 (2004).

59. M. Ikura, L. E. Kay, R. Tschudin, A. Bax, Three-dimensional NOESY-HMQC spectroscopy of a ^{13}C-labeled protein. *J. Magn. Reson.* **86**, 204–209 (1990).

60. S. W. Fesik, E. R. P. Zuiderweg, Heteronuclear three-dimensional nmr spectroscopy. A strategy for the simplification of homonuclear two-dimensional NMR spectra. *J. Magn. Reson.* **78**, 588–593 (1988).

61. G. M. Clore, L. E. Kay, A. Bax, A. M. Gronenborn, Four-dimensional carbon-13/carbon-13-edited nuclear Overhauser enhancement spectroscopy of a protein in solution: Application to interleukin 1 beta. *Biochemistry* **30**, 12–18 (1991).

62. G. W. Vuister, G. M. Clore, A. M. Gronenborn, R. Powers, D. S. Garrett, R. Tschudin, A. Bax, Increased resolution and improved spectral quality in four-dimensional $^{13}C/^{13}C$-separated HMQC-NOESY-HMQC spectra using pulsed field gradients. *J. Magn. Reson. Ser. B* **101**, 210–213 (1993).

63. S. G. Hyberts, A. G. Milbradt, A. B. Wagner, H. Arthanari, G. Wagner, Application of iterative soft thresholding for fast reconstruction of NMR data non-uniformly sampled with multidimensional Poisson Gap scheduling. *J. Biomol. NMR* **52**, 315–327 (2012).

64. F. Delaglio, S. Grzesiek, G. W. Vuister, G. Zhu, J. Pfeifer, A. Bax, NMRPipe: A multidimensional spectral processing system based on UNIX pipes. *J. Biomol. NMR* **6**, 277–293 (1995).

65. W. F. Vranken, W. Boucher, T. J. Stevens, R. H. Fogh, A. Pajon, M. Llinas, E. L. Ulrich, J. L. Markley, J. Ionides, E. D. Laue, The CCPN data model for NMR spectroscopy: Development of a software pipeline. *Proteins* **59**, 687–696 (2005).

66. G. W. Vuister, A. Bax, Resolution enhancement and spectral editing of uniformly ^{13}C-enriched proteins by homonuclear broadband ^{13}C decoupling. *J. Magn. Reson.* **98**, 428–435 (1992).

67. R. Sprangers, L. E. Kay, Quantitative dynamics and binding studies of the 20S proteasome by NMR. *Nature* **445**, 618–622 (2007).

68. G. Bodenhausen, D. J. Ruben, Natural abundance nitrogen-15 NMR by enhanced heteronuclear spectroscopy. *Chem. Phys. Lett.* **69**, 185–189 (1980).

69. M. Piotto, V. Saudek, V. Sklenář, Gradient-tailored excitation for single-quantum NMR spectroscopy of aqueous solutions. *J. Biomol. NMR* **2**, 661–665 (1992).

70. V. Sklenar, M. Piotto, R. Leppik, V. Saudek, Gradient-tailored water suppression for ^{1}H-^{15}N HSQC experiments optimized to retain full sensitivity. *J. Magn. Reson. Ser. A* **102**, 241–245 (1993).

71. M. Ikura, L. E. Kay, A. Bax, A novel approach for sequential assignment of 1H, 13C, and 15N spectra of proteins: Heteronuclear triple-resonance three-dimensional NMR spectroscopy. Application to calmodulin. *Biochemistry* **29**, 4659–4667 (1990).

72. R. T. Clubb, V. Thanabal, G. Wagner, A constant-time three-dimensional triple-resonance pulse scheme to correlate intraresidue ^{1}H^{N}, ^{15}N, and ^{13}C′ chemical shifts in ^{15}N ^{13}C-labelled proteins. *J. Magn. Reson.* **97**, 213–217 (1992).

73. L. E. Kay, G. Y. Xu, T. Yamazaki, Enhanced-sensitivity triple-resonance spectroscopy with minimal H_2O saturation. *J. Magn. Reson. Ser. A* **109**, 129–133 (1994).

74. S. Grzesiek, A. Bax, Improved 3D triple-resonance NMR techniques applied to a 31 kDa protein. *J. Magn. Reson.* **96**, 432–440 (1992).

75. M. Wittekind, L. Mueller, HNCACB, a high-sensitivity 3D NMR experiment to correlate amide-proton and nitrogen resonances with the alpha- and beta-carbon resonances in proteins. *J. Magn. Reson. Ser. B* **101**, 201–205 (1993).

76. T. Yamazaki, W. Lee, C. H. Arrowsmith, D. R. Muhandiram, L. E. Kay, A suite of triple resonance NMR experiments for the backbone assignment of ^{15}N, ^{13}C, ^{2}H labeled proteins with high sensitivity. *J. Am. Chem. Soc.* **116**, 11655–11666 (1994).

77. A. Bax, M. Ikura, An efficient 3D NMR technique for correlating the proton and ^{15}N backbone amide resonances with the α-carbon of the preceding residue in uniformly $^{15}N/^{13}C$ enriched proteins. *J. Biomol. NMR* **1**, 99–104 (1991).

78. G. T. Montelione, B. A. Lyons, S. D. Emerson, M. Tashiro, An efficient triple resonance experiment using carbon-13 isotropic mixing for determining sequence-specific resonance assignments of isotopically-enriched proteins. *J. Am. Chem. Soc.* **114**, 10974–10975 (1992).

79. T. M. Logan, E. T. Olejniczak, R. X. Xu, S. W. Fesik, Side chain and backbone assignments in isotopically labeled proteins from two heteronuclear triple resonance experiments. *FEBS Lett.* **314**, 413–418 (1992).

80. J. Cavanagh, M. Rance, Suppression of cross-relaxation effects in TOCSY spectra via a modified DIPSI-2 mixing sequence. *J. Magn. Reson.* **96**, 670–678 (1992).

81. A. Bax, G. M. Clore, A. M. Gronenborn, ^{1}H-^{1}H correlation via isotropic mixing of ^{13}C magnetization, a new three-dimensional approach for assigning ^{1}H and ^{13}C spectra of ^{13}C-enriched proteins. *J. Magn. Reson.* **88**, 425–431 (1990).

82. L. E. Kay, G. Y. Xu, A. U. Singer, D. R. Muhandiram, J. D. Formankay, A gradient-enhanced HCCH-TOCSY experiment for recording side-chain ^{1}H and ^{13}C correlations in H_2O samples of proteins. *J. Magn. Reson. Ser. B* **101**, 333–337 (1993).

83. T. Yamazaki, J. D. Forman-Kay, L. E. Kay, Two-dimensional NMR experiments for correlating carbon-13.beta. and proton.delta./.epsilon. chemical shifts of aromatic residues in ^{13}C-labeled proteins via scalar couplings. *J. Am. Chem. Soc.* **115**, 11054–11055 (1993).

84. D. Marion, L. E. Kay, S. W. Sparks, D. A. Torchia, A. Bax, Three-dimensional heteronuclear NMR of nitrogen-15 labeled proteins. *J. Am. Chem. Soc.* **111**, 1515–1517 (1989).

85. D. R. Muhandiram, N. A. Farrow, G. Y. Xu, S. H. Smallcombe, L. E. Kay, A gradient ^{13}C NOESY-HSQC experiment for recording NOESY spectra of ^{13}C-labeled proteins dissolved in H_2O. *J. Magn. Reson. Ser. B* **102**, 317–321 (1993).

86. M.-S. Cheung, M. L. Maguire, T. J. Stevens, R. W. Broadhurst, DANGLE: A Bayesian inferential method for predicting protein backbone dihedral angles and secondary structure. *J. Magn. Reson.* **202**, 223–233 (2010).

87. Y. Shen, A. Bax, Protein backbone and sidechain torsion angles predicted from NMR chemical shifts using artificial neural networks. *J. Biomol. NMR* **56**, 227–241 (2013).

88. W. Rieping, M. Habeck, B. Bardiaux, A. Bernard, T. E. Malliavin, M. Nilges, ARIA2: Automated NOE assignment and data integration in NMR structure calculation. *Bioinformatics* **23**, 381–382 (2007).

89. A. T. Brunger, Version 1.2 of the Crystallography and NMR system. *Nat. Protoc.* **2**, 2728–2733 (2007).

90. A. W. Schuttelkopf, D. M. F. van Aalten, PRODRG: A tool for high-throughput crystallography of protein-ligand complexes. *Acta Crystallogr. D* **60**, 1355–1363 (2004).

91. V. Tugarinov, L. E. Kay, Relaxation rates of degenerate ^{1}H transitions in methyl groups of proteins as reporters of side-chain dynamics. *J. Am. Chem. Soc.* **128**, 7299–7308 (2006).

92. V. Tugarinov, L. E. Kay, Estimating side-chain order in [U-^{2}H;$^{13}CH_3$]-labeled high molecular weight proteins from analysis of HMQC/HSQC spectra. *J. Phys. Chem. B* **117**, 3571–3577 (2013).

93. J. Iwahara, C. D. Schwieters, G. M. Clore, Ensemble aqpproach for NMR structure refinement against ^{1}H paramagnetic relaxation enhancement data arising from a flexible paramagnetic group attached to a macromolecule. *J. Am. Chem. Soc.* **126**, 5879–5896 (2004).

94. C. D. Schwieters, J. J. Kuszewski, N. Tjandra, G. Marius Clore, The Xplor-NIH NMR molecular structure determination package. *J. Magn. Reson.* **160**, 65–73 (2003).

95. C. Dominguez, R. Boelens, A. M. J. J. Bonvin, HADDOCK: A protein−protein docking approach based on biochemical or biophysical information. *J. Am. Chem. Soc.* **125**, 1731–1737 (2003).

96. A. Šali, T. L. Blundell, Comparative protein modelling by satisfaction of spatial restraints. *J. Mol. Biol.* **234**, 779–815 (1993).

97. C. J. Williams, J. J. Headd, N. W. Moriarty, M. G. Prisant, L. L. Videau, L. N. Deis, V. Verma, D. A. Keedy, B. J. Hintze, V. B. Chen, S. Jain, S. M. Lewis, W. B. Arendall Iii, J. Snoeyink, P. D. Adams, S. C. Lovell, J. S. Richardson, D. C. Richardson, MolProbity: More and better reference data for improved all-atom structure validation. *Protein Sci.* **27**, 293–315 (2018).

98. Schrödinger LLC, *The PyMOL Molecular Graphics System, Version 2.5* (Schrödinger LLC, 2021).

99. R. A. Laskowski, PDBsum1: A standalone program for generating PDBsum analyses. *Protein Sci.* **31**, e4473 (2022).
